# Drug-induced phospholipidosis confounds drug repurposing for SARS-CoV-2

**DOI:** 10.1126/science.abi4708

**Published:** 2021-07-30

**Authors:** Tia A. Tummino, Veronica V. Rezelj, Benoit Fischer, Audrey Fischer, Matthew J. O’Meara, Blandine Monel, Thomas Vallet, Kris M. White, Ziyang Zhang, Assaf Alon, Heiko Schadt, Henry R. O’Donnell, Jiankun Lyu, Romel Rosales, Briana L. McGovern, Raveen Rathnasinghe, Sonia Jangra, Michael Schotsaert, Jean-René Galarneau, Nevan J. Krogan, Laszlo Urban, Kevan M. Shokat, Andrew C. Kruse, Adolfo García-Sastre, Olivier Schwartz, Francesca Moretti, Marco Vignuzzi, Francois Pognan, Brian K. Shoichet

**Affiliations:** 1 Department of Pharmaceutical Chemistry, University of California San Francisco (UCSF), San Francisco, CA, USA.; 2 Graduate Program in Pharmaceutical Sciences and Pharmacogenomics, UCSF, San Francisco, CA, USA.; 3 Quantitative Biosciences Institute (QBI), UCSF, San Francisco, CA, USA.; 4 QBI COVID-19 Research Group (QCRG), San Francisco, CA, USA.; 5 Institut Pasteur, Viral Populations and Pathogenesis Unit, CNRS UMR 3569, 75724 Paris, Cedex 15, France.; 6 Novartis Institutes for BioMedical Research, Preclinical Safety, Basel, Switzerland.; 7 Department of Computational Medicine and Bioinformatics, University of Michigan, Ann Arbor, MI, USA.; 8 Institut Pasteur, Virus and Immunity Unit, CNRS UMR 3569, 75724 Paris, Cedex 15, France.; 9 Department of Microbiology, Icahn School of Medicine at Mount Sinai, New York, NY, USA.; 10 Global Health and Emerging Pathogens Institute, Icahn School of Medicine at Mount Sinai, New York, NY, USA.; 11 Department of Cellular and Molecular Pharmacology, UCSF, San Francisco, CA, USA.; 12 Howard Hughes Medical Institute, UCSF, San Francisco, CA, USA.; 13 Department of Biological Chemistry and Molecular Pharmacology, Blavatnik Institute, Harvard Medical School, Boston, MA, USA.; 14 Graduate School of Biomedical Sciences, Icahn School of Medicine at Mount Sinai, New York, NY, USA.; 15 Novartis Institutes for BioMedical Research, Preclinical Safety, Cambridge, MA, USA.; 16 Gladstone Institute of Data Science and Biotechnology, J. David Gladstone Institutes, San Francisco, CA, USA.; 17 Department of Medicine, Division of Infectious Diseases, Icahn School of Medicine at Mount Sinai, New York, NY, USA.; 18 Tisch Cancer Institute, Icahn School of Medicine at Mount Sinai, New York, NY, USA.

## Abstract

Repurposing drugs as treatments for COVID-19, the disease caused by severe acute respiratory syndrome coronavirus 2 (SARS-CoV-2), has drawn much attention. Beginning with sigma receptor ligands and expanding to other drugs from screening in the field, we became concerned that phospholipidosis was a shared mechanism underlying the antiviral activity of many repurposed drugs. For all of the 23 cationic amphiphilic drugs we tested, including hydroxychloroquine, azithromycin, amiodarone, and four others already in clinical trials, phospholipidosis was monotonically correlated with antiviral efficacy. Conversely, drugs active against the same targets that did not induce phospholipidosis were not antiviral. Phospholipidosis depends on the physicochemical properties of drugs and does not reflect specific target-based activities—rather, it may be considered a toxic confound in early drug discovery. Early detection of phospholipidosis could eliminate these artifacts, enabling a focus on molecules with therapeutic potential.

## Screening for drugs that don’t work

In the battle against COVID-19, drugs discovered in repurposing screens are of particular interest because these could be rapidly implemented as treatments. However, Tummino *et al*. deliver a cautionary tale, finding that many leads from such screens have an antiviral effect in cells through phospholipidosis, a phospholipid storage disorder that can be induced by cationic amphiphilic drugs (see the Perspective by Edwards and Hartung). There is a strong correlation between drug-induced phospholipidosis and inhibition of severe acute respiratory syndrome coronavirus 2 replication in cells. Unfortunately, drugs that have an antiviral effect in cells through phospholipidosis are unlikely to be effective in vivo. Screening out such drugs may allow a focus on drugs with better clinical potential. —VV

## Teaser

Phospholipidosis is a shared mechanism underlying the in vitro antiviral activity of many repurposed drugs against SARS-CoV-2.

**Fig. 1.**
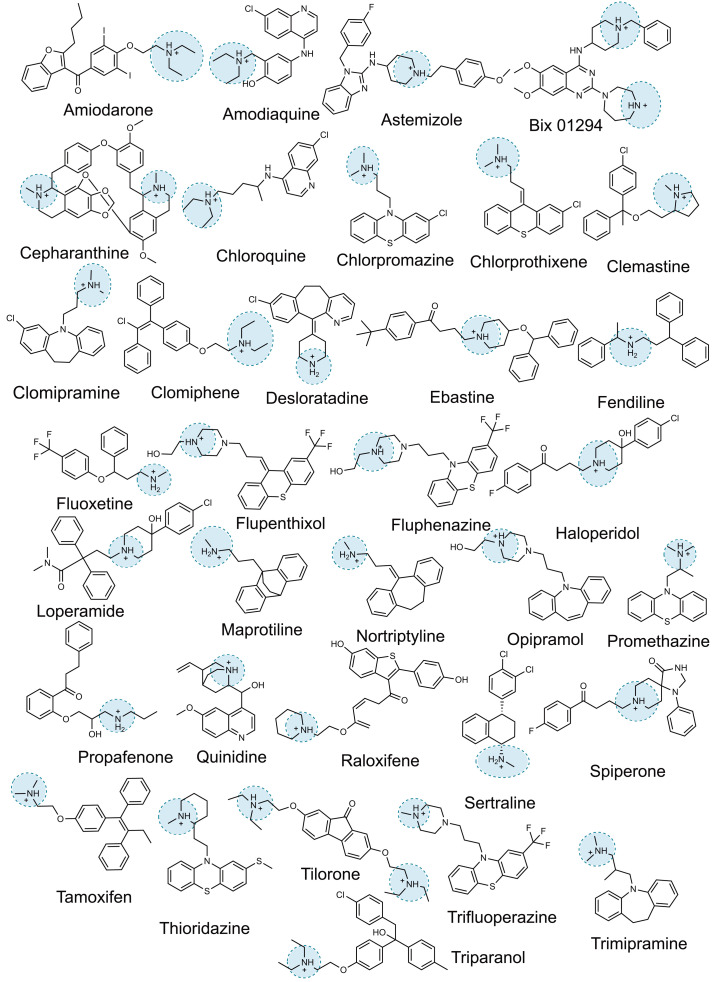
Representative examples of CADs that are identified in SARS-CoV-2 drug repurposing screens.

The outbreak of COVID-19 has inspired multiple drug repurposing screens to find antiviral therapeutics that can be rapidly brought to the clinic (*1*). To date, more than 1974 drugs and investigational drugs have been reported to have in vitro activity against severe acute respiratory syndrome coronavirus 2 (SARS-CoV-2) (*1*) (Fig. 1). Because almost all of these drugs act against human targets and might be unlikely to be viable against a novel virus (*2*), the question of mechanism of action arises.

Our interest in this question was motivated by the discovery that human sigma receptors were candidates for modulating SARS-CoV-2 infection (*3*) and that drugs and reagents like chloroquine, haloperidol, clemastine, and PB28—all with nanomolar affinity against one or both sigma receptors—had cellular antiviral half-maximal inhibitory concentration (IC_50_) values in the 300-nM to 5-μM range. Subsequently, we investigated more than 50 different molecules with a wide range of affinities at these receptors. Although we found molecules with relatively potent antiviral activity, there was little correlation between receptor potency and antiviral efficacy in cells (figs. S1 to S3 and table S1). Whereas drugs like amiodarone, sertraline, and tamoxifen had mid- to high-nanomolar antiviral IC_50_s, other potent sigma ligands, such as melperone and ditolylguanidine (DTG), were without measurable antiviral activity. Notably, the antiviral sigma drugs were all cationic at physiological pH and relatively hydrophobic, whereas those that were inactive against the virus were often smaller and more polar. This cationic-amphiphilic character was shared by many of the hits emerging from other phenotypic screens (Fig. 1 and fig. S4), suggesting that it was this physicochemical property that might explain cellular antiviral activity instead of a specific on-target activity (*4*).

If the cationic-amphiphilic nature of these molecules led to antiviral activity in vitro, rather than their target-based activities, one would expect this physical property to reflect a shared cellular mechanism. In fact, cationic amphiphilic drugs (CADs) can provoke phospholipidosis in cells and organs (*5*). This side effect is characterized by the formation of vesicle-like structures and “foamy” or “whorled” membranes (*5*, *6*) and is thought to arise by CAD disruption of lipid homeostasis. CADs accumulate in intracellular compartments, such as endosomes and lysosomes, where they can directly or indirectly inhibit lipid processing (*5*). Modulation of these same lipid processing pathways is critical for viral replication (*7*), and inhibiting phospholipid production has previously been associated with the inhibition of coronavirus replication (*8*). CADs have in vitro activity against multiple viruses including severe acute respiratory syndrome, Middle East respiratory syndrome, Ebola, Zika, dengue, and filoviruses (*9*), though CAD induction of phospholipidosis has only been proposed as an antiviral mechanism for Marburg virus (*10*). Finally, among the most potent known phospholipidosis inducers are amiodarone (*11*) and chloroquine (*12*, *13*), which are also potent inhibitors of SARS-CoV-2 replication in vitro (*14*–*16*), whereas drugs from SARS-CoV-2 phenotypic screens, such as chlorpromazine (*17*) and tamoxifen (*16*), are also known to induce phospholipidosis (*18*). As an effect that rarely occurs at concentrations lower than 100 nM, which does not appear to translate from in vitro to in vivo antiviral activity and which can result in dose-limiting toxicity (*19*), phospholipidosis may be a confound to true antiviral drug discovery.

Here, we investigate the association between phospholipidosis and antiviral activity against SARS-CoV-2 in cell culture. This apparently general mechanism may be responsible for many of the drug repurposing hits for SARS-CoV-2 and an extraordinary amount of effort and resources lavished on drug discovery against this disease. We explore the prevalence of this confound in SARS-CoV-2 repurposing studies, how phospholipidosis correlates with inhibition of viral infection, and how to eliminate such hits rapidly so as to focus on drugs with genuine potential against COVID-19 and against pandemics yet to arise.

## Results

### Correlation of phospholipidosis and antiviral activity

**Fig. 2.**
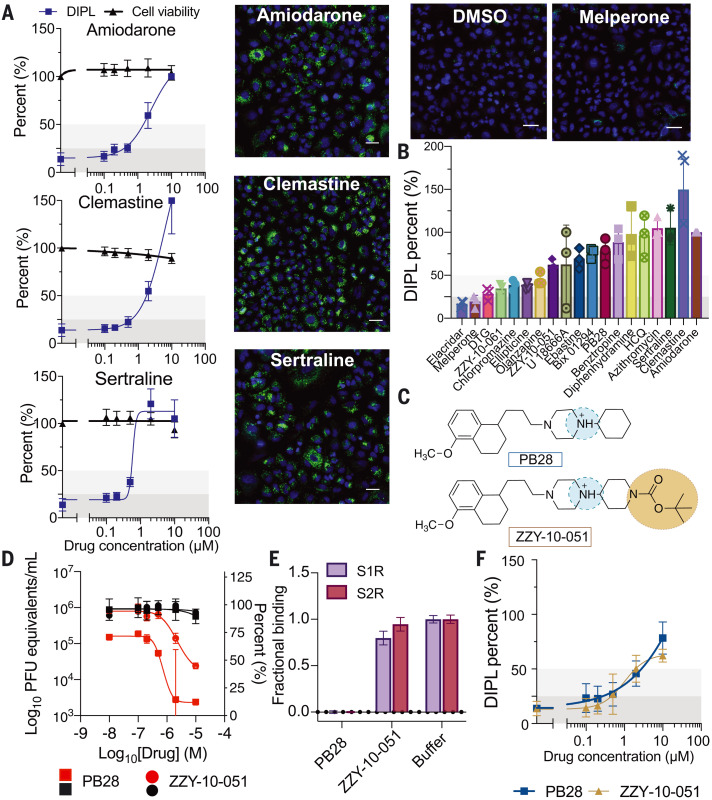
Cellular phospholipidosis may confound antiviral screening results. (**A**) Examples of NBD-PE quantification of phospholipidosis in A549 cells, including dose-response curves. Blue indicates Hoechst nuclei staining, and green indicates NBD-PE phospholipid staining. Scale bars, 20 μm. Amiodarone is the positive control for assay normalization; sertraline and clemastine are two examples of high phospholipidosis-inducing drugs [phospholipidosis (DIPL) > 50% of amiodarone]. Images of DMSO and a non–phospholipidosis-inducing molecule (melperone) are included for reference. Thresholds for determining phospholipidosis power are shaded in dark gray (low phospholipidosis), light gray (medium phospholipidosis), and no shading (high phospholipidosis). (**B**) Pooled DIPL amounts (means ± SDs) at the highest nontoxic concentration tested for each drug. Results were pooled from three biological and three technical replicates and were normalized to amiodarone (100%) from the control wells in the same experimental batches. (**C**) Structures of PB28 and its analog ZZY-10-051, the latter of which is inactive on the sigma receptors. (**D**) Viral infectivity (red) and viability (black) data for PB28 (squares) and ZZY-10-051 (circles) in A549-ACE2 cells. Data shown are means ± SDs from three technical replicates. (**E**) Fractional binding of PB28 and ZZY-10-051 against sigma-1 (purple; S1R) and sigma-2 (maroon; S2R) normalized to a buffer control at 1.0 in a radioligand binding experiment. Data shown are means ± SEMs from three technical replicates. PB28 is a strong ligand of both sigma-1 and sigma-2 and has high displacement of the radioligands, whereas ZZY-10-051 is unable to displace the radioligands to a high degree at 1 μM. (**F**) Dose-response curves for PB28 (blue) and ZZY-10-051 (gold) show that these closely related analogs both induce phospholipidosis.

To investigate the role of phospholipidosis in antiviral activity in vitro, we tested 19 drugs for their induction of this effect in A549 cells using the well-established nitrobenzoxadiazole–conjugated phosphoethanolamine (NBD-PE) staining assay (*20*). Here, the vesicular lipidic bodies characteristic of the effect may be quantified by high-content imaging (Fig. 2A). Three classes of drugs and reagents were initially investigated: (i) sigma-binding antiviral CADs we had discovered, like amiodarone, sertraline, chlorpromazine, and clemastine (nine total), which are predicted or known to induce phospholipidosis; (ii) analogs of these CADs that no longer bound sigma receptors but were still antiviral (four total), which are predicted to induce phospholipidosis despite their lack of sigma binding; and (iii) sigma-binding, nonantiviral drugs, like melperone and DTG, that are more polar than classic CADs (two total), which are predicted not to induce phospholipidosis. Of the nine sigma-binding CADs that were antiviral (the first class)—six of which were also reported in phenotypic screens in the literature as inhibitors of SARS-CoV-2—eight induced phospholipidosis consistent with the hypothesis (Fig. 2, A and B, and figs. S5 and S6). The only non–phospholipidosis-inducing antiviral from this set was elacridar, a promiscuous P-glycoprotein inhibitor; this drug may therefore be active through another mechanism. Notably, analogs of the potent sigma ligand PB28 that had lost their sigma-binding activity but remained CADs (ZZY-10-051 and ZZY-10-061; Fig. 2, B to F, and figs. S5 to S8) did induce phospholipidosis, as did the antipsychotic olanzapine and the antihistamine diphenhydramine, which are weak sigma receptor ligands but are structurally related to potent sigma receptor ligands like chlorpromazine and clemastine. Finally, melperone and DTG, which are potent cationic sigma receptor ligands but are not antiviral, did not induce phospholipidosis (Fig. 2, A and B, and figs. S5 and S6; class iii). These results do not prove phospholipidosis as the antiviral mechanism but are consistent with the phospholipidosis hypothesis.

If phospholipidosis is responsible for antiviral activity, then other molecules known to induce phospholipidosis should be antiviral. We tested three CADs for antiviral activity, including ebastine, ellipticine, and Bix01294, all of which are reported to induce phospholipidosis (*21*) [Bix01294 and ebastine have also been reported as drug repurposing hits against SARS-CoV-2 (*22*)]. We further tested azithromycin, which is also reported to induce phospholipidosis (*23*) but has different physical properties than typical CADs. We first confirmed phospholipidosis-inducing activity for these molecules (Fig. 2B and figs. S5 and S6). All four molecules were next shown to be antiviral using live virus assays (e.g., SARS-CoV-2 strain BetaCoV/France/IDF0372/2020; see materials and methods), with IC_50_ values in the 400-nM to 3-μM range, overlapping with the activities of other CADs that we and others have identified for SARS-CoV-2 (*22*) (fig. S6). This too was consistent with the antiviral phospholipidosis hypothesis.

**Fig. 3.**
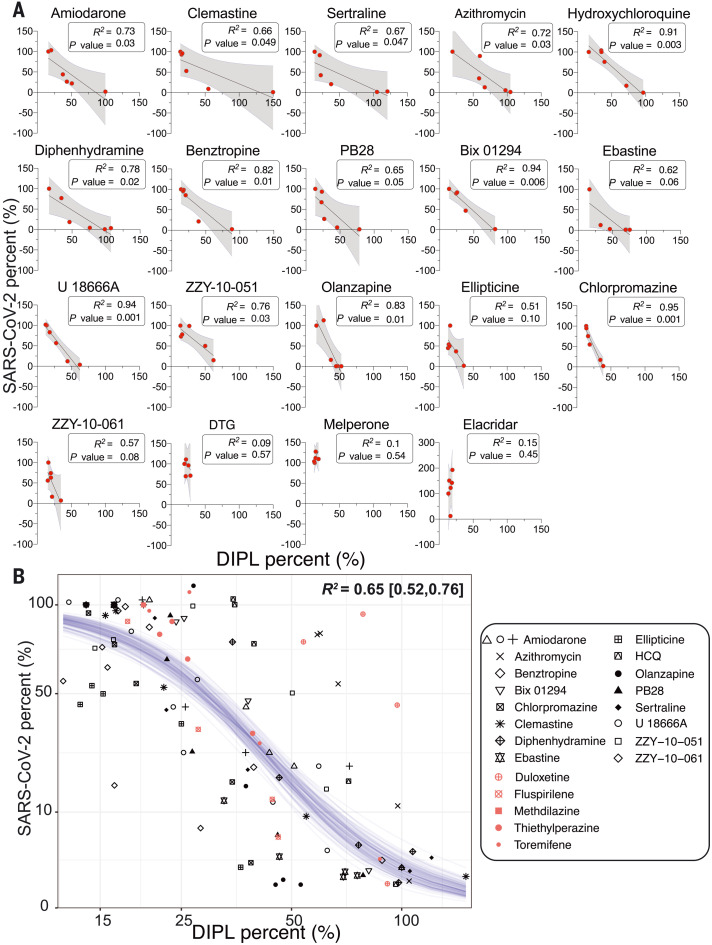
Quantitative relationship between phospholipidosis and viral amounts. (**A**) Correlations between phospholipidosis (DIPL), normalized to amiodarone at 100%, and percent of SARS-CoV-2, normalized to DMSO at 100%, in the reverse transcription quantitative polymerase chain reaction (RT-qPCR) assay in A549-ACE2 cells. Each dot represents the same concentration tested in both assays. A strong negative correlation emerges, with *R*^2^ ≥ 0.65 and *P* ≤ 0.05 for all high and medium phospholipidosis-inducing drugs except ellipticine, which is confounded by its cytotoxicity in both experiments; ebastine; and ZZY-10-61. The latter two examples are marginally significant. (**B**) The SARS-CoV-2 viral loads and induced phospholipidosis magnitude for each compound and dose in (A) are plotted as sqrt(viral_amount_mean) ~ 10 × inv_logit{hill × 4/10 × [log(DIPL_mean) − logIC_50_]}. Fitting a sigmoid Bayesian model with weakly informative priors yields parameters and 95% credible intervals of IC_50_ = 43 (38, 48)%, hill: −5.6 (−7.0, −4.5), and sigma 2.0 (0.14, 1.78). Forty draws from the fit model are shown as blue lines. Salmon-colored points overlaid with the model represent predicted phospholipidosis inducers from the literature (fig. S10).

For phospholipidosis to explain antiviral activity, we might expect a correlation between concentration-response curves for phospholipidosis and for antiviral activity. We compared concentrations that induce phospholipidosis with those that inhibit SARS-CoV-2 for each drug individually. Most correlations were high—not only did antiviral activity occur in the same concentration ranges as phospholipidosis, but *R*^2^ (coefficient of determination) values, ranging from 0.51 to 0.94, supported a quantitative relationship between the two effects (Fig. 3A). We then fit a sigmoidal model through all of the 107 phospholipidosis versus antiviral activity observations (made up of six concentration measurements for each of the 16 phospholipidosis-inducing drugs) and observed a strong negative correlation [*R*^2^ = 0.65; 95% confidence interval (95% CI), 0.52 to 0.76] between induced phospholipidosis and SARS-CoV-2 viral load across all observations for all 16 drugs. Because phospholipidosis and antiviral effects are both saturable, the sigmoidal curve–fit plateaus at the extremes (Fig. 3B).

### Concurrent measurement of viral infection and drug-induced phospholipidosis

**Fig. 4.**
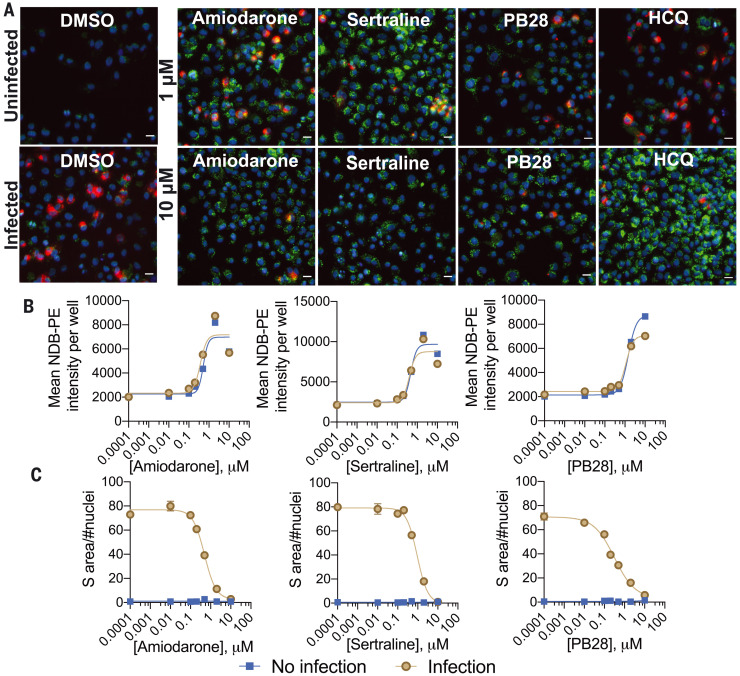
Phospholipidosis and spike protein measurements in the same cellular context. (**A**) Representative images from a costaining experiment measuring phospholipidosis and SARS-CoV-2 spike protein in infected and uninfected A549-ACE2 cells. Five molecules (1 and 10 μM) and DMSO were measured; see fig. S9 for Bix 01294. Blue indicates Hoechst nuclei staining, green indicates NBD-PE phospholipid staining, red indicates SARS-CoV-2 spike protein staining, and yellow indicates coexpression of spike protein and NBD-PE. Scale bar, 20 μm. (**B**) Concentration-response curves for phospholipidosis induction measured by NBD-PE staining in infected cells for three characteristic CADs. (**C**) Spike protein in infected cells decreases as phospholipidosis increases. For (B) and (C), data are means ± SEMs from four biological replicates.

In the previous experiments, drug-induced phospholipidosis and drug antiviral activity were measured separately. To measure the two effects in the same cells at the same time, we dosed cells with either 1 or 10 μM of five characteristic CADs [amiodarone, sertraline, PB28, hydroxychloroquine (HCQ), and Bix01294], followed by a mock or SARS-CoV-2 infection, and quantified phospholipidosis and the accumulation of viral spike protein (Fig. 4A and fig. S9). Compared with dimethyl sulfoxide (DMSO), drug treatments led to substantial increases in NBD-PE aggregates, indicating increased phospholipidosis (fig. S9). At 1-μM drug concentrations, SARS-CoV-2 spike protein was readily stained, and it was possible to visualize both spike protein and phospholipidosis in the same cells (yellow puncta), which suggests that at this low concentration of drug—often close to the antiviral IC_50_ value—both phospholipidosis and viral infection co-occur, though even viral staining was reduced relative to that observed in the DMSO-treated controls. As drug concentration rose to 10 μM, viral spike protein staining dropped, whereas staining for phospholipidosis increased (fig. S9); there was nearly complete loss of spike protein signal with a concomitant increase in phospholipidosis (Fig. 4A) for all treatments. In seven-point concentration-response curves for amiodarone, sertraline, and PB28, viral staining monotonically decreased as phospholipidosis increased (Fig. 4, B and C).

### CADs are common among drug repurposing hits for SARS-CoV-2 and other viruses

With the strong correlation between CAD phospholipidosis and antiviral efficacy (Fig. 3), including drugs that have been found in multiple SARS-CoV-2 repurposing studies, we investigated the prevalence of phospholipidosis-inducing CADs among 1974 repurposing hits identified in the literature. We focused on 12 studies, including two screens of the ReFRAME library (*24*, *25*) and screens of the National Center for Advancing Translational Sciences (NCATS) “approved drug” and “bioactive” libraries (*15*), among others (*3*, *14*, *16*, *22*, *26*–*30*). Together, these 12 screens found 310 drugs, investigational drugs, and reagents that were antiviral in vitro against SARS-CoV-2. We used two physicochemical features to identify likely CADs: drugs with calculated log octanol:water coefficients above 3 (cLogP ≥ 3) and with p*K*_a_ values (where *K*_a_ is the acid dissociation constant) ≥7.4 (*31*, *32*). We then further filtered for drugs that topologically resembled known phospholipidosis inducers (*18*, *21*) using an ECFP4-based Tanimoto coefficient (Tc) ≥0.4 (table S2). Of the 310 drugs, 60% passed the cLogP and p*K*_a_ threshold, and 34% also resembled a known phospholipidosis inducer (Fig. 1 and figs. S4 and S10).

Although the two physical property filters do not capture atypical phospholipidosis inducers such as azithromycin, they do capture 16 of the other 18 CADs we had already tested (missing only the medium phospholipidosis inducers, olanzapine and ellipticine). Notably, nine of these, including amiodarone, sertraline, chlorpromazine, Bix01294, clemastine, and benztropine, also appeared in at least one of the 12 other repurposing studies. To probe the reliability of this association, we tested another five drugs that passed our filters and had been reported as antiviral against SARS-CoV-2 for their induction of phospholipidosis. Not only were all five active in the NBD-PE assay, but we confirmed SARS-CoV-2 antiviral activity for these drugs (fig. S10). Additionally, these molecules fit into the sigmoidal model relating phospholipidosis to viral load (salmon-colored points overlaid with sigmoidal model; Fig. 3B). Finally, we note a preliminary identification of 30 CADs, 19 of which overlap with the literature-derived SARS-CoV-2 list, active against other viruses including Middle East respiratory syndrome and severe acute respiratory syndrome (*33*), Ebola (*34*–*36*), Marburg (*36*, *37*), hepatitis C (*38*), and dengue (*39*) (table S3). It may be that most drugs repurposed against many viruses are CADs, whose antiviral activities can be attributed to phospholipidosis.

### Animal efficacy for repurposed drugs

**Fig. 5.**
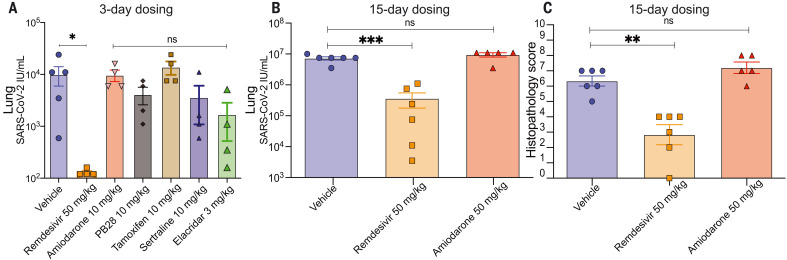
Phospholipidosis-inducing drugs are not efficacious in vivo. (**A**) Three-day dosing of six different drugs with a 2-hour preincubation before SARS-CoV-2 treatment. Lung viral titers were quantified, and groups were compared using the Kruskal-Wallis test [*H*(7) = 22.76; *P* = 0.002] with Dunn’s multiple comparison correction indicated (vehicle *N* = 5; remdesivir *N* = 4; *P = 0.02). All other groups, *N* = 4. ns, not significant. (**B**) Fifteen-day dosing of amiodarone (50 mg/kg) compared with 3-day remdesivir dosing. Lung viral titers were quantified, and groups were compared with a two-way analysis of variance (ANOVA) [main effect of treatment *F*(2,9) = 19.66, *P* = 0.0005; no main effect of mouse, *F*(5,9) = 1.21, *P* = 0.38]. Individual group comparisons determined using Dunnett’s multiple comparison test are indicated (vehicle *N* = 6; remdesivir *N* = 6; ***P = 0.0008; amiodarone *N* = 5; ns, not significant). (**C**) Histopathology scores after 15-day (amiodarone) or 3-day (remdesivir) treatments as in (B). See materials and methods for scoring breakdown. Groups were compared with a two-way ANOVA [main effect of treatment *F*(2,9) = 19.05, *P* = 0.0006; no main effect of mouse, *F*(5,9) = 0.78, *P* = 0.59]. Individual group comparisons determined using Dunnett’s multiple comparison test are indicated (vehicle *N* = 6; remdesivir *N* = 6; **P = 0.0014; amiodarone *N* = 5; ns, not significant). All data are means ± SEMs.

Although phospholipidosis is considered a drug-induced side effect, it remains possible that it can be leveraged for antiviral efficacy. Accordingly, we tested four of the repurposed, phospholipidosis-inducing drugs most potent against SARS-CoV-2 in vitro—amiodarone, sertraline, PB28, and tamoxifen (*5*, *18*)—for efficacy in a murine model of COVID-19 (*40*). In the same model, we also tested elacridar, which does not induce phospholipidosis (Fig. 2B), and remdesivir, which is unlikely to induce phospholipidosis at concentrations relevant to its antiviral activity. All molecules had relatively long half-lives, especially in the lung, where tissue maximum concentration (C_max_) values often exceeded 10 μM after a 10 mg/kg dose, or were 10 to 1000 times as high as their in vitro antiviral IC_50_, which suggests that exposure would be high enough for efficacy (tables S4 to S8). Guided by the pharmacokinetics of each drug, mice were dosed either once (amiodarone and elacridar) or twice per day (remdesivir, PB28, tamoxifen, and sertraline) for 3 days. Two hours after the first dose, mice were intranasally infected with 1 × 10^4^ plaque-forming units (PFU) of SARS-CoV-2, and lung viral titers were measured after a 3-day infection period. Notwithstanding their high lung exposure, the four phospholipidosis-inducing drugs had little effect on viral propagation in the mice. Conversely, remdesivir reduced viral load by two to three orders of magnitude. Although the cationic nonphospholipidosis drug elacridar had a modest antiviral effect, it did not rise to statistical significance (Fig. 5), and mice given elacridar doses higher than 3 mg/kg exhibited toxicities that limited further study.

Because phospholipidosis is typically an in vivo side effect that appears after chronic dosing, we then pretreated mice with fivefold higher concentrations (50 mg/kg) of amiodarone over the course of 12 days before a 3-day infection period. Even in this case, no diminution of viral titer was observed in mouse lungs after infection, and amiodarone offered no protection from infection-induced weight loss or from pulmonary inflammation and epithelial necrosis, as measured by histopathology (Fig. 5 and fig. S11). We note that foamy vacuolation and whorled vacuoles that are the hallmarks of phospholipidosis were not seen in lung and spleen by light or transmission electron microscopy. It is thus possible that this treatment was not long enough to induce protective phospholipidosis. Still, the in vitro activities of the phospholipidosis-inducing drugs did not readily translate in vivo, and drugs whose antiviral activity arises as a result of phospholipidosis seem nonviable for clinical progression.

## Discussion

The emergence of COVID-19 has motivated intense effort to repurpose drugs as SARS-CoV-2 antivirals. An extraordinary number of diverse, apparently unrelated hits have emerged (*1*). A key observation from this work is that many, perhaps most, of these are active in antiviral assays through the induction of phospholipidosis (Fig. 1 and figs. S4 and S10). This disrupts lysosomal lipid catabolism and trafficking, which may in turn disrupt the double membrane vesicles that the virus creates and on which it depends for propagation. Quantitatively, there is a close in vitro correlation between drug-induced phospholipidosis and antiviral activity, both drug-by-drug and over the set of drugs tested in this work (Fig. 3). The effect is predictive: Molecules that induce phospholipidosis are antiviral over the same concentration range, irrespective of whether they are CADs or not (e.g., azithromycin), whereas molecules that are related by target activity to the CADs, but are more polar and do not induce phospholipidosis (e.g., melperone and DTG), are not antiviral. Unfortunately, CAD induction of phospholipidosis—at least at the potencies observed in this work—does not translate in vivo (Fig. 5). More encouragingly, this study illuminates a method to rapidly identify confounds in cellular antiviral screens, allowing us to eliminate them from further study and to focus on molecules with true potential.

Although the molecular mechanisms for the antiviral effects of phospholipidosis remain unclear, certain associations may be tentatively advanced. SARS-CoV-2, like many viruses, subverts the cell to produce double membrane vesicles in which it replicates (*41*–*43*). Disruption of lipid homeostasis by the induction of phospholipidosis may disrupt these vesicles, reducing viral replication. The disruption of lysosomal (*44*) and endosomal (*45*) compartments and CAD-induced shifts in compartmental pH (*46*) may further affect viral entry and propagation (*47*). Accordingly, targeting the endosomal-lysosomal pathway has been suggested as a viable strategy against SARS-CoV-2 infection (*48*), but developing potent and targeted inhibitors remains challenging.

The cost to the community of investments in what appears to be a confound merits consideration for future pandemics. According to the DrugBank COVID-19 dashboard (*49*), which draws from US and international clinical trials, putatively antiviral CADs have been promoted into an astonishing 316 phase 1 to phase 3 clinical trials against COVID-19. Although 57% of these trials study the phospholipidosis-inducing CADs hydroxychloroquine (Fig. 3A, top row) or chloroquine, that still leaves 136 trials across 33 other predicted or known phospholipidosis inducers. Using conservative estimates (*50*, *51*), the expense of the clinical trials component alone over the last year for phospholipidosis-inducing CADs may be over $6 billion US dollars (table S9).

Certain caveats merit mentioning. First, the correlation between antiviral activity and phospholipidosis, as strong as it is, does not illuminate the mechanism by which phospholipidosis is antiviral. Phospholipidosis is itself only partly understood, and there are no good genetic or chemical ways to either inhibit its induction by drugs nor to promote it by target-selective reagents. Second, predicting whether a molecule will induce phospholipidosis remains challenging, and even non-CAD molecules can induce it. Thus, we have chosen conservative criteria to predict phospholipidosis inducers, which may miss many drugs. Third, phospholipidosis is a confound that only affects drugs repurposed for direct antiviral activity—it is irrelevant for drugs like dexamethasone (*52*) and fluvoxamine (*53*) that have been repurposed for immunomodulation in COVID-19, and it is also irrelevant for CADs whose antiviral activity is well below the concentration range where phospholipidosis occurs. Fourth, our estimates of the clinical trial costs of phospholipidosis-inducing CADs are obviously rough. Finally, we do not exclude exploiting phospholipidosis therapeutically, although we suspect that would have to proceed through a more target-directed mechanism than that of the CADs studied here.

These caveats should not obscure the central observation of this study. Many drugs repurposed for antiviral activity against SARS-CoV-2 are cationic amphiphiles, and, despite their diverse structures and multiple targets, many likely have their antiviral effects through a single shared mechanism: phospholipidosis. Both because of the side effects with which it is associated and the limited efficacy to which it leads—rarely better than 100 nM in vitro—drugs active as a result of phospholipidosis are unlikely to translate in vivo (Fig. 5). Many resources will be saved by counterscreening for phospholipidosis in simple cellular assays (*20*), allowing investigators to focus on drugs with genuine promise as antivirals.

## Supplementary Material

20210622-1

## Supplementary Materials

science.sciencemag.org/content/373/6554/541/suppl/DC1
Materials and Methods Figs. S1 to S11 Tables S1 to S9 References (*54*–*66*) MDAR Reproducibility Checklist

## Appendix

View/request a protocol for this paper from *Bio-protocol*.
