# Supplementary material for: Drug-induced phospholipidosis confounds drug repurposing for SARS-CoV-2
Source: Science. 2021 Jun 22;373(6554):541–7. doi: 10.1126/science.abi4708 (PMC8501941; doi:10.1126/science.abi4708)
Supplement: 20210622-1 [file science.abi4708.v1.pdf]

Cite as: T. A. Tummino *et al.*, *Science* 10.1126/science.abi4708 (2021).

Drug-induced phospholipidosis confounds drug repurposing for SARS-CoV-2

Tia A. Tummino[1,2,3,4]†, Veronica V. Rezelj[5]†, Benoit Fischer[6]†, Audrey Fischer[6]†, Matthew J. O'Meara[7], Blandine Monel[8], Thomas Vallet[5], Kris M. White[9,10], Ziyang Zhang[3,4,11,12], Assaf Alon[13], Heiko Schadt[6], Henry R. O'Donnell[1], Jiankun Lyu[1,3,4], Romel Rosales[9,10], Briana L. McGovern[9,10], Raveen Rathnasinghe[9,10,14], Sonia Jangra[9,10], Michael Schotsaert[9,10], Jean-René Galarneau[15], Nevan J. Krogan[3,4,11,16], Laszlo Urban[15], Kevan M. Shokat[3,4,11,12], Andrew C. Kruse[13], Adolfo García-Sastre[9,10,17,18], Olivier Schwartz[8], Francesca Moretti[6]*, Marco Vignuzzi[5]*, Francois Pognan[6]*, Brian K. Shoichet[1,3,4]*

[1]Department of Pharmaceutical Chemistry, University of California San Francisco (UCSF), San Francisco, CA, USA. [2]Graduate Program in Pharmaceutical Sciences and Pharmacogenomics, UCSF, San Francisco, CA, USA. [3]Quantitative Biosciences Institute (QBI), UCSF, San Francisco, CA, USA. [4]QBI COVID-19 Research Group (QCRG), San Francisco, CA, USA. [5]Institut Pasteur, Viral Populations and Pathogenesis Unit, CNRS UMR 3569, 75724 Paris, Cedex 15, France. [6]Novartis Institutes for BioMedical Research, Preclinical Safety, Basel, Switzerland. [7]Department of Computational Medicine and Bioinformatics, University of Michigan, Ann Arbor, MI, USA. [8]Institut Pasteur, Virus and Immunity Unit, CNRS UMR 3569, 75724 Paris, Cedex 15, France. [9]Department of Microbiology, Icahn School of Medicine at Mount Sinai, New York, NY, USA. [10]Global Health and Emerging Pathogens Institute, Icahn School of Medicine at Mount Sinai, New York, NY, USA. [11]Department of Cellular and Molecular Pharmacology, UCSF, San Francisco, CA, USA. [12]Howard Hughes Medical Institute, UCSF, San Francisco, CA, USA. [13]Department of Biological Chemistry and Molecular Pharmacology, Blavatnik Institute, Harvard Medical School, Boston, MA, USA. [14]Graduate School of Biomedical Sciences, Icahn School of Medicine at Mount Sinai, New York, NY, USA. [15]Novartis Institutes for BioMedical Research, Preclinical Safety, Cambridge, MA, USA. [16]Gladstone Institute of Data Science and Biotechnology, J. David Gladstone Institutes, San Francisco, CA, USA. [17]Department of Medicine, Division of Infectious Diseases, Icahn School of Medicine at Mount Sinai, New York, NY, USA. [18]Tisch Cancer Institute, Icahn School of Medicine at Mount Sinai, New York, NY, USA.

†These authors contributed equally to this work.

***Corresponding author. Email: bshoichet@gmail.com (B.K.S.); francois.pognan@novartis.com (F.P.); marco.vignuzzi@pasteur.fr (M.V.); francesca.moretti@novartis.com (F.M.)**

Repurposing drugs as treatments for COVID-19 has drawn much attention. Beginning with sigma receptor ligands, and expanding to other drugs from screening in the field, we became concerned that phospholipidosis was a shared mechanism underlying the antiviral activity of many repurposed drugs. For all of the 23 cationic amphiphilic drugs tested, including hydroxychloroquine, azithromycin, amiodarone, and four others already in clinical trials, phospholipidosis was monotonically correlated with antiviral efficacy. Conversely, drugs active against the same targets that did not induce phospholipidosis were not antiviral. Phospholipidosis depends on the physicochemical properties of drugs, and does not reflect specific target-based activities, rather it may be considered a toxic confound in early drug discovery. Early detection of phospholipidosis could eliminate these artifacts, enabling a focus on molecules with therapeutic potential.

The outbreak of COVID-19 has inspired multiple drug repurposing screens to find antiviral therapeutics that can be rapidly brought to the clinic (*1*). To date, over 1,974 drugs and investigational drugs have reported to have in vitro activity against SARS-CoV-2 (*1*) (Fig. 1). Since almost all of these act against human targets, and might be unlikely to be viable against a novel virus (*2*), the question of mechanism of action arises.

Our interest in this question was motivated by the discovery that human sigma receptors were candidates for modulating SARS-CoV-2 infection (*3*), and that drugs and reagents like chloroquine, haloperidol, clemastine, and PB28—all with nanomolar affinity against one or both sigma receptors—had cellular antiviral IC_{50} values in the 300 nM to 5 μM range. Subsequently, we investigated over 50 different molecules with a wide range of affinities at these receptors. While this found molecules with relatively potent antiviral activity, there was little correlation between receptor potency and antiviral efficacy in cells (figs. S1 to S3 and table S1). Whereas drugs like amiodarone, sertraline, and tamoxifen had mid-to high-nM antiviral IC_{50}s, other potent sigma ligands, such as melperone and DTG, were without measurable antiviral activity. Intriguingly, the antiviral sigma drugs were all cationic at physiological pH and relatively hydrophobic, while those that were inactive against the virus were often smaller and more polar. This cationic-amphiphilic character was shared by many of the hits emerging from other phenotypic screens (Fig. 1 and fig. S4), suggesting it was this physico-chemical property that might explain cellular antiviral activity, instead of a specific on-target activity (*4*).

If the cationic-amphiphilic nature of these molecules led to antiviral activity in vitro, rather than their target-based

activities, one would expect this physical property to reflect a shared cellular mechanism. Indeed, cationic amphiphilic drugs (CADs) can provoke phospholipidosis in cells and organs (*5*). This side effect is characterized by the formation of vesicle-like structures and "foamy" or "whorled" membranes (*5*, *6*), and is thought to arise by CAD disruption of lipid homeostasis. CADs accumulate in intracellular compartments such as endosomes and lysosomes where they can directly or indirectly inhibit lipid processing (*5*). Modulation of these same lipid processing pathways is critical for viral replication (*7*), and inhibiting phospholipid production has previously been associated with inhibition of coronavirus replication (*8*). CADs have in vitro activity against multiple viruses including Severe Acute Respiratory Syndrome, Middle East Respiratory Syndrome, Ebola, Zika, Dengue, and filoviruses (*9*), though CAD-induction of phospholipidosis has only been proposed as an antiviral mechanism for Marburg virus (*10*). Finally, among the most potent known phospholipidosis inducers are amiodarone (*11*) and chloroquine (*12*, *13*), which are also potent inhibitors of SARS-CoV-2 replication in vitro (*14*–*16*), while drugs from SARS-CoV-2 phenotypic screens, such as chlorpromazine (*17*) and tamoxifen (*16*), are also known to induce phospholipidosis (*18*). As an effect that rarely occurs at concentrations lower than 100 nM, that does not appear to translate from in vitro to in vivo antiviral activity, and that can result in dose-limiting toxicity (*19*), phospholipidosis may be a confound to true antiviral drug discovery.

Here, we investigate the association between phospholipidosis and antiviral activity against SARS-CoV-2 in cell culture. This apparently general mechanism may be responsible for many of the drug repurposing hits for SARS-CoV-2, and an extraordinary amount of effort and resources lavished on drug discovery against this disease. We explore the prevalence of this confound in SARS-CoV-2 repurposing studies, how phospholipidosis correlates with inhibition of viral infection, and how to eliminate such hits rapidly so as to focus on drugs with genuine potential against COVID-19, and against new pandemics yet to arise.

Results

Correlation of phospholipidosis and antiviral activity

To investigate the role of phospholipidosis in antiviral activity in vitro, we tested 19 drugs for their induction of this effect in A549 cells using the well-established NBD-PE staining assay (*20*). Here, the vesicular lipidic bodies characteristic of the effect may be quantified by high content imaging (Fig. 2A). Three classes of drugs and reagents were initially investigated: A. Sigma-binding antiviral CADs we had discovered, like amiodarone, sertraline, chlorpromazine, and clemastine (nine total); these molecules are predicted or known to induce phospholipidosis; B. Analogs of these CADs that no longer bound sigma receptors, but were still antiviral (four total); these molecules are predicted to induce phospholipidosis despite their lack of sigma binding; and C. Sigma-binding, non-antiviral drugs, like melperone and DTG, that are more polar than classic CADs (two total); these molecules are predicted not to induce phospholipidosis. Of the nine sigma-binding CADs that were antiviral (class A), six of which were also reported in literature phenotypic screens as inhibitors of COVID-19, eight induced phospholipidosis, consistent with the hypothesis (Fig. 2, A and B, and figs. S5 and S6). The only non-phospholipidosis inducing antiviral from this set was elacridar, a promiscuous P-glycoprotein inhibitor; this drug may therefore be active via another mechanism. Intriguingly, analogs of the potent sigma ligand PB28 that had lost their sigma-binding activity but remained CADs (ZZY-10-051 and ZZY-10-061; Fig. 2, B to F, and figs. S5 to S8), did induce phospholipidosis, as did the antipsychotic olanzapine and the antihistamine diphenhydramine, which are weak sigma receptor ligands but are structurally related to potent sigma receptor ligands like chlorpromazine and clemastine. Finally, melperone and DTG, which are potent cationic sigma receptor ligands but are not antiviral, did not induce phospholipidosis (Fig. 2, A and B, and figs. S5 and S6; class C). These results do not prove phospholipidosis as the antiviral mechanism but are consistent with the phospholipidosis hypothesis.

If phospholipidosis is responsible for antiviral activity, then other molecules known to induce phospholipidosis should be antiviral. We tested three CADs for antiviral activity, including ebastine, ellipticine, and Bix01294, all of which are reported to induce phospholipidosis (*21*) (Bix01294 and ebastine have also been reported as drug repurposing hits against SARS-CoV-2 (*22*)). We further tested azithromycin, also reported to induce phospholipidosis (*23*), but having different physical properties from typical CADs. We first confirmed phospholipidosis-inducing activity for these molecules (Fig. 2B and figs. S5 and S6). All four molecules were next shown to be antiviral, as ever in this work using live virus assays (e.g., SARS-CoV-2 strain BetaCoV/France/IDF0372/2020; Methods), with IC_{50} values in the 400 nM to 3 μM range, overlapping with the activities of other CADs we and others have identified for SARS-CoV-2 (*22*) (fig. S6). This too was consistent with the antiviral phospholipidosis hypothesis.

For phospholipidosis to explain antiviral activity, we might expect a correlation between concentration-response curves for phospholipidosis and for antiviral activity. We compared concentrations that induce phospholipidosis to those that inhibit SARS-CoV-2 for each drug individually. Most correlations were high—not only did antiviral activity occur in the same concentration ranges as phospholipidosis, but R^2 values, ranging from 0.51 to 0.94, supported a quantitative relationship between the two effects (Fig. 3A). We then

fit a sigmoidal model through all the 107 phospholipidosis versus antiviral activity observations (comprised of six concentration measurements each for 16 phospholipidosis-inducing drugs) and observed a strong negative correlation (R^2 = 0.65, 95%CI [.52, 0.76]) between induced phospholipidosis and SARS-CoV-2 viral load across all observations for all 16 drugs. Because phospholipidosis and antiviral effects are both saturable, the sigmoidal curve-fit plateaus at the extremes (Fig. 3B).

Concurrent measurement of viral infection and drug induced phospholipidosis

In the previous experiments, drug-induced phospholipidosis and drug antiviral activity were measured separately. To measure the two effects in the same cells at the same time, we dosed cells with either 1 or 10 μM of five characteristic CADs (amiodarone, sertraline, PB28, hydroxychloroquine (HCQ), and Bix01294), followed by a mock or SARS-CoV-2 infection, and quantified phospholipidosis and the accumulation of viral spike protein (Fig. 4A and fig. S9). Compared to DMSO, drug treatments led to substantial increases in NBD-PE aggregates, indicating increased phospholipidosis (fig. S9). At 1 μM drug concentrations, SARS-CoV-2 spike protein was readily stained, and one could visualize both spike protein and phospholipidosis in the same cells (yellow puncta), suggesting at this low concentration of drug—often close to the antiviral IC_{50} value—both phospholipidosis and viral infection co-occur, though even here viral staining was reduced relative to the DMSO treated controls. As drug concentration rose to 10 μM, viral spike protein staining dropped while staining for phospholipidosis increased (fig. S9); there was nearly complete loss of spike protein signal with a concomitant increase in phospholipidosis (Fig. 4A) for all treatments. In seven-point concentration-response curves for amiodarone, sertraline, and PB28, viral staining monotonically decreased as phospholipidosis increased (Fig. 4, B and C).

CADs are common among drug repurposing hits for SARS-CoV-2 and other viruses

With the strong correlation between CAD phospholipidosis and antiviral efficacy (Fig. 3), including drugs that have been found in multiple SARS-CoV-2 repurposing studies, we investigated the prevalence of phospholipidosis-inducing CADs among 1,974 repurposing hits identified in the literature. We focused on 12 studies, including two screens of the ReFRAME library (*24*, *25*), screens of the NCATS “approved drug” and “bioactive” libraries (*15*), among others (*3*, *14*, *16*, *22*, *26*–*30*). Together, these 12 screens found 310 drugs, investigational drugs, and reagents that were antiviral in vitro against SARS-CoV-2. We used two physico-chemical features to identify likely CADs: drugs with calculated Log octanol:water coefficients above 3 (cLogP ≥ 3), and with pK_a values ≥ 7.4 (*31*, *32*). We then further filtered for drugs that topologically resembled known phospholipidosis inducers (*18*, *21*) using an ECFP4-based Tanimoto coefficient ((Tc) ≥ 0.4) (table S2). Sixty percent of the 310 drugs passed the cLogP and pK_a threshold; 34% also resembled a known phospholipidosis inducer (Fig. 1 and figs. S4 and S10).

Although the two physical property filters do not capture atypical phospholipidosis inducers such as azithromycin, they do capture 16 of the other 18 CADs we had already tested (missing only the medium phospholipidosis inducers olanzapine and ellipticine); intriguingly, nine of these, including amiodarone, sertraline, chlorpromazine, Bix01294, clemastine, and benztropine also appeared in at least one of the 12 other repurposing studies. To probe the reliability of this association, we tested another five drugs that passed our filters, and had been reported as antiviral against SARS-CoV-2, for their induction of phospholipidosis. Not only were all five active in the NBD-PE assay, but we confirmed SARS-CoV-2 antiviral activity for these drugs (fig. S10). Additionally, these molecules fit into the sigmoidal model relating phospholipidosis to viral load (salmon points overlaid with sigmoidal model; Fig. 3B). Finally, we note a preliminary identification of 30 CADs, 19 of which overlap with the literature-derived SARS-CoV-2 list, active against other viruses including Middle East Respiratory Syndrome and Severe Acute Respiratory Syndrome (*33*), Ebola (*34*–*36*), Marburg (*36*, *37*), Hepatitis C (*38*), and Dengue (*39*) (table S3). It may be that most drugs repurposed against many viruses are CADs whose antiviral activities can be attributed to phospholipidosis.

Animal efficacy for repurposed drugs

Though phospholipidosis is considered a drug-induced side effect, it remains possible that it can be leveraged for antiviral efficacy. Accordingly, we tested four of the repurposed, phospholipidosis-inducing drugs most potent against SARS-CoV-2 in vitro, amiodarone, sertraline, PB28 and tamoxifen (*5*, *18*), for efficacy in a murine model of COVID-19 (*40*). In the same model, we also tested elacridar, which does not induce phospholipidosis (Fig. 2B) and remdesivir, which is unlikely to induce phospholipidosis at concentrations relevant to its antiviral activity. All molecules had relatively long half-lives, especially in the lung where tissue C_{max} values often exceeded 10 μM after a 10 mg/kg dose, or 10 to 1000 times higher than their in vitro antiviral IC_{50}, suggesting that exposure would be high enough for efficacy (tables S4 to S8). Guided by the pharmacokinetics of each drug, mice were dosed either once (amiodarone and elacridar) or twice per day (remdesivir, PB28, tamoxifen, and sertraline), for three days. Two hours following the first dose, mice were intranasally infected with 1×10^4 PFU of SARS-CoV-2 and lung viral titers were measured after a three-day infection period. Notwithstanding their high lung exposure, the four phospholipidosis-inducing

drugs had little effect on viral propagation in the mice. Conversely, remdesivir reduced viral load by two to three orders of magnitude. While the cationic non-phospholipidosis drug elacridar had a modest antiviral effect, it did not rise to statistical significance (Fig. 5) and mice given elacridar doses higher than 3 mg/kg exhibited toxicities that limited further study.

Because phospholipidosis is typically an in vivo side effect that appears after chronic dosing, we then pre-treated mice with five-fold higher concentrations (50 mg/kg) of amiodarone over twelve days prior to a 3-day infection period. Even here, no diminution of viral titer was observed in mouse lungs after infection, and amiodarone offered no protection from infection-induced weight loss or from pulmonary inflammation and epithelial necrosis, as measured by histopathology (Fig. 5 and fig. S11). We note that foamy vacuolation and whorled vacuoles that are the hallmarks of phospholipidosis were not seen in lung and spleen by light or transmission electron microscopy. It is thus possible that this treatment was not long enough to induce protective phospholipidosis. Still, the in vitro activities of the phospholipidosis-inducing drugs did not readily translate in vivo, and drugs whose antiviral activity arises due to phospholipidosis seem non-viable for clinical progression.

Discussion

The emergence of COVID-19 has motivated intense effort to repurpose drugs as SARS-CoV-2 antivirals. An extraordinary number of diverse, apparently unrelated hits have emerged (*1*). A key observation from this work is that many, perhaps most of these are active in antiviral assays via induction of phospholipidosis (Fig. 1 and figs. S4 and S10). This disrupts lysosomal lipid catabolism and trafficking, which may in turn disrupt the double membrane vesicles that the virus creates and on which it depends for propagation. Quantitatively, there is a close in vitro correlation between drug-induced phospholipidosis and antiviral activity, both drug-by-drug and over the set of drugs tested here (Fig. 3). The effect is predictive: molecules that induce phospholipidosis are antiviral over the same concentration range, irrespective of whether they are cationic amphiphilic drugs (CADs) or not (e.g., azithromycin), while molecules that are related by target activity to the CADs, but are more polar and do not induce phospholipidosis (e.g., melperone and DTG), are not antiviral. Unfortunately, CAD induction of phospholipidosis, at least at the potencies observed here, does not translate in vivo (Fig. 5). More encouragingly, this study illuminates a method to rapidly identify confounds in cellular antiviral screens, allowing one to eliminate them from further study and to focus on those molecules with true potential.

Although the molecular mechanisms for the antiviral effects of phospholipidosis remain unclear, certain associations may be tentatively advanced. SARS-CoV-2, like many viruses, subverts the cell to produce double membrane vesicles in which it replicates (*41*–*43*). Disruption of lipid homeostasis by the induction of phospholipidosis may disrupt these vesicles, reducing viral replication. The disruption of lysosomal (*44*) and endosomal (*45*) compartments and CAD-induced shifts in compartmental pH (*46*) may further affect viral entry and propagation (*47*). Accordingly, targeting the endosomal-lysosomal pathway has been suggested as a viable strategy against SARS-CoV-2 infection (*48*), but developing potent and targeted inhibitors remains challenging.

The cost to the community of investments in what appears to be a confound merits consideration for future pandemics. According to the DrugBank COVID-19 dashboard (*49*), which draws from U.S. and international clinical trials, putatively antiviral CADs have been promoted into an astonishing 316 Phase I to Phase III clinical trials against COVID-19. While 57% of these study the phospholipidosis-inducing CADs hydroxychloroquine (Fig. 3A, top row) or chloroquine, that still leaves 136 trials across 33 other predicted or known phospholipidosis-inducers. Using conservative estimates (*50*, *51*), the expense of the clinical trials component alone, over the last year, for phospholipidosis-inducing CADs may be over $6 billion US dollars (table S9).

Certain caveats merit airing. First, the correlation between antiviral activity and phospholipidosis, as strong as it is, does not illuminate the mechanism by which phospholipidosis is antiviral. Phospholipidosis is itself only partly understood, and there are no good genetic or chemical ways to either inhibit its induction by drugs nor to promote it by target-selective reagents. Second, predicting whether a molecule will induce phospholipidosis remains challenging, and even non-CAD molecules can induce it. Thus, we have chosen conservative criteria to predict phospholipidosis-inducers, which may miss many drugs. Third, phospholipidosis is a confound that only affects drugs repurposed for direct antiviral activity—it is irrelevant for drugs like dexamethasone (*52*) and fluvoxamine (*53*) that have been repurposed for immunomodulation in COVID-19, nor is it relevant for CADs whose antiviral activity is well-below the concentration range where phospholipidosis occurs. Fourth, our estimates of the clinical trial costs of phospholipidosis-inducing CADs are obviously rough. Finally, we do not exclude exploiting phospholipidosis therapeutically, though we suspect that would have to proceed through a more target-directed mechanism than that of the CADs studied here.

These caveats should not obscure the central observation of this study. Many drugs repurposed for antiviral activity against SARS-CoV-2 are cationic amphiphiles, and despite their diverse structures and multiple targets, many likely have their antiviral effects via a single shared mechanism: phospholipidosis. Both because of the side effects with which

it is associated, and the limited efficacy to which it leads—rarely better than 100 nM in vitro—drugs active due to phospholipidosis are unlikely to translate in vivo (Fig. 5). Many resources will be saved by counter-screening for phospholipidosis in simple cellular assays (*20*), allowing investigators to focus on drugs with genuine promise as antivirals.

REFERENCES AND NOTES

1. M. V. Kuleshov, D. J. Stein, D. J. B. Clarke, E. Kropiwnicki, K. M. Jagodnik, A. Bartal, J. E. Evangelista, J. Hom, M. Cheng, A. Bailey, A. Zhou, L. B. Ferguson, A. Lachmann, A. Ma'ayan, The COVID-19 Drug and Gene Set Library. *Patterns* **1**, 100090 (2020). doi:10.1016/j.patter.2020.100090 Medline
2. A. Edwards, What are the odds of finding a COVID-19 drug from a lab repurposing screen? *J. Chem. Inf. Model.* **60**, 5727–5729 (2020). doi:10.1021/acs.jcim.0c00861 Medline
3. D. E. Gordon, G. M. Jang, M. Bouhaddou, J. Xu, K. Obernier, K. M. White, M. J. O'Meara, V. V. Rezelj, J. Z. Guo, D. L. Swaney, T. A. Tummino, R. Hüttenhain, R. M. Kaake, A. L. Richards, B. Tutuncuoglu, H. Foussard, J. Batra, K. Haas, M. Modak, M. Kim, P. Haas, B. J. Polacco, H. Braberg, J. M. Fabius, M. Eckhardt, M. Soucheray, M. J. Bennett, M. Cakir, M. J. McGregor, Q. Li, B. Meyer, F. Roesch, T. Vallet, A. Mac Kain, L. Miorin, E. Moreno, Z. Z. C. Naing, Y. Zhou, S. Peng, Y. Shi, Z. Zhang, W. Shen, I. T. Kirby, J. E. Melnyk, J. S. Chorba, K. Lou, S. A. Dai, I. Barrio-Hernandez, D. Memon, C. Hernandez-Armenta, J. Lyu, C. J. P. Mathy, T. Perica, K. B. Pilla, S. J. Ganesan, D. J. Saltzberg, R. Rakesh, X. Liu, S. B. Rosenthal, L. Calviello, S. Venkataramanan, J. Liboy-Lugo, Y. Lin, X.-P. Huang, Y. Liu, S. A. Wankowicz, M. Bohn, M. Safari, F. S. Ugur, C. Koh, N. S. Savar, Q. D. Tran, D. Shengjuler, S. J. Fletcher, M. C. O'Neal, Y. Cai, J. C. J. Chang, D. J. Broadhurst, S. Klippsten, P. P. Sharp, N. A. Wenzell, D. Kuzuoglu-Ozturk, H.-Y. Wang, R. Trenker, J. M. Young, D. A. Cavero, J. Hiatt, T. L. Roth, U. Rathore, A. Subramanian, J. Noack, M. Hubert, R. M. Stroud, A. D. Frankel, O. S. Rosenberg, K. A. Verba, D. A. Agard, M. Ott, M. Emerman, N. Jura, M. von Zastrow, E. Verdin, A. Ashworth, O. Schwartz, C. d'Enfert, S. Mukherjee, M. Jacobson, H. S. Malik, D. G. Fujimori, T. Ideker, C. S. Craik, S. N. Floor, J. S. Fraser, J. D. Gross, A. Sali, B. L. Roth, D. Ruggero, J. Taunton, T. Kortemme, P. Beltrao, M. Vignuzzi, A. García-Sastre, K. M. Shokat, B. K. Shoichet, N. J. Krogan, A SARS-CoV-2 protein interaction map reveals targets for drug repurposing. *Nature* **583**, 459–468 (2020). doi:10.1038/s41586-020-2286-9 Medline
4. J. L. Dahlin, D. S. Auld, I. Rothenaigner, S. Haney, J. Z. Sexton, J. W. M. Nissink, J. Walsh, J. A. Lee, J. M. Strelow, F. S. Willard, L. Ferrins, J. B. Baell, M. A. Walters, B. K. Hua, K. Hadian, B. K. Wagner, Nuisance compounds in cellular assays. *Cell Chem. Biol.* **28**, 356–370 (2021). doi:10.1016/j.chembiol.2021.01.021 Medline
5. B. Breiden, K. Sandhoff, Emerging mechanisms of drug-induced phospholipidosis. *Biol. Chem.* **401**, 31–46 (2019). doi:10.1515/hsz-2019-0270 Medline
6. J. A. Shayman, A. Abe, Drug induced phospholipidosis: An acquired lysosomal storage disorder. *Biochim. Biophys. Acta* **1831**, 602–611 (2013). doi:10.1016/j.bbalip.2012.08.013 Medline
7. M. Abu-Farha, T. A. Thanaraj, M. G. Qaddoumi, A. Hashem, J. Abubaker, F. Al-Mulla, The role of lipid metabolism in COVID-19 virus infection and as a drug target. *Int. J. Mol. Sci.* **21**, 3544 (2020). doi:10.3390/ijms21103544 Medline
8. C. Müller, M. Hardt, D. Schwudke, B. W. Neuman, S. Pleschka, J. Ziebuhr, Inhibition of cytosolic phospholipase $A_{2\alpha}$ impairs an early step of coronavirus replication in cell culture. *J. Virol.* **92**, 01463-17 (2018). doi:10.1128/JVI.01463-17 Medline
9. C. Salata, A. Calistri, C. Parolin, A. Baritussio, G. Palù, Antiviral activity of cationic amphiphilic drugs. *Expert Rev. Anti-Infect. Ther.* **15**, 483–492 (2017). doi:10.1080/14787210.2017.1305888 Medline
10. A. P. Gunesch, F. J. Zapatero-Belinchón, L. Pinkert, E. Steinmann, M. P. Manns, G. Schneider, T. Pietschmann, M. Brönstrup, T. von Hahn, Filovirus antiviral activity of cationic amphiphilic drugs is associated with lipophilicity and ability to induce phospholipidosis. *Antimicrob. Agents Chemother.* **64**, e00143-20 (2020). doi:10.1128/AAC.00143-20 Medline
11. B. Guigui, S. Perrot, J. P. Berry, J. Fleury-Feith, N. Martin, J. M. Métreau, D. Dhumeaux, E. S. Zafrani, Amiodarone-induced hepatic phospholipidosis: A morphological alteration independent of pseudoalcoholic liver disease. *Hepatology* **8**, 1063–1068 (1988). doi:10.1002/hep.1840080514 Medline
12. J. Müller-Höcker, H. Schmid, M. Weiss, U. Dendorfer, G. S. Braun, Chloroquine-induced phospholipidosis of the kidney mimicking Fabry's disease: Case report and review of the literature. *Hum. Pathol.* **34**, 285–289 (2003). doi:10.1053/hupa.2003.36 Medline
13. O. G. Fitzhugh, A. A. Nelson, O. L. Holland, The chronic oral toxicity of chloroquine. *J. Pharmacol. Exp. Ther.* **93**, 147–152 (1948). Medline
14. D. E. Gordon, J. Hiatt, M. Bouhaddou, V. V. Rezelj, S. Ulferts, H. Braberg, A. S. Jureka, K. Obernier, J. Z. Guo, J. Batra, R. M. Kaake, A. R. Weckstein, T. W. Owens, M. Gupta, S. Pourmal, E. W. Titus, M. Cakir, M. Soucheray, M. McGregor, Z. Cakir, G. Jang, M. J. O'Meara, T. A. Tummino, Z. Zhang, H. Foussard, A. Rojc, Y. Zhou, D. Kuchenov, R. Hüttenhain, J. Xu, M. Eckhardt, D. L. Swaney, J. M. Fabius, M. Ummadi, B. Tutuncuoglu, U. Rathore, M. Modak, P. Haas, K. M. Haas, Z. Z. C. Naing, E. H. Pulido, Y. Shi, I. Barrio-Hernandez, D. Memon, E. Petsalaki, A. Dunham, M. C. Marrero, D. Burke, C. Koh, T. Vallet, J. A. Silvas, C. M. Azumaya, C. Billesbølle, A. F. Brilot, M. G. Campbell, A. Diallo, M. S. Dickinson, D. Diwanji, N. Herrera, N. Hoppe, H. T. Kratochvil, Y. Liu, G. E. Merz, M. Moritz, H. C. Nguyen, C. Nowotny, C. Puchades, A. N. Rizo, U. Schulze-Gahmen, A. M. Smith, M. Sun, I. D. Young, J. Zhao, D. Asarnow, J. Biel, A. Bowen, J. R. Braxton, J. Chen, C. M. Chio, U. S. Chio, I. Deshpande, L. Doan, B. Faust, S. Flores, M. Jin, K. Kim, V. L. Lam, F. Li, J. Li, Y.-L. Li, Y. Li, X. Liu, M. Lo, K. E. Lopez, A. A. Melo, F. R. Moss 3rd, P. Nguyen, J. Paulino, K. I. Pawar, J. K. Peters, T. H. Pospiech Jr., M. Safari, S. Sangwan, K. Schaefer, P. V. Thomas, A. C. Thwin, R. Trenker, E. Tse, T. K. M. Tsui, F. Wang, N. Whitis, Z. Yu, K. Zhang, Y. Zhang, F. Zhou, D. Saltzberg, QCRG Structural Biology Consortium, A. J. Hodder, A. S. Shun-Shion, D. M. Williams, K. M. White, R. Rosales, T. Kehrer, L. Miorin, E. Moreno, A. H. Patel, S. Rihn, M. M. Khalid, A. Vallejo-Gracia, P. Fozouni, C. R. Simoneau, T. L. Roth, D. Wu, M. A. Karim, M. Ghoussaini, I. Dunham, F. Berardi, S. Weigang, M. Chazal, J. Park, J. Logue, M. McGrath, S. Weston, R. Haupt, C. J. Hastie, M. Elliott, F. Brown, K. A. Burness, E. Reid, M. Dorward, C. Johnson, S. G. Wilkinson, A. Geyer, D. M. Giesel, C. Baillie, S. Raggett, H. Leech, R. Toth, N. Goodman, K. C. Keough, A. L. Lind, Zoonomia Consortium, R. J. Klesh, K. R. Hemphill, J. Carlson-Stevermer, J. Oki, K. Holden, T. Maures, K. S. Pollard, A. Sali, D. A. Agard, Y. Cheng, J. S. Fraser, A. Frost, N. Jura, T. Kortemme, A. Manglik, D. R. Southworth, R. M. Stroud, D. R. Alessi, P. Davies, M. B. Frieman, T. Ideker, C. Abate, N. Jouvenet, G. Kochs, B. Shoichet, M. Ott, M. Palmarini, K. M. Shokat, A. García-Sastre, J. A. Rassen, R. Grosse, O. S. Rosenberg, K. A. Verba, C. F. Basler, M. Vignuzzi, A. A. Peden, P. Beltrao, N. J. Krogan, Comparative host-coronavirus protein interaction networks reveal pan-viral disease mechanisms. *Science* **370**, eabe9403 (2020). doi:10.1126/science.abe9403 Medline
15. C. Z. Chen, P. Shinn, Z. Itkin, R. T. Eastman, R. Bostwick, L. Rasmussen, R. Huang, M. Shen, X. Hu, K. M. Wilson, B. M. Brooks, H. Guo, T. Zhao, C. Klump-Thomas, A. Simeonov, S. G. Michael, D. C. Lo, M. D. Hall, W. Zheng, Drug repurposing screen for compounds inhibiting the cytopathic effect of SARS-CoV-2. *Front. Pharmacol.* **11**, 592737 (2021). doi:10.3389/fphar.2020.592737 Medline
16. S. Weston, C. M. Coleman, R. Haupt, J. Logue, K. Matthews, Y. Li, H. M. Reyes, S. R. Weiss, M. B. Frieman, Broad anti-coronavirus activity of food and drug administration-approved drugs against SARS-CoV-2 in vitro and SARS-CoV in vivo. *J. Virol.* **94**, e01218–e01220 (2020). doi:10.1128/JVI.01218-20 Medline
17. M. Plaze, D. Attali, M. Prot, A.-C. Petit, M. Blatzer, F. Vinckier, L. Levillayer, J. Chiaravalli, F. Perin-Dureau, A. Cachia, G. Friedlander, F. Chrétien, E. Simon-Loriere, R. Gaillard, Inhibition of the replication of SARS-CoV-2 in human cells by the FDA-approved drug chlorpromazine. *Int. J. Antimicrob. Agents* **57**, 106274 (2021). doi:10.1016/j.ijantimicag.2020.106274 Medline
18. M. Muehlbacher, P. Tripal, F. Roas, J. Kornhuber, Identification of drugs inducing phospholipidosis by novel in vitro data. *ChemMedChem* **7**, 1925–1934 (2012). doi:10.1002/cmdc.201200306 Medline
19. M. Obeidat, A. L. Isaacson, S. J. Chen, M. Ivanovic, D. Holanda, Zebra-like bodies in COVID-19: Is phospholipidosis evidence of hydroxychloroquine induced acute kidney injury? *Ultrastruct. Pathol.* **44**, 519–523 (2020). doi:10.1080/01913123.2020.1850966 Medline
20. J. K. Morelli, M. Buehrle, F. Pognan, L. R. Barone, W. Fieles, P. J. Ciaccio, Validation of an in vitro screen for phospholipidosis using a high-content biology platform. *Cell Biol. Toxicol.* **22**, 15–27 (2006). doi:10.1007/s10565-006-0176-z Medline
21. S. A. Shahane, R. Huang, D. Gerhold, U. Baxa, C. P. Austin, M. Xia, Detection of phospholipidosis induction: A cell-based assay in high-throughput and high-

content format. *J. Biomol. Screen.* **19**, 66–76 (2014). doi:10.1177/1087057113502851 Medline
22. M. Dittmar, J. S. Lee, K. Whig, E. Segrist, M. Li, K. Jurado, K. Samby, H. Ramage, D. Schultz, S. Cherry, Drug repurposing screens reveal FDA approved drugs active against SARS-Cov-2. bioRxiv 2020.06.19.161042 [Preprint] (2020). https://doi.org/10.1101/2020.06.19.161042.
23. E. T. Baronas, J.-W. Lee, C. Alden, F. Y. Hsieh, Biomarkers to monitor drug-induced phospholipidosis. *Toxicol. Appl. Pharmacol.* **218**, 72–78 (2007). doi:10.1016/j.taap.2006.10.015 Medline
24. L. Riva, S. Yuan, X. Yin, L. Martin-Sancho, N. Matsunaga, L. Pache, S. Burgstaller-Muehlbacher, P. D. De Jesus, P. Teriete, M. V. Hull, M. W. Chang, J. F.-W. Chan, J. Cao, V. K.-M. Poon, K. M. Herbert, K. Cheng, T. H. Nguyen, A. Rubanov, Y. Pu, C. Nguyen, A. Choi, R. Rathnasinghe, M. Schotsaert, L. Miorin, M. Dejosez, T. P. Zwaka, K.-Y. Sit, L. Martinez-Sobrido, W.-C. Liu, K. M. White, M. E. Chapman, E. K. Lendy, R. J. Glynne, R. Albrecht, E. Ruppin, A. D. Mesecar, J. R. Johnson, C. Benner, R. Sun, P. G. Schultz, A. I. Su, A. García-Sastre, A. K. Chatterjee, K.-Y. Yuen, S. K. Chanda, Discovery of SARS-CoV-2 antiviral drugs through large-scale compound repurposing. *Nature* **586**, 113–119 (2020). doi:10.1038/s41586-020-2577-1 Medline
25. M. A. Bakowski, N. Beutler, E. Chen, T.-T. H. Nguyen, M. G. Kirkpatrick, M. Parren, L. Yang, J. Ricketts, A. K. Gupta, M. V. Hull, P. G. Schultz, D. R. Burton, A. K. Chatterjee, C. W. McNamara, T. F. Rogers, Oral drug repositioning candidates and synergistic remdesivir combinations for the prophylaxis and treatment of COVID-19. bioRxiv 2020.06.16.153403 [Preprint] (2020). https://doi.org/10.1101/2020.06.16.153403.
26. N. Drayman, K. A. Jones, S.-A. Azizi, H. M. Froggatt, K. Tan, N. I. Maltseva, S. Chen, V. Nicolaescu, S. Dvorkin, K. Furlong, R. S. Kathayat, M. R. Firpo, V. Mastrodomenico, E. A. Bruce, M. M. Schmidt, R. Jedrzejczak, M. Á. Muñoz-Alía, B. Schuster, V. Nair, J. W. Botten, C. B. Brooke, S. C. Baker, B. C. Mounce, N. S. Heaton, B. C. Dickinson, A. Jaochimiak, G. Randall, S. Tay, Drug repurposing screen identifies masitinib as a 3CLpro inhibitor that blocks replication of SARS-CoV-2 in vitro. bioRxiv 2020.08.31.274639 [Preprint] (2020). https://doi.org/10.1101/2020.08.31.274639.
27. C. Mirabelli, J. W. Wotring, C. J. Zhang, S. M. McCarty, R. Fursmidt, T. Frum, N. S. Kadambi, A. T. Amin, T. R. O'Meara, C. D. Pretto, J. R. Spence, J. Huang, K. D. Alysandratos, D. N. Kotton, S. K. Handelman, C. E. Wobus, K. J. Weatherwax, G. A. Mashour, M. J. O'Meara, J. Z. Sexton, Morphological Cell Profiling of SARS-CoV-2 Infection Identifies Drug Repurposing Candidates for COVID-19. bioRxiv 2020.05.27.117184 [Preprint] (2020). https://doi.org/10.1101/2020.05.27.117184.
28. F. Touret, M. Gilles, K. Barral, A. Nougairède, J. van Helden, E. Decroly, X. de Lamballerie, B. Coutard, In vitro screening of a FDA approved chemical library reveals potential inhibitors of SARS-CoV-2 replication. *Sci. Rep.* **10**, 13093 (2020). doi:10.1038/s41598-020-70143-6 Medline
29. X. Xiao, C. Wang, D. Chang, Y. Wang, X. Dong, T. Jiao, Z. Zhao, L. Ren, C. S. Dela Cruz, L. Sharma, X. Lei, J. Wang, Identification of potent and safe antiviral therapeutic candidates against SARS-CoV-2. *Front. Immunol.* **11**, 586572 (2020). doi:10.3389/fimmu.2020.586572 Medline
30. S. Jeon, M. Ko, J. Lee, I. Choi, S. Y. Byun, S. Park, D. Shum, S. Kim, Identification of antiviral drug candidates against SARS-CoV-2 from FDA-approved drugs. *Antimicrob. Agents Chemother.* **64**, e00819–e00820 (2020). doi:10.1128/AAC.00819-20 Medline
31. R. Lowe, R. C. Glen, J. B. O. Mitchell, Predicting phospholipidosis using machine learning. *Mol. Pharm.* **7**, 1708–1714 (2010). doi:10.1021/mp100103e Medline
32. J.-P. H. T. M. Ploemen, J. Kelder, T. Hafmans, H. van de Sandt, J. A. van Burgsteden, P. J. M. Saleminki, E. van Esch, Use of physicochemical calculation of pKa and CLogP to predict phospholipidosis-inducing potential: A case study with structurally related piperazines. *Exp. Toxicol. Pathol.* **55**, 347–355 (2004). doi:10.1078/0940-2993-00338 Medline
33. J. Dyall, C. M. Coleman, B. J. Hart, T. Venkataraman, M. R. Holbrook, J. Kindrachuk, R. F. Johnson, G. G. Olinger Jr., P. B. Jahrling, M. Laidlaw, L. M. Johansen, C. M. Lear-Rooney, P. J. Glass, L. E. Hensley, M. B. Frieman, Repurposing of clinically developed drugs for treatment of Middle East respiratory syndrome coronavirus infection. *Antimicrob. Agents Chemother.* **58**, 4885–4893 (2014). doi:10.1128/AAC.03036-14 Medline
34. P. B. Madrid, R. G. Panchal, T. K. Warren, A. C. Shurtleff, A. N. Endsley, C. E. Green, A. Kolokoltsov, R. Davey, I. D. Manger, L. Gilfillan, S. Bavari, M. J. Tanga, Evaluation of Ebola virus inhibitors for drug repurposing. *ACS Infect. Dis.* **1**, 317–326 (2015). doi:10.1021/acsinfecdis.5b00030 Medline
35. L. M. Johansen, L. E. DeWald, C. J. Shoemaker, B. G. Hoffstrom, C. M. Lear-Rooney, A. Stossel, E. Nelson, S. E. Delos, J. A. Simmons, J. M. Grenier, L. T. Pierce, H. Pajouhesh, J. Lehár, L. E. Hensley, P. J. Glass, J. M. White, G. G. Olinger, A screen of approved drugs and molecular probes identifies therapeutics with anti–Ebola virus activity. *Sci. Transl. Med.* **7**, 290ra89 (2015). doi:10.1126/scitranslmed.aaa5597 Medline
36. H. Cheng, C. M. Lear-Rooney, L. Johansen, E. Varhegyi, Z. W. Chen, G. G. Olinger, L. Rong, Inhibition of Ebola and Marburg virus entry by G protein-coupled receptor antagonists. *J. Virol.* **89**, 9932–9938 (2015). doi:10.1128/JVI.01337-15 Medline
37. G. Gehring, K. Rohrmann, N. Atenchong, E. Mittler, S. Becker, F. Dahlmann, S. Pöhlmann, F. W. R. Vondran, S. David, M. P. Manns, S. Ciesek, T. von Hahn, The clinically approved drugs amiodarone, dronedarone and verapamil inhibit filovirus cell entry. *J. Antimicrob. Chemother.* **69**, 2123–2131 (2014). doi:10.1093/jac/dku091 Medline
38. S. He, B. Lin, V. Chu, Z. Hu, X. Hu, J. Xiao, A. Q. Wang, C. J. Schweitzer, Q. Li, M. Imamura, N. Hiraga, N. Southall, M. Ferrer, W. Zheng, K. Chayama, J. J. Marugan, T. J. Liang, Repurposing of the antihistamine chlorcyclizine and related compounds for treatment of hepatitis C virus infection. *Sci. Transl. Med.* **7**, 282ra49 (2015). doi:10.1126/scitranslmed.3010286 Medline
39. M. K. Poh, G. Shui, X. Xie, P.-Y. Shi, M. R. Wenk, F. Gu, U18666A, an intra-cellular cholesterol transport inhibitor, inhibits dengue virus entry and replication. *Antiviral Res.* **93**, 191–198 (2012). doi:10.1016/j.antiviral.2011.11.014 Medline
40. K. M. White, R. Rosales, S. Yildiz, T. Kehrer, L. Miorin, E. Moreno, S. Jangra, M. B. Uccellini, R. Rathnasinghe, L. Coughlan, C. Martinez-Romero, J. Batra, A. Rojc, M. Bouhaddou, J. M. Fabius, K. Obernier, M. Dejosez, M. J. Guillén, A. Losada, P. Avilés, M. Schotsaert, T. Zwaka, M. Vignuzzi, K. M. Shokat, N. J. Krogan, A. García-Sastre, Plitidepsin has potent preclinical efficacy against SARS-CoV-2 by targeting the host protein eEF1A. *Science* **371**, 926–931 (2021). doi:10.1126/science.abf4058 Medline
41. G. Wolff, C. E. Melia, E. J. Snijder, M. Bárcena, Double-membrane vesicles as platforms for viral replication. *Trends Microbiol.* **28**, 1022–1033 (2020). doi:10.1016/j.tim.2020.05.009 Medline
42. P. V'kovski, A. Kratzel, S. Steiner, H. Stalder, V. Thiel, Coronavirus biology and replication: Implications for SARS-CoV-2. *Nat. Rev. Microbiol.* **19**, 155–170 (2021). doi:10.1038/s41579-020-00468-6 Medline
43. G. Wolff, R. W. A. L. Limpens, J. C. Zevenhoven-Dobbe, U. Laugks, S. Zheng, A. W. M. de Jong, R. I. Koning, D. A. Agard, K. Grünewald, A. J. Koster, E. J. Snijder, M. Bárcena, A molecular pore spans the double membrane of the coronavirus replication organelle. *Science* **369**, 1395–1398 (2020). doi:10.1126/science.abd3629 Medline
44. L. K. K. Holland, I. Ø. Nielsen, K. Maeda, M. Jäättelä, SnapShot: Lysosomal Functions. *Cell* **181**, 748–748.e1 (2020). doi:10.1016/j.cell.2020.03.043 Medline
45. O. O. Glebov, Understanding SARS-CoV-2 endocytosis for COVID-19 drug repurposing. *FEBS J.* **287**, 3664–3671 (2020). doi:10.1111/febs.15369 Medline
46. R. Hamaguchi, J. Haginaka, T. Tanimoto, Y. Kuroda, Maintenance of luminal pH and protease activity in lysosomes/late endosomes by vacuolar ATPase in chlorpromazine-treated RAW264 cells accumulating phospholipids. *Cell Biol. Toxicol.* **30**, 67–77 (2014). doi:10.1007/s10565-014-9269-2 Medline
47. U. Norinder, A. Tuck, K. Norgren, V. Munic Kos, Existing highly accumulating lysosomotropic drugs with potential for repurposing to target COVID-19. *Biomed. Pharmacother.* **130**, 110582 (2020). doi:10.1016/j.biopha.2020.110582 Medline
48. N. Yang, H.-M. Shen, Targeting the endocytic pathway and autophagy process as a novel therapeutic strategy in COVID-19. *Int. J. Biol. Sci.* **16**, 1724–1731 (2020). doi:10.7150/ijbs.45498 Medline
49. D. S. Wishart, C. Knox, A. C. Guo, S. Shrivastava, M. Hassanali, P. Stothard, Z. Chang, J. Woolsey, DrugBank: A comprehensive resource for in silico drug discovery and exploration. *Nucleic Acids Res.* **34**, D668–D672 (2006). doi:10.1093/nar/gkj067 Medline
50. H. H. Wong, A. Jessup, A. Sertkaya, A. Birkenbach, A. Berlind, J. Eyraud, "Examination of clinical trial costs and barriers for drug development"

(HHSP23337007T, Eastern Research Group, Inc., 2014); https://aspe.hhs.gov/system/files/pdf/77166/rpt_erg.pdf.
51. A. Sertkaya, H.-H. Wong, A. Jessup, T. Beleche, Key cost drivers of pharmaceutical clinical trials in the United States. *Clin. Trials* **13**, 117–126 (2016). doi:10.1177/1740774515625964 Medline
52. RECOVERY Collaborative Group, Dexamethasone in hospitalized patients with Covid-19. *N. Engl. J. Med.* **384**, 693–704 (2021). doi:10.1056/NEJMoa2021436 Medline
53. E. J. Lenze, C. Mattar, C. F. Zorumski, A. Stevens, J. Schweiger, G. E. Nicol, J. P. Miller, L. Yang, M. Yingling, M. S. Avidan, A. M. Reiersen, Fluvoxamine vs placebo and clinical deterioration in outpatients with symptomatic COVID-19: A randomized clinical trial. *JAMA* **324**, 2292–2300 (2020). doi:10.1001/jama.2020.22760 Medline
54. L. M. Wingler, C. McMahon, D. P. Staus, R. J. Lefkowitz, A. C. Kruse, Distinctive activation mechanism for angiotensin receptor revealed by a synthetic nanobody. *Cell* **176**, 479–490.e12 (2019). doi:10.1016/j.cell.2018.12.006 Medline
55. J. Buchrieser, J. Dufloo, M. Hubert, B. Monel, D. Planas, M. M. Rajah, C. Planchais, F. Porrot, F. Guivel-Benhassine, S. Van der Werf, N. Casartelli, H. Mouquet, T. Bruel, O. Schwartz, Syncytia formation by SARS-CoV-2-infected cells. *EMBO J.* **39**, e106267 (2020). doi:10.15252/embj.2020106267 Medline
56. F. Amanat, K. M. White, L. Miorin, S. Strohmeier, M. McMahon, P. Meade, W. C. Liu, R. A. Albrecht, V. Simon, L. Martinez-Sobrido, T. Moran, A. García-Sastre, F. Krammer, An in vitro microneutralization assay for SARS-CoV-2 serology and drug screening. *Curr. Protoc. Microbiol.* **58**, e108 (2020). doi:10.1002/cpmc.108 Medline
57. D. Planas, T. Bruel, L. Grzelak, F. Guivel-Benhassine, I. Staropoli, F. Porrot, C. Planchais, J. Buchrieser, M. M. Rajah, E. Bishop, M. Albert, F. Donati, M. Prot, S. Behillil, V. Enouf, M. Maquart, M. Smati-Lafarge, E. Varon, F. Schortgen, L. Yahyaoui, M. Gonzalez, J. De Sèze, H. Péré, D. Veyer, A. Sève, E. Simon-Lorière, S. Fafi-Kremer, K. Stefic, H. Mouquet, L. Hocqueloux, S. van der Werf, T. Prazuck, O. Schwartz, Sensitivity of infectious SARS-CoV-2 B.1.1.7 and B.1.351 variants to neutralizing antibodies. *Nat. Med.* **27**, 917–924 (2021). doi:10.1038/s41591-021-01318-5 Medline
58. F. Berardi, C. Abate, S. Ferorelli, V. Uricchio, N. A. Colabufo, M. Niso, R. Perrone, Exploring the importance of piperazine N-atoms for σ_2 receptor affinity and activity in a series of analogs of 1-cyclohexyl-4-[3-(5-methoxy-1,2,3,4-tetrahydronaphthalen-1-yl)propyl]piperazine (PB28). *J. Med. Chem.* **52**, 7817–7828 (2009). doi:10.1021/jm9007505 Medline
59. P.-C. Bürkner, brms: An R package for Bayesian multilevel models using Stan. *J. Stat. Softw.* **80**, 1–28 (2017). doi:10.18637/jss.v080.i01
60. A. Vehtari, A. Gelman, J. Gabry, Practical Bayesian model evaluation using leave-one-out cross-validation and WAIC. *Stat. Comput.* **27**, 1413–1432 (2017). doi:10.1007/s11222-016-9696-4
61. S. Kim, J. Chen, T. Cheng, A. Gindulyte, J. He, S. He, Q. Li, B. A. Shoemaker, P. A. Thiessen, B. Yu, L. Zaslavsky, J. Zhang, E. E. Bolton, PubChem in 2021: New data content and improved web interfaces. *Nucleic Acids Res.* **49**, D1388–D1395 (2021). doi:10.1093/nar/gkaa971 Medline
62. R. Rathnasinghe, S. Strohmeier, F. Amanat, V. L. Gillespie, F. Krammer, A. García-Sastre, L. Coughlan, M. Schotsaert, M. B. Uccellini, Comparison of transgenic and adenovirus hACE2 mouse models for SARS-CoV-2 infection. *Emerg. Microbes Infect.* **9**, 2433–2445 (2020). doi:10.1080/22221751.2020.1838955 Medline
63. L. Coughlan, A. C. Bradshaw, A. L. Parker, H. Robinson, K. White, J. Custers, J. Goudsmit, N. Van Roijen, D. H. Barouch, S. A. Nicklin, A. H. Baker, Ad5:Ad48 hexon hypervariable region substitutions lead to toxicity and increased inflammatory responses following intravenous delivery. *Mol. Ther.* **20**, 2268–2281 (2012). doi:10.1038/mt.2012.162 Medline
64. L. Coughlan, S. Vallath, A. Saha, M. Flak, I. A. McNeish, G. Vassaux, J. F. Marshall, I. R. Hart, G. J. Thomas, In vivo retargeting of adenovirus type 5 to $\alpha v\beta 6$ integrin results in reduced hepatotoxicity and improved tumor uptake following systemic delivery. *J. Virol.* **83**, 6416–6428 (2009). doi:10.1128/JVI.00445-09 Medline
65. R. Rathnasinghe, S. Jangra, A. Cupic, C. Martínez-Romero, L. C. F. Mulder, T. Kehrer, S. Yildiz, A. Choi, I. Mena, J. De Vrieze, S. Aslam, D. Stadlbauer, D. A. Meekins, C. D. McDowell, V. Balaraman, J. A. Richt, B. G. De Geest, L. Miorin, PVI study group, F. Krammer, V. Simon, A. García-Sastre, M. Schotsaert, The N501Y mutation in SARS-CoV-2 spike leads to morbidity in obese and aged mice and is neutralized by convalescent and post-vaccination human sera. medRxiv 2021.01.19.21249592 [Preprint] (2021). https://doi.org/10.1101/2021.01.19.21249592.
66. D. S. Himmelstein, User-friendly extensions of the DrugBank database v1.0, version v1.0, Zenodo (2016); https://zenodo.org/record/45579.

ACKNOWLEDGMENTS

We gratefully acknowledge the Région Ile-de-France (program DIM1Health) for the use of the Institut Pasteur imaging facility. We thank N. Aulner for assistance with image requisition and A. Danckaert for assistance with image analysis at Institut Pasteur, Paris. We thank R. Albrecht for support with the BSL3 facility and procedures at the Icahn School of Medicine at Mount Sinai, New York. We thank C. Hayden for the work with the transmission electron microscopy at Novartis. We thank T.R. O'Meara for reading the manuscript. We thank K. Obernier, M. Bouhaddou, and J.M. Fabius for contributions to the overall COVID-19 effort at QCRG. **Funding:** Supported by grants from the Defense Advanced Research Projects Agency (HR0011-19-2-0020 to B.K.S., N.J.K., A.G.-S., and K.M.S.); NIGMS R35GM122481 (to B.K.S.); National Institutes of Health (P50AI150476, U19AI135990, U19AI135972, R01AI143292, R01AI120694, P01AI063302, and R01AI122747 to N.J.K.); Excellence in Research Award (ERA) from the Laboratory for Genomics Research (LGR to N.J.K.); a collaboration between UCSF, UCB, and GSK (no. 133122P to N.J.K.); a Fast Grant for COVID-19 from the Emergent Ventures program at the Mercatus Center of George Mason University (to N.J.K.); funding from the Roddenberry Foundation (to N.J.K.); funding from F. Hoffmann-La Roche and Vir Biotechnology (to N.J.K.); gifts from QCRG philanthropic donors (to N.J.K.); funding from Institut Pasteur (to O.S. and M.V.); Urgence COVID-19 Fundraising Campaign of Institut Pasteur (to O.S. and M.V.); Labex IBEID (ANR-10-LABX-62-IBEID, to O.S. and M.V.); ANR/FRM Flash Covid PROTEO-SARS-CoV-2 (to O.S.); IDISCOVR (to O.S.); National Institutes of Health (R01GM119185 to A.C.K.); partly supported by the Center for Research for Influenza Pathogenesis, a Center of Excellence for Influenza Research and Surveillance supported by the National Institute of Allergy and Infectious Diseases (contract number HHSN272201400008C to A.G.-S.); a supplement to NIAID grant U19AI135972 and to DoD grant W81XWH-20-1-0270 (to A.G.-S.); a Fast Grant from the Mercatus Center (to A.G.-S.); the generous support of the JPB Foundation and the Open Philanthropy Project (research grant 2020-215611 (5384) to A.G.-S.); anonymous donors (to AG-S); and Z.Z. is a Damon Runyon fellow supported by the Damon Runyon Cancer Research Foundation (DRG-2281-17). **Author contributions:** The following authors designed and conceptualized the study: T.A.T, V.V.R, M.J.O., Z.Z., K.M.S., F.M., M.V., F.P., and B.K.S. The following authors performed experiments or data acquisition: T.A.T, V.V.R., B.F., A.F., B.M., T.V., K.M.W., Z.Z., A.A., H.R.O., R.R., B.L.M., R.R., S.J., M.S., and J.-R.G. The following authors conducted formal data analysis: T.A.T, V.V.R, B.F., A.F., M.J.O., B.M., T.V., K.M.W., Z.Z., A.A., H.S., J.L., and F.M. The following authors supervised or managed research: M.J.O., K.M.W., N.J.K., L.U., K.M.S., A.C.K., A.G.-S., O.S., F.M., M.V., F.P., and B.K.S. The following authors drafted the original manuscript: T.A.T., V.V.R., M.J.O, B.M., K.M.W., Z.Z., M.V., F.P., and B.K.S. All authors reviewed the manuscript prior to submission. **Competing interests:** B.K.S. is a founder of Epiodyne Ltd. and of Blue Dolphin LLC, is on the SAB of Schrodinger LLC, and his lab receives funding from Pfizer and from Apple Tree Ventures. A.C.K. is a founder and advisor to Tectonic Therapeutic, Inc., and the Institute for Protein Innovation. K.M.S. has consulting agreements for the following companies, which involve monetary and/or stock compensation: Black Diamond Therapeutics, BridGene Biosciences, Denali Therapeutics, Dice Molecules, eFFECTOR Therapeutics, Erasca, Genentech/Roche, Janssen Pharmaceuticals, Kumquat Biosciences, Kura Oncology, Merck, Mitokinin, Petra Pharma, Revolution Medicines, Type6 Therapeutics, Venthera, Wellspring Biosciences. The N.J.K. laboratory has received research support from Vir Biotechnology and F. Hoffmann-La Roche. N.J.K. has received research support from Vir Biotechnology and F. Hoffmann-La Roche. N.J.K. has consulting agreements with the Icahn School of Medicine at Mount Sinai, New York, Maze Therapeutics and Interline Therapeutics, is a shareholder of Tenaya Therapeutics and has received stocks from Maze Therapeutics and Interline Therapeutics. The A.G.-S. laboratory has received research support from Pfizer, Senhwa Biosciences, Kenall Manufacturing,

Avimex, Johnson & Johnson, Dynavax, 7Hills Pharma, Pharmamar, ImmunityBio, Accurius, Merck and Nanocomposix. and A.G.-S. has consulting agreements for the following companies involving cash and/or stock: Vivaldi Biosciences, Contrafect, 7Hills Pharma, Avimex, Vaxalto, Pagoda, Accurius, Esperovax, Farmak and Pfizer. A.G.-S. is inventor on patents and patent application on the use of antivirals for the treatment of virus infections, owned by the Icahn School of Medicine at Mount Sinai, New York. **Data and materials availability:** All data are available in the manuscript or the supplementary materials. Further information and requests for resources and reagents should be directed to B.K.S. (bshoichet@gmail.com), F. P. (francois.pognan@novartis.com), M.V (marco.vignuzzi@pasteur.fr), and F.M. (francesca.moretti@novartis.com). This work is licensed under a Creative Commons Attribution 4.0 International (CC BY 4.0) license, which permits unrestricted use, distribution, and reproduction in any medium, provided the original work is properly cited. To view a copy of this license, visit https://creativecommons.org/licenses/by/4.0/. This license does not apply to figures/photos/artwork or other content included in the article that is credited to a third party; obtain authorization from the rights holder before using such material.

SUPPLEMENTARY MATERIALS

science.sciencemag.org/cgi/content/full/science.abi4708/DC1
Materials and Methods
Figs. S1 to S11
Tables S1 to S9
References (*54*–*66*)
MDAR Reproducibility Checklist

11 March 2021; accepted 15 June 2021
Published online 22 June 2021
10.1126/science.abi4708

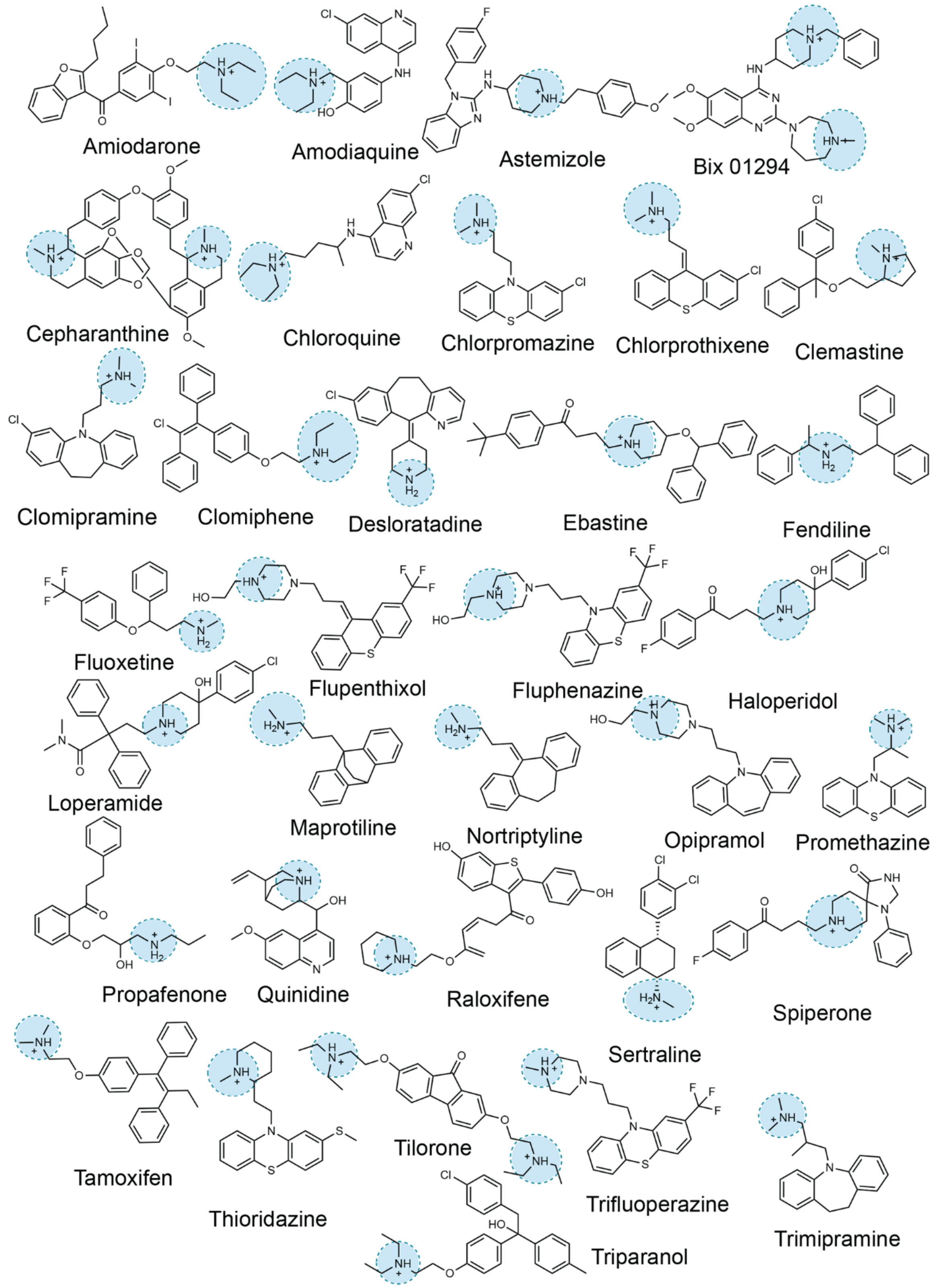

Fig. 1. Representative examples of cationic amphiphilic drugs that are identified in SARS-CoV-2 drug repurposing screens.

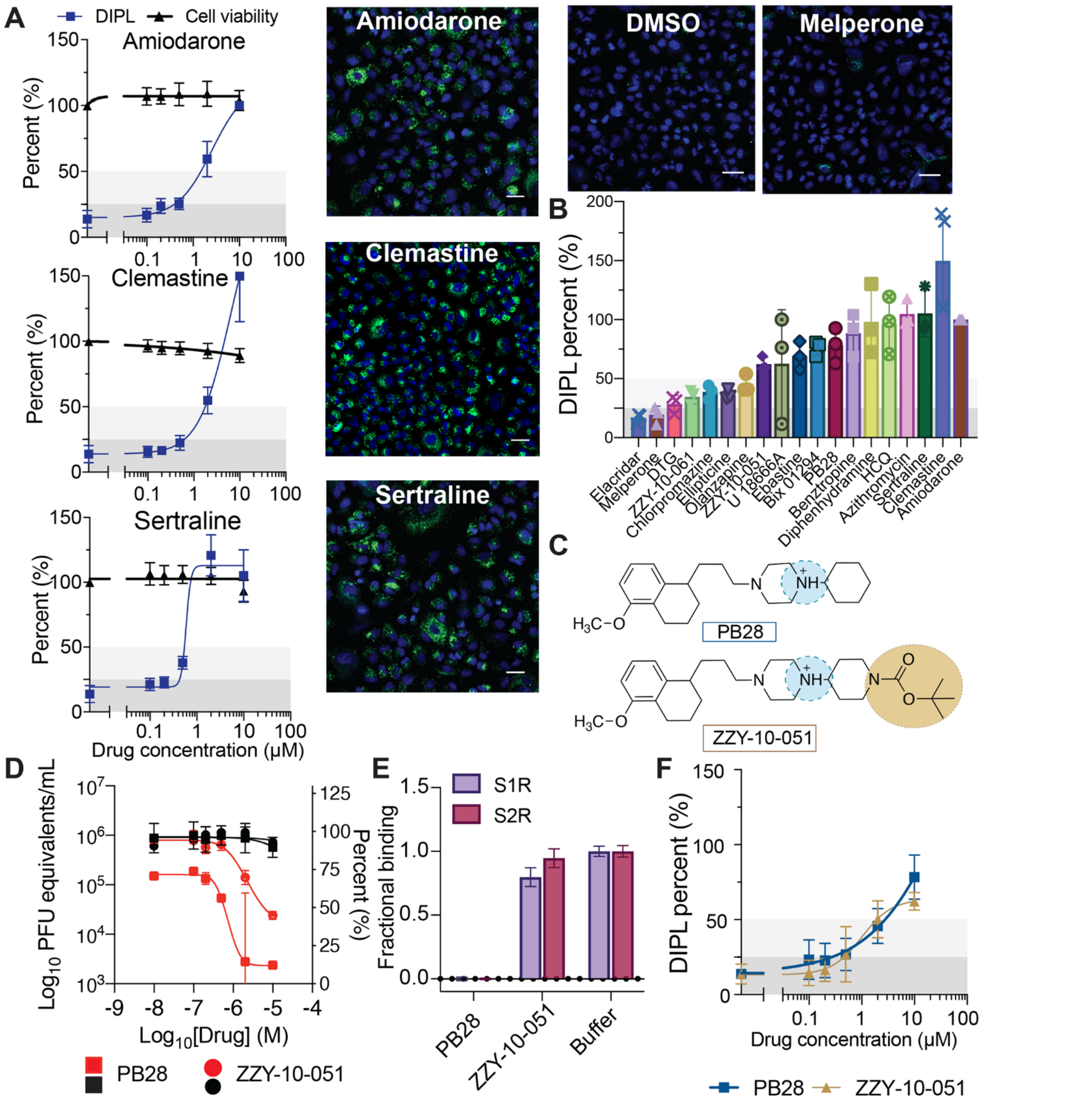
A
DIPL
Cell viability
Amiodarone
Clemastine
Sertraline
Percent (%)
Drug concentration (µM)
DMSO
Melperone
B
DIPL percent (%)
Elacridar
Melperone
DTG
ZZY-10-061
Chlorpromazine
Ellipticine
Olanzapine
ZZY-10-051
U 18666A
Ebastine
Bix 01294
PB28
Benztropine
Diphenhydramine
HCQ
Azithromycin
Sertraline
Clemastine
Amiodarone
C
PB28
ZZY-10-051
D
Log10 PFU equivalents/mL
Log10[Drug] (M)
Percent (%)
PB28
ZZY-10-051
E
S1R
S2R
Fractional binding
PB28
ZZY-10-051
Buffer
F
DIPL percent (%)
Drug concentration (µM)
PB28
ZZY-10-051

Fig. 2. Cellular phospholipidosis may confound antiviral screening results. A. Examples of NBD-PE quantification of phospholipidosis in A549 cells including dose response curves. Blue = Hoechst nuclei staining, Green = NBD-PE phospholipid staining, Red = EthD-2 staining for dead cells. Scale bars = 20 μm. Amiodarone is the positive control for assay normalization; sertraline and clemastine are two examples of high phospholipidosis inducing drugs (phospholipidosis (DIPL) > 50% of amiodarone). Images of DMSO and a non-phospholipidosis inducing molecule (melperone) are included for reference. Thresholds for determining phospholipidosis power are shaded in dark grey (low phospholipidosis), light gray (medium phospholipidosis) and no shading (high phospholipidosis). **B.** Pooled DIPL amounts (mean ± SD) at the highest non-toxic concentration tested for each drug. Results were pooled from three biological and three technical replicates and were normalized to amiodarone (100%) from the control wells in the same experimental batches. **C.** Structures of PB28 and its analog ZZY-10-051, the latter of which is inactive on the sigma receptors. **D.** Viral infectivity (red) and viability (black) data for PB28 (square) and ZZY-10-051 (circle) in A549-ACE2 cells. Data shown are mean ± SD from three technical replicates. **E.** Fractional binding of PB28 and ZZY-10-051 against Sigma-1 (purple; S1R) and Sigma-2 (maroon; S2R) normalized to a buffer control at 1.0 in a radioligand binding experiment. Data shown are mean ± SEM from three technical replicates. PB28 is a strong ligand of both Sigma-1 and Sigma-2 and has high displacement of the radioligands, whereas ZZY-10-051 is unable to displace the radioligands to a high degree at 1 μM. **F.** Dose response curves for PB28 (blue) and ZZY-10-051 (gold) show that these closely related analogs both induce phospholipidosis.

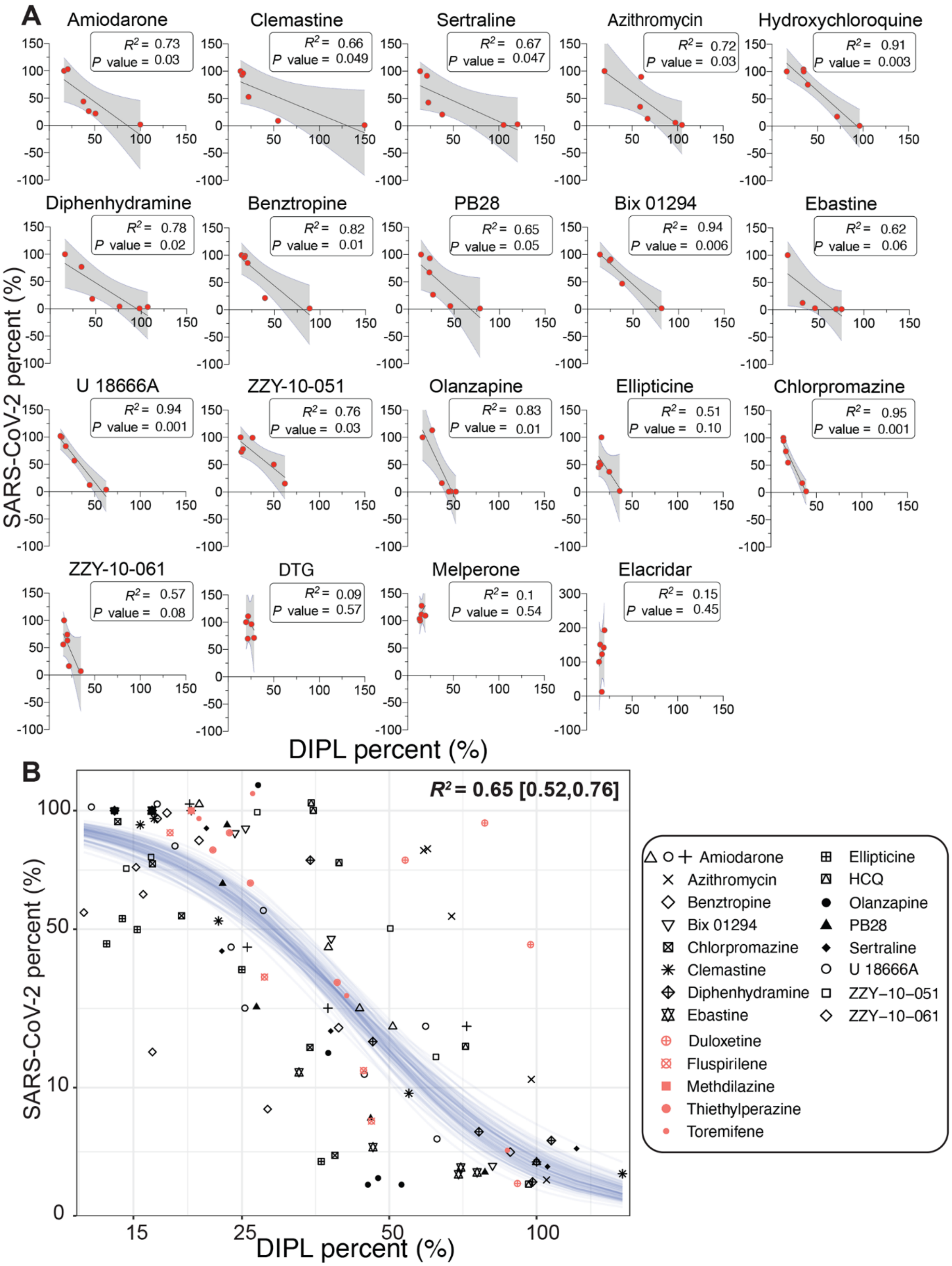
A
Amiodarone
R^2 = 0.73
P value = 0.03
Clemastine
R^2 = 0.66
P value = 0.049
Sertraline
R^2 = 0.67
P value = 0.047
Azithromycin
R^2 = 0.72
P value = 0.03
Hydroxychloroquine
R^2 = 0.91
P value = 0.003
Diphenhydramine
R^2 = 0.78
P value = 0.02
Benztropine
R^2 = 0.82
P value = 0.01
PB28
R^2 = 0.65
P value = 0.05
Bix 01294
R^2 = 0.94
P value = 0.006
Ebastine
R^2 = 0.62
P value = 0.06
U 18666A
R^2 = 0.94
P value = 0.001
ZZY-10-051
R^2 = 0.76
P value = 0.03
Olanzapine
R^2 = 0.83
P value = 0.01
Ellipticine
R^2 = 0.51
P value = 0.10
Chlorpromazine
R^2 = 0.95
P value = 0.001
ZZY-10-061
R^2 = 0.57
P value = 0.08
DTG
R^2 = 0.09
P value = 0.57
Melperone
R^2 = 0.1
P value = 0.54
Elacridar
R^2 = 0.15
P value = 0.45
SARS-CoV-2 percent (%)
DIPL percent (%)
B
R^2 = 0.65 [0.52,0.76]
SARS-CoV-2 percent (%)
DIPL percent (%)
Amiodarone
Azithromycin
Benztropine
Bix 01294
Chlorpromazine
Clemastine
Diphenhydramine
Ebastine
Duloxetine
Fluspirilene
Methdilazine
Thiethylperazine
Toremifene
Ellipticine
HCQ
Olanzapine
PB28
Sertraline
U 18666A
ZZY–10–051
ZZY–10–061

Fig. 3. Quantitative relationship between phospholipidosis and viral amounts. A. Correlations between phospholipidosis (DIPL), normalized to amiodarone at 100%, and percent of SARS-CoV-2, normalized to DMSO at 100%, in the RT-qPCR assay in A549-ACE2 cells. Each dot represents the same concentration tested in both assays. A strong negative correlation emerges, with $R^2 \geq 0.65$ and $p \leq 0.05$ for all high and medium phospholipidosis-inducing drugs except ellipticine, which is confounded by its cytotoxicity in both experiments, ebastine, and ZZY-10-61. The latter two examples are marginally significant. **B.** The SARS-CoV-2 viral loads and induced phospholipidosis magnitude for each compound and dose in A are plotted as sqrt(viral_amount_mean) ~ 10*inv_logit(hill*4/10*(log(DIPL_mean)-logIC_{50}). Fitting a sigmoid Bayesian model with weakly informative priors yields parameters and 95% credible intervals of IC_{50}: 43 [38, 48]%, hill: -5.6 [-7.0, -4.5], and Sigma 2.0 [0.14, 1.78]. Forty draws from the fit model are shown as blue lines. Salmon points overlaid with the model represent predicted phospholipidosis inducers from the literature (fig. S10).

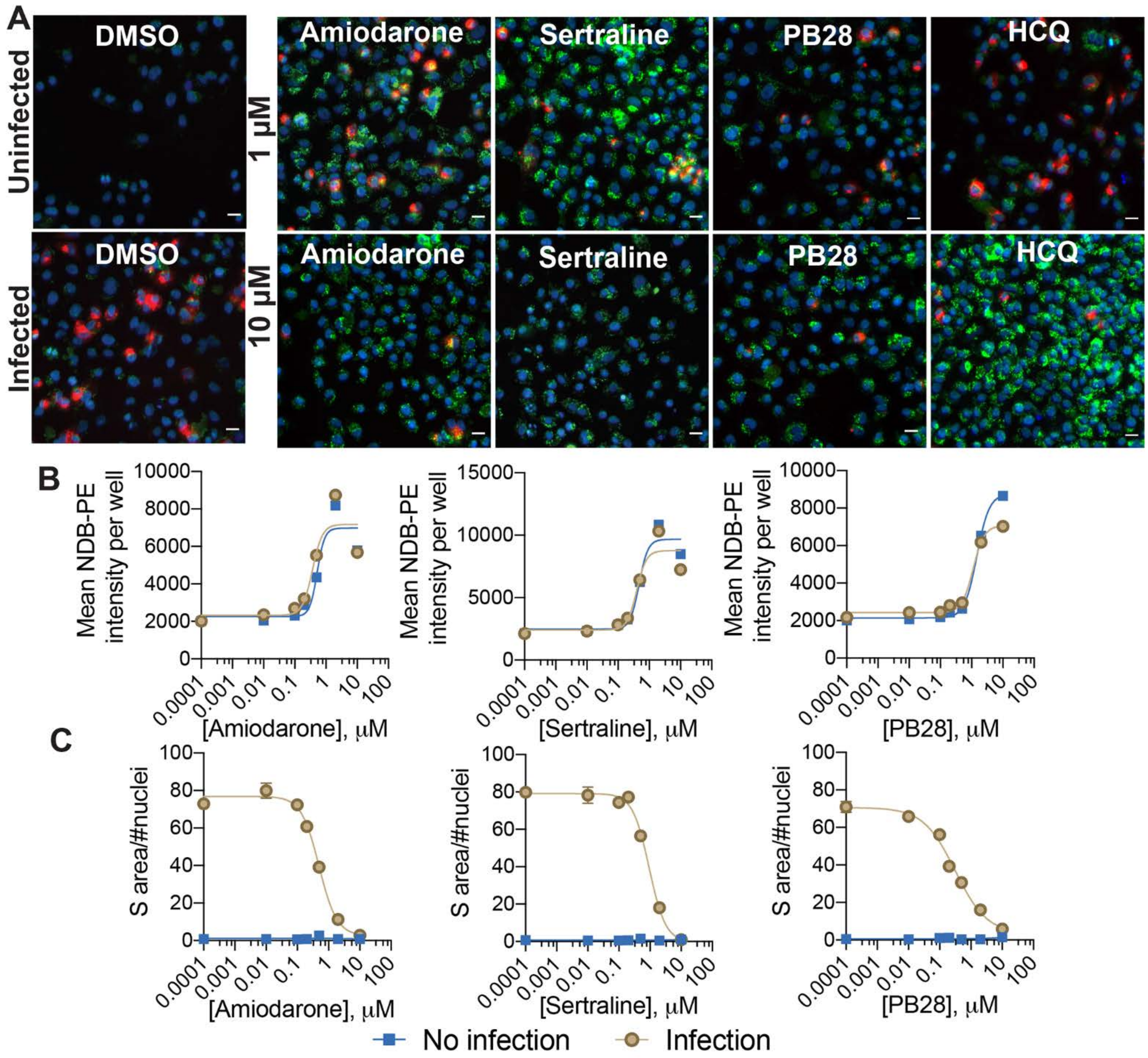

Fig. 4. Phospholipidosis and spike protein measurements in the same cellular context. A. Representative images from a co-staining experiment measuring phospholipidosis and SARS-CoV-2 spike protein in infected and uninfected A549-ACE2 cells. Five molecules (1 and 10 μM) and DMSO were measured; see fig. S9 for Bix 01294. Blue = Hoechst nuclei staining, Green = NBD-PE phospholipid staining, Red = SARS-CoV-2 spike protein staining; Yellow = coexpression of spike protein and NBD-PE. Scale bar = 20 μm. B. Concentration-response curves for phospholipidosis induction measured by NBD-PE staining in infected cells for three characteristic CADs. C. Spike protein in infected cells decreases as phospholipidosis increases. For B and C, data are mean ± SEM from four biological replicates.

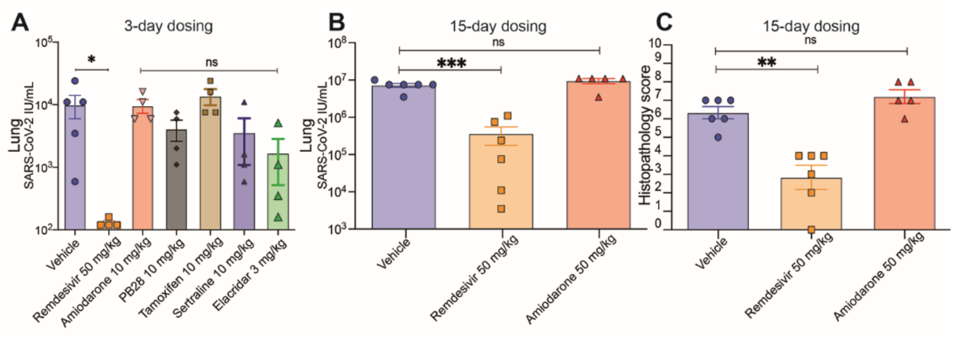

Fig. 5. Phospholipidosis-inducing drugs are not efficacious in vivo. A. Three-day dosing of six different drugs with a two-hour preincubation before SARS-CoV-2 treatment. Lung viral titers were quantified and groups were compared using the Kruskal-Wallis test ($H(7) = 22.76$, $P = 0.002$) with Dunn's multiple comparison correction indicated (vehicle $N = 5$; remdesivir $N = 4$, *$P = 0.02$). All other groups $N = 4$, ns = not significant. **B.** Fifteen-day dosing of amiodarone (50 mg/kg) compared to 3-day remdesivir dosing. Lung viral titers were quantified and groups were compared with a two-way ANOVA (main effect of treatment $F(2,9) = 19.66$, $P = 0.0005$; no main effect of mouse, $F(5,9) = 1.21$, $P = 0.38$). Individual group comparisons determined using Dunnett's multiple comparison test are indicated (vehicle $N = 6$; remdesivir $N = 6$, ***$P = 0.0008$, amiodarone $N = 5$, ns = not significant). **C.** Histopathology scores after 15-day (amiodarone) or 3-day (remdesivir) treatments as in panel B. See materials and methods for scoring breakdown. Groups were compared with a two-way ANOVA (main effect of treatment $F(2,9) = 19.05$, $P = 0.0006$; no main effect of mouse, $F(5,9) = 0.78$, $P = 0.59$). Individual group comparisons determined using Dunnett's multiple comparison test are indicated (vehicle $N = 6$; remdesivir $N = 6$, **$P = 0.0014$, amiodarone $N = 5$, ns = not significant). All data are mean ± SEM.
