# Supplementary material for: Drug-induced phospholipidosis confounds drug repurposing for SARS-CoV-2
Source: Science. Author manuscript; Available in PMC 2022 Jan 30. (PMC8501941; doi:10.1126/science.abi4708)
Supplement: Supplementary file 1 [file NIHMS1744547-supplement-Supplementary_Material.pdf]

Science AAAS

Supplementary Materials for

Drug-induced phospholipidosis confounds drug repurposing for SARS-CoV-2

Tia A. Tummino[1,2,3,4]†, Veronica V. Rezelj[5]†, Benoit Fischer[6]†, Audrey Fischer[6]†, Matthew J. O'Meara[7], Blandine Monel[8], Thomas Vallet[5], Kris M. White[9,10], Ziyang Zhang[3,4,11,12], Assaf Alon[13], Heiko Schadt[6], Henry R. O'Donnell[1], Jiankun Lyu[1,3,4], Romel Rosales[9,10], Briana L. McGovern[9,10], Raveen Rathnasinghe[9,10,14], Sonia Jangra[9,10], Michael Schotsaert[9,10], Jean-René Galarneau[15], Nevan J. Krogan[3,4,11,16], Laszlo Urban[15], Kevan M. Shokat[3,4,11,12], Andrew C. Kruse[13], Adolfo García-Sastre[9,10,17,18], Olivier Schwartz[8], Francesca Moretti*[6], Marco Vignuzzi[5]*, Francois Pognan[6]*, Brian K. Shoichet[1,3,4]*

* Corresponding authors: bshoichet@gmail.com, francois.pognan@novartis.com, marco.vignuzzi@pasteur.fr, francesca.moretti@novartis.com

This PDF file includes:

Materials and Methods
Figs. S1 – S11
Tables S3 – S9
References (*54* – *66*)

Other Supplementary Materials for this manuscript include the following:

Tables S1– S2 (.xlsx)
MDAR Reproducibility Checklist (.pdf)

Materials and Methods

Competition Binding Assays

Competition curves were measured using membranes from Expi293F cells (Thermo, A14527) with a stably integrated tetracycline repressor (*54*) (provided by the lab of Dr. Robert Lefkowitz, lefko001@receptor-biol.duke.edu) transiently overexpressing either the human Sigma-1 or the human Sigma-2 receptors. For binding assays, ^{3}H-(+)-pentazocine (PerkinElmer, net1056250uc) and ^{3}H-DTG (PerkinElmer, net986250uc) were used as the radioactive probes for Sigma-1 and Sigma-2, respectively. Membranes were incubated in a 100 µL reaction with 50 mM Tris (pH 8.0), 0.1% (w/v) bovine serum albumin (Rockland, BSA-50), 10 nM radioligand, and eight concentrations of the competing cold ligand. Reactions were incubated for two hours at 37 °C and then were terminated by filtration through a glass fiber filter using a Brandel harvester. Glass fiber filters were pre-soaked in 0.3% (v/v) polyethylenimine for 30 minutes at room temperature before harvesting. All reactions were performed in triplicate using a 96-well block format. After the membranes were transferred to the filters and washed, the filters were soaked in 5 mL Cytoscint scintillation fluid (MP Biomedicals, 0188245304) overnight, and radioactivity was measured using a Beckman Coulter LS 6500 scintillation counter. K_D values for each receptor were calculated in GraphPad Prism version 8.0.0 (San Diego, CA) using a saturation binding assay model with eight concentrations of the radioactive ligand. Non-specific binding was measured in the presence of 10 µM haloperidol (Tocris, 0931). The K_D of the sigma-1 radioligand probe was measured to be 21 nM and K_D of the sigma-2 radioligand probe was measured to be 15 nM, and these values were used to calculate K_i values for cold ligands in GraphPad Prism using the Nonlinear fit, one site-fit K_i method after normalizing to solvent baseline at 100 (Percent = 100*Value/Baseline).

Antiviral drug and cell viability assays at Institut Pasteur

A549-ACE2 cells were kindly provided by the lab of Dr Olivier Schwartz (*55*). A549-ACE2 cells were propagated at 37°C, 5% CO_2 in DMEM supplemented with 10% FBS and 20 µg/mL blasticidin S. 6250 of these cells per well were seeded into 384-well plates in DMEM (10% FBS) and incubated for 24 hours at 37°C, 5% CO_2. Two hours prior to infection, the media was replaced with 50 µL of DMEM (2% FBS) containing the compound of interest at the indicated concentration. At the time of infection, the media was replaced with virus inoculum (multiplicity of infection, MOI = 0.1 PFU/cell) and incubated for one hour at 37°C, 5% CO_2. The SARS-CoV-2 strain used

(BetaCoV/France/IDF0372/2020) was propagated once in Vero-E6 cells and is a kind gift from the National Reference Centre for Respiratory Viruses at Institut Pasteur, Paris, originally supplied through the European Virus Archive goes Global platform. Following the adsorption period, the inoculum was removed, replaced with 50 µL of drug-containing media, and cells were incubated for an additional 72 hours at 37°C, 5% CO_2. At this point, the cell culture supernatant was harvested and viral load was assessed by RT-qPCR. Briefly, the cell culture supernatant was collected, heat inactivated at 95°C for 5 minutes and used for RT-qPCR analysis. SARS-CoV-2 specific primers targeting the N gene region: 5′-TAATCAGACAAGGAACTGATTA-3′ (Forward) and 5′-CGAAGGTGTGACTTCCATG-3′ (Reverse) were used with the Luna Universal One-Step RT-qPCR Kit (New England Biolabs, #E3005) in an Applied Biosystems QuantStudio 6 thermocycler, with the following cycling conditions: 55°C for 10 min, 95°C for 1 minute, and 40 cycles of 95°C for 10 seconds, followed by 60°C for 1 minute. The number of viral genomes is expressed as PFU equivalents/mL, and was calculated by performing a standard curve with RNA derived from a viral stock with a known viral titer. Data was fit using nonlinear regression and IC_{50}s for each experiment were determined using GraphPad Prism version 8.0.0 (San Diego, CA).

Cell viability was assayed in uninfected, drug-treated cells using the CellTiter-Glo assay following the manufacturer's instructions (Promega, G7570). Luminescence was measured in a Tecan Infinity 2000 plate reader, and percentage viability calculated relative to untreated cells (100% viability) and cells lysed with 20% ethanol (0% viability), included in each experiment.

Cells and viruses for Anti-NP Immunofluorescence

Vero E6 (ATCC, CRL-1586) cells were maintained in DMEM (Corning, 10-013) supplemented with 10% FB (Peak Serum, PS-FB) and Penicillin/Streptomycin (Corning, 30-002) at 37°C and 5% CO_2. This cell line was regularly screened for mycoplasma contamination using the Universal Detection Kit (ATCC, 30-1012K). Cells were infected with SARS-CoV-2, isolate USA-WA1/2020 (BEI Resources NR-52281) under biosafety level 3 (BSL3) containment in accordance to the biosafety protocols developed by the Icahn School of Medicine at Mount Sinai. Viral stocks were grown in Vero E6 cells as previously described (*56*) and were validated by genome sequencing.

Antiviral drug and cell viability assays at Mt. Sinai

Two thousand Vero E6 cells were seeded into 96-well plates in DMEM (10% FBS) and incubated for 24 hours at 37°C, 5% CO_2. Two hours before infection, the medium was replaced with 100 µL of DMEM (2% FBS) containing the compound of interest at concentrations 50% greater than those indicated, including a DMSO control. Plates were then transferred into the BSL3 facility and 100 PFU (MOI = 0.025) was added in 50 µL of DMEM (2% FBS), bringing the final compound concentration to those indicated. Plates were then incubated for 48 hours at 37°C. After infection, supernatants were removed and cells were fixed with 4% formaldehyde for 24 hours prior to being removed from the BSL3 facility. The cells were then immunostained for the viral NP protein (an in-house mAb 1C7, provided by Dr. Thomas Moran, Thomas.Moran@mssm.edu) with a DAPI counterstain. Infected cells (488 nm) and total cells (DAPI) were quantified using the Celigo (Nexcelcom) imaging cytometer. Infectivity was measured by the accumulation of viral NP protein in the nucleus of the Vero E6 cells (fluorescence accumulation). Percent infection was quantified as ((Infected cells/Total cells) - Background) *100 and the DMSO control was then set to 100% infection for analysis. Data was fit using nonlinear regression and IC_{50}s for each experiment were determined using GraphPad Prism version 8.0.0 (San Diego, CA). Cytotoxicity was also performed using the MTT assay (Roche), according to the manufacturer's instructions. Cytotoxicity was performed in uninfected VeroE6 cells with same compound dilutions and concurrent with viral replication assay. All assays were performed in biologically independent triplicates. The Vero E6 cell line used in this study is a kidney cell line; therefore, we cannot exclude that lung cells yield different results for some inhibitors (see also 'Antiviral drug and cell viability assays at Institut Pasteur' for studies carried out at Institut Pasteur).

Phospholipidosis quantification in uninfected cells

Phospholipidosis was assessed as previously described (*20*). Briefly, A549 cells (ATCC, ref. CCL-185) were cultivated in Ham's F12-K Medium (ThermoFisher, ref. 21127-022) containing 10% FCS and seeded in a black 96-well plate with clear bottom at a density of 15000 cells per well. The day after seeding, the cells were treated for 24 hours with a dose-range of different drugs in presence of 7.5 µM NBD-PE (ThermoFisher, ref. N360). The final DMSO concentration was 0.2%. Amiodarone (Hydrochloride salt, internal Novartis supply) was used as a positive control for phospholipidosis. Before imaging, the cells were stained for 20 minutes at 37°C, 5% CO_2 with a solution containing Hoechst (ThermoFisher, ref. H3570) (10 µg/ml) and Ethidium homodimer-2 (EthD-2; ThermoFisher, E3599) (2 µM) in complete culture medium for visualizing the total and dead cell populations

respectively. Cells were washed once with pre-warmed HBSS +/+ and images were taken on an Arrayscan XTI (ThermoFisher) equipped with a 20x objective and the LED/filter combinations BGRFR_386_23, BGRFR_485_20 and BGRFR_549_15 for acquisition of Hoechst, NBD-PE and EthD-2 dyes respectively. The images were analyzed using the HCS Studio software (ThermoFisher). Briefly, the nuclei were detected using the Hoechst dye and the dead cell nuclei showing a co-staining with EthD-2 were excluded from the analysis. Then, the dots of NBD-PE were detected in the cytoplasm of each cell corresponding to a dilation of the nuclei to a maximum of 50 µm width and the total intensity of NBD-PE was measured in each cell for quantification of DIPL. After imaging, cytotoxicity was assessed doing an ATP quantification with the CellTiter-Glo® 2.0 Cell Viability assay kit (Promega, ref. G9241) following manufacturer's instructions.

NBD-PE and Spike protein staining in infected cells

15000 A549-ACE2 cells per well were seeded into 96-well plates in DMEM (10% FBS) and incubated for 24 hours at 37°C, 5% CO_2. Two hours prior to infection, the media was replaced with 100 µL of DMEM (2% FBS) containing the compound of interest at the indicated concentration and 7.5 µM NBD-PE dye (Invitrogen, N360). At the time of infection, the media was replaced with virus inoculum (MOI 2 PFU/cell) and incubated for one hour at 37°C, 5% CO_2. Following the adsorption period, the inoculum was removed, replaced with 100 µL of drug and NBD-PE-containing media, and cells incubated for an additional 24 hours at 37°C, 5% CO_2. At this point, the supernatant was replaced with DMEM containing 10 µg/mL of Hoechst (Invitrogen, H3570) and 2 µM Ethidium homodimer-2 (Invitrogen, E3599) and incubated for 20 min at 37°C, 5% CO_2. The cells were then washed once with HBSS+/+ and fixed with 4% (v/v) formalin in PBS. The plates were then imaged using the Opera Phenix High Content Screening System, taking 12 images per well with a 20x objective. Subsequently, the cells were washed and stained for Spike antigen, to identify infected cells. Briefly, the cells were permeabilized with Triton 0.1% for 10 minutes at room temperature and nonspecific staining was blocked with PBS 5% Bovine Serum Albumin (BSA) for two hours at room temperature. The cells were then stained with 1ug/mL of the anti-Spike human monoclonal antibody mAb48 recognizing the RBD (*57*) (kindly provided by the lab of Hugo Mouquet, hugo.mouquet@pasteur.fr) overnight at 4°C and with a goat anti-human secondary antibody AlexaFluor647 for one hour at room temperature. Upon staining, the cells were imaged once more in the same fields of view using the Opera Phenix screening system.

For image analysis the Columbus image analysis (PerkinElmer) system was used. Nuclei touching the border of the image were rejected. Living cells (not stained with Ethidium homodimer 2) were identified and the total intensity of NBD-PE dots in a 50 µm radius circle centered on the nucleus of living cells was measured, using the spot detection algorithm. To quantify SARS-CoV-2 infection, the area of Spike+ staining was quantified and normalized by the number of nuclei using the same software. Two-way ANOVAs were performed on pooled data from three independent experiments each in biological triplicate (**Fig. S9**) using GraphPad Prism version 8.0.0 (San Diego, CA).

General Chemical Synthesis Procedure

Anhydrous solvents were purchased from Acros Organics. Unless specified below, all chemical reagents were purchased from Sigma-Aldrich and AK Scientific. Commercial solvents and reagents were used as received. All reactions were performed in oven-dried glassware fitted with rubber septa under a positive pressure of argon, unless otherwise noted. Air- and moisture-sensitive liquids were transferred via syringe. Solutions were concentrated by rotary evaporation at or below 40 °C. Analytical thin-layer chromatography (TLC) was performed using glass plates pre-coated with silica gel (0.25-mm, 60-Å pore size, 230−400 mesh, Merck KGA) impregnated with a fluorescent indicator (254 nm). TLC plates were visualized by exposure to ultraviolet light (UV), then were stained by submersion in a 10% solution of phosphomolybdic acid (PMA) in ethanol or an acidic ethanolic solution of p-anisaldehyde followed by brief heating on a hot plate. The latter solution was prepared by sequential additions of concentrated sulfuric acid (5.0 mL), glacial acetic acid (1.5 mL) and p-anisaldehyde (3.7 mL) to absolute ethanol (135 mL) at 23 °C with efficient stirring. Flash column chromatography was performed with Teledyne ISCO CombiFlash EZ Prep chromatography system, employing pre-packed silica gel cartridges (Teledyne ISCO RediSep).

Instrumentation

Proton nuclear magnetic resonance (^{1}H NMR) spectra and carbon nuclear magnetic resonance (^{13}C NMR) spectra were recorded on Bruker AvanceIII HD 2-channel instrument (400 MHz/100 MHz) at 23 °C. Proton chemical shifts are expressed in parts per million (ppm, δ scale) and are referenced to residual protium in the NMR solvent ($CHCl_3$: δ 7.26, D_2HSOCD_3: δ 2.50). Data are represented as follows: chemical shift, multiplicity (s = singlet, d = doublet, t = triplet, q = quartet, dd =

doublet of doublets, dt = doublet of triplets, m = multiplet, br = broad, app = apparent), integration, and coupling constant (J) in Hertz (Hz). High-resolution mass spectra were obtained using a Waters Xevo G2-XS time-of-flight mass spectrometer.

.

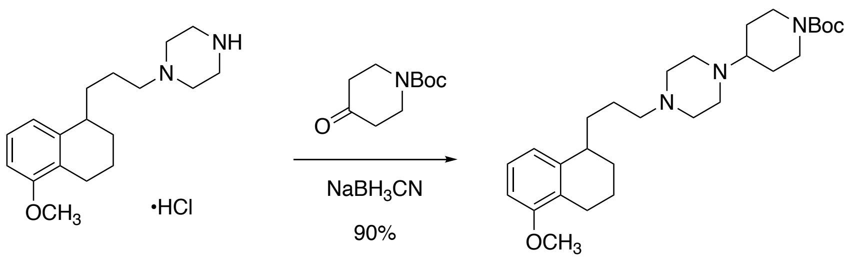

S1•HCl **ZZY-10-051**

ZZY-10-051

N-Boc-4-piperidone (36.8 mg, 0.185 mmol) and sodium cyanoborohydride (11.6 mg, 0.185 mmol) were added sequentially to a stirred suspension of 1-[3-(5-methoxytetralin-1-yl)propyl]piperazine hydrochloride (*58*) (S1•HCl, 20.0 mg, 0.0616 mmol) in methanol (0.50 mL). The mixture was stirred at 23 ºC and the reaction progress was monitored by LC-MS. After 24 h, the reaction mixture was partitioned between saturated aqueous sodium bicarbonate solution (5 mL) and dichloromethane (5 mL). The layers were separated, and the aqueous layer was extracted with dichloromethane (2 x 5 mL). The combined organic layers were dried over sodium sulfate. The dried solution was filtered, and the filtrate was concentrated. The residue was purified by column chromatography (0–10% methanol–dichloromethane, 4-g RediSep(R) Rf column, Teledyne ISCO, Lincoln, NE) to afford the product as a white foam (26.0 mg, 90%).

^{1}H NMR (400 MHz, CDCl3) δ 7.08 (t, J = 7.9 Hz, 1H), 6.77 (d, J = 7.7 Hz, 1H), 6.64 (dd, J = 8.2, 1.1 Hz, 1H), 4.13 (s, 2H), 3.79 (s, 3H), 2.81 – 2.48 (m, 12H), 2.48 – 2.32 (m, 4H), 1.89 – 1.51 (m, 10H), 1.44 (s, 9H), 1.42 – 1.33 (m, 2H).

HRMS (ESI$^+$): Calculated for $[C_{28}H_{45}N_3O_3+H]^+$: 472.3539. Found: 472.3518.

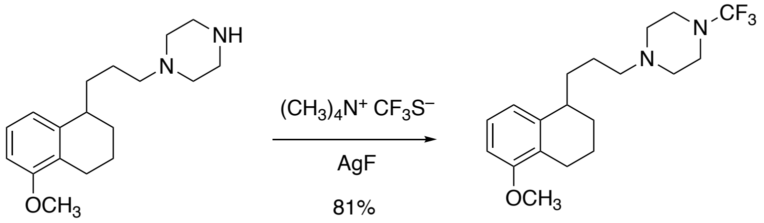

S1 **ZZY-10-061**

ZZY-10-061

An oven-dried one-dram vial was charged with 1-[3-(5-methoxytetralin-1-yl)propyl]piperazine (*58*) (S1, 40.0 mg, 0.139 mmol), acetonitrile (0.35 mL) and a magnetic stir bar. The mixture was sonicated until an even suspension was formed. The solids did not fully dissolve. Tetramethylammonium trifluoromethanethiolate (31.6 mg, 0.180 mmol) was added in one portion, and the mixture was stirred for 15 min at 23 ºC. Silver(I) fluoride (52.8 mg, 0.416 mmol) was then added in a single portion, and the mixture was warmed to 50 ºC and stirred at that temperature for 4 h. At this point, TLC analysis showed complete conversion. The reaction mixture was diluted with ether (10 mL) and filtered through a cotton plug. The filtrate was directly concentrated to afford the product as pale white powder (40.0 mg, 81%).

^{1}H NMR (400 MHz, DMSO-d6) δ 7.10 (t, J = 7.9 Hz, 1H), 6.79 (d, J = 7.7 Hz, 1H), 6.74 (d, J = 8.1 Hz, 1H), 3.97 (d, J = 14.2 Hz, 1H), 3.62 – 3.34 (m, 7H), 3.23 – 2.93 (m, 4H), 2.75 (s, 1H), 1.84 – 1.46 (m, 9H).

HRMS (ESI+): Calculated for [$C_{19}H_{27}F_3N_2O$+H]+: 357.2154. Found: 357.2162.

Correlation analyses

Compound-by-compound phospholipidosis versus SARS-CoV-2 infection correlation analyses were performed using the “correlation” function in GraphPad Prism using raw RT-qPCR values normalized to DMSO from the same experiment at 100%. To model the pooled correlation we used Bayesian inference through the brms R package v2.14.4 (*59*) the log PLD. With weakly informative priors, fitting 107 drug/concentration pairs yielded posterior parameter means and 95% credible interval (95%CI) estimates of IC_{50}: 43 [38, 48]%, hill: -5.6 [-7.0, -4.5], and Sigma 2.0 [0.14, 1.78]. Bayesian leave-one-out R^2 values were computed by using the loo package v2.4.1 (*60*). Analysis and accompanying raw data can be found at https://github.com/momeara/DIPL_SARS-CoV-2.

Literature search for SARS-CoV-2 repurposed antivirals

Twelve major phenotypic antiviral repurposing papers were sourced from the literature (*3*, *8*, *14*–*16*, *24*–*29*). Drug names were selected from each paper based on the author-reported number of in vitro hits at the most strict reported threshold. If an explicit mention of the number of hits was not mentioned, all molecules with demonstrated dose-response inhibition of SARS-CoV-2 were selected. SMILES for each compound were retrieved from PubChem (*61*) using the PubChemPy API (https://pubchempy.readthedocs.io). Using the PubChem canonical SMILES, each molecule’s cLogP was calculated using RDkit-2019.09.3.0 (http://www.rdkit.org), and JChem’s cxcalc command line tool

was used to calculate each molecule's most basic pKa, JChem-15.11.23.0, ChemAxon (https://www.chemaxon.com). Molecules with cLogP ≥ 3 and pKa ≥ 7.4 were considered CADs. ECFP-4-based Tanimoto coefficients (Tcs) were calculated for the list of CADs to all molecules from a list of known phospholipidosis inducers [Citation error], and the maximum Tc for each CAD to a known phospholipidosis inducer was used for filtering the CAD list for known, and highly likely, phospholipidosis inducers (Tc ≥ 0.4).

Pharmacokinetics

Pharmacokinetic experiments were performed by Bienta (Enamine Biology Services) in accordance with Enamine pharmacokinetic study protocols and Institutional Animal Care and Use Guidelines (protocol number 1-2/2020). Plasma pharmacokinetics and lung distribution for amiodarone, sertraline, PB28, tamoxifen, and elacridar were investigated following five intraperitoneal (i.p.) doses of each drug in 21 male mice per drug condition plus 1 mouse per vehicle condition (106 mice per drug experiment total). CD-1 mice were used for PB28, amiodarone, elacridar, and sertraline studies and C57BL/6 mice were used for tamoxifen studies. The formulations for each compound was as follows: amiodarone- PG-PEG400 (80%:20%); sertraline- DMA-PEG400-physiological saline (20%:20%:60%); PB28- PG-PEG400 (80%:20%); tamoxifen- corn oil (100%); Elacridar- DMA-PEG400-2HPβCD-water (25%:25%:25%:25%). Mice were injected i.p. with 2,2,2-tribromoethanol at the dose of 150 mg/kg prior to drawing blood. Blood collection was performed from the orbital sinus in microtainers containing K2EDTA at 0, 5, 15, 30, 60, 120, 360, and 1440 minutes after drug injection. Animals were sacrificed by cervical dislocation after the blood samples collection. After this, lung samples were collected and weighted. All samples were immediately processed, flash-frozen and stored at -70°C until subsequent analysis.

Plasma samples (50 µL) were mixed with 200 µL of internal standard (IS) solution. After mixing by pipetting and centrifuging for 4 min at 6000 rpm, 0.5 µL of each supernatant was injected into the LC-MS/MS system. Solution of each compound (400 ng/ml in acetonitrile-methanol mixture, 1:1, v/v) was used as IS for drug quantification in plasma samples. Lung samples were dispersed in 3.5 volumes of IS400(90) using stainless steel beads (115 mg ± 5 mg) in The Bullet Blender® homogenizer for 90 sec at speed 10. After this, the samples were centrifuged for 4 min at 14,000 rpm, and 0.5 µL of each supernatant was injected into the LC-MS/MS system. Solution of a reference

compound (sertraline or amiodarone; 400 ng/ml in water-methanol mixture, 1:9, v/v) was used as IS for quantification of the drugs in lung samples.

Analyses of plasma and lung samples were conducted at Enamine/Bienta. The concentrations of drugs in plasma and lung samples were determined using high performance liquid chromatography/tandem mass spectrometry (HPLC-MS/MS). The Shimadzu HPLC system used consists of two isocratic pumps LC-10ADvp, an autosampler SIL-30AC MP, a sub-controller FCV-20AHs and a degasser DGU-14A. Mass spectrometric analysis was performed using a 4000 Q TRAP instrument from MDS Sciex (Canada) with an electro-spray (ESI) interface. The data acquisition and system control was performed using Analyst 1.6.3 software from AB Sciex. The concentrations of the test compound below the lower limit of quantitation (LLOQ, amiodarone: 5 ng/mL for plasma and 7 ng/g for lung; sertraline: 10 ng/mL for plasma and 17.5 ng/g for lung; PB28: 5 ng/mL for plasma and 20 ng/g for lung; tamoxifen: 10 ng/mL for plasma and 8 ng/g for lung; elacridar: 5 ng/mL for plasma and 35 ng/g for lung) were designated as zero. The pharmacokinetic data analysis was performed using noncompartmental, bolus injection or extravascular input analysis models in WinNonlin 5.2 (PharSight). Data below LLOQ were presented as missing to improve validity of T½ calculations.

<u>3-day dosing animal models of SARS-CoV-2 infection experiments</u>

All the antiviral animal studies were performed in an animal biosafety level 3 (BSL3) facility at the Icahn school of Medicine in Mount Sinai Hospital, New York City. All work was conducted under protocols approved by the Mt. Sinai Institutional Animal Care and Use Committee (IACUC). We used a model of BALB/c mice transduced intranasally with an adenovirus expressing human ACE2 (hACE2) for all acute dosing experiments as described previously (*62*). We used 29 female 12-week-old specific pathogen–free BALB/c mice (the Jackson laboratory strain 000651). Five days prior to infection with SARS-CoV-2, BALB/c mice were infected intranasally with $2.5x10^8$ PFU of an adenovirus carrying the gene for hACE2. Viral seed stocks for non-replicating E1/E3 deleted viral vectors based on human adenovirus type-5 expressing the human angiotensin-converting enzyme 2 (Ad-ACE2) receptor under the control of a CMV promoter, were obtained from the Iowa Viral Vector Core Facility. Viral stocks were amplified to high titers following infection of T-Rex TM-293 cells and purification using two sequential rounds of cesium chloride (CsCl) ultracentrifugation, as described previously (*63*, *64*). The infectious titer was determined using a tissue culture infectious dose-50

($TCID_{50}$) end-point dilution assay, and physical particle titer quantified by micro-bicinchoninic acid (microBCA) protein assay, both described previously (*63*). Remdesivir was administered subcutaneously (s.c.) while the other five remaining drugs were injected intraperitoneally (i.p.), with amiodarone and elacridar dosed once per day and remdesivir, PB28, tamoxifen, and sertraline being dosed twice per day, for a total of 3 days, consistent with their pharmacokinetic profiles. We administered vehicle (PBS, i.p., twice-daily) or drug treatments, two hours before intranasal infection with 1×10^4 PFU of SARS-CoV-2 in 50 µL of PBS. Mice were anesthetized with a mixture of ketamine/xylazine before each intranasal infection. Three days post infection (dpi) animals were humanely euthanized. Whole left lungs were harvested and homogenized in PBS with silica glass beads then frozen at −80°C for viral titration via $TCID_{50}$. Briefly, infectious supernatants were collected at 48 hours post infection and frozen at −80°C until later use. Infectious titers were quantified by limiting dilution titration using Vero E6 cells. Briefly, Vero E6 cells were seeded in 96-well plates at 20,000 cells/well. The next day, SARS-CoV-2-containing supernatant was applied at serial 10-fold dilutions ranging from 10^{-1} to 10^{-6} and, after 5 days, viral cytopathic effect (CPE) was detected by staining cell monolayers with crystal violet. $TCID_{50}$/mL were calculated using the method of Reed and Muench. GraphPad Prism version 8.0.0 (San Diego, CA) was used to determine differences in lung titers between treatments and vehicle using the Kruskal-Wallis test with Dunn's multiple comparison correction.

<u>15-day dosing animal models of SARS-CoV-2 infection experiments</u>

All the antiviral animal studies were performed in animal biosafety level 3 (BSL3) facility at the Icahn school of Medicine in Mount Sinai Hospital, New York City. All work was conducted under protocols approved by the Institutional Animal Care and Use Committee (IACUC). SARS-CoV-2 isolate USA-WA1/2020 (BEI resources; NR-52281), referred in this report as WT-SARS-CoV-2, was used to challenge mice intranasally. A variant of virus (termed MA- SARS-CoV-2) was obtained after series of passaging in different backgrounds of laboratory mice as well as mACE-2 expressing VeroE6 cells (*65*). Briefly, the virus was serially passaged every 2 days via intranasal inoculation of the virus in 50 µL volume derived from the spun-down supernatants of lung homogenates. The mouse adaptation of the SARS-CoV-2 variant was studied in C57Bl6, BALB/c and 129S1/SVMJ (termed 129 for simplicity) mice models. Viral stocks were sequenced after propagation to verify the integrity of the viral genome with the mutations associated with mouse adaptation.

We utilized female 8-week-old specific pathogen–free 129 mice (the Jackson laboratory strain 002448). The amiodarone, vehicle, and remdesivir groups each had 6 mice. Dosing schemes were as follows; Group 1: 50 mg/kg intraperitoneal (i.p.) amiodarone, once per day (QD); Group 2: i.p. vehicle QD, Group 3: 50 mg/kg subcutaneous (s.c.) remdesevir, twice per day (BID). Amiodarone and vehicle groups were dosed QD for 12 days prior to infection in order to induce phospholipidosis in the animals and dosing was continued through the course of the experiment. Remdesivir was administered starting 1 hour before intranasal infection with 2.5 × 104 PFU of MA-SARS-CoV-2 in 50 µL of PBS. Mice were anesthetized with a mixture of ketamine/xylazine before each intranasal infection. Mice were weighed daily for signs of pathogenesis. 3 days post infection (dpi) animals were humanely euthanized. The right whole lung of each mouse was harvested and preserved in 10% formalin for later histopathology analysis. The left whole lung of each mouse was harvested and homogenized in PBS with silica glass beads then frozen at −80°C for viral titration via TCID50. Infectious titers were quantified by limiting dilution titration using Vero E6 cells. Briefly, Vero E6 cells were seeded in 96-well plates at 20,000 cells/well. The next day, SARS-CoV-2-containing supernatant was applied at serial 10-fold dilutions ranging from 10−1 to 10−6 and, after 5 days, viral cytopathic effect (CPE) was detected by staining cell monolayers with crystal violet. TCID50/ml were calculated using the method of Reed and Muench. The Prism software (GraphPad) was used to determine differences in lung titers using 2-way ANOVA on log-transformed data. One mouse from the amiodarone group died prior to SARS-CoV-2 infection, leaving only 5 mice in Group 1 for the statistical analyses.

129 mice infected with 2.5 x 104 MA-SARS-CoV-2/WA, treated one daily (QD) or twice-daily (BID) with the indicated drugs, had lungs harvested on day 3 post-infection for histopathology analysis. Paraffin-embedded lung tissue blocks for mouse lungs were cut into 5 µm sections. Sections were stained with hematoxylin and eosin (H&E) and analyzed by HistoWiz Inc. (histowiz.com, Brooklyn, NY). Digital light microscopic scans of whole lung processed in toto were examined by an experienced veterinary pathologist. H&E stained sections of lung from 129 mice were examined by implementing a semi quantitative, 5-point grading scheme (0 - within normal limits, 1 - mild, 2 - moderate, 3 - marked, 4 - severe) that took into account four different histopathological parameters: 1) perivascular inflammation 2) bronchial or bronchiolar epithelial degeneration or necrosis 3) bronchial or bronchiolar inflammation and 4) alveolar inflammation. These changes were absent (grade 0) in lungs from uninfected mice from groups that were utilized for this assessment. All

mice from amiodarone, remdesivir, and vehicle treated infected groups exhibited multifocal pulmonary lesions.

COVID-19 clinical trial expenditure analysis

Data for COVID-19 clinical trials were downloaded from the DrugBank (*49*) COVID-19 dashboard (accessed on 2021-02-16). Supplemental information for each compound, including its SMILES and DrugBank ID was downloaded and unpacked from the DrugBank “All drugs” 2021-01-03 release (*66*). A total of 3395 treatments were recorded in the COVID-19 dashboard at the time of download, and 2244 of those annotations were for small molecules from a total of 1490 unique clinical trials worldwide. Using the DrugBank annotated SMILES, each molecule’s cLogP was calculated using RDkit-2019.09.3.0 (http://www.rdkit.org), and JChem’s cxcalc command line tool was used to calculate each molecule’s most basic pKa, JChem-15.11.23.0, ChemAxon (https://www.chemaxon.com). Molecules with cLogP ≥ 3 and pKa ≥ 7.4 were considered CADs. Azithromycin, a non-CAD phospholipidosis inducer, was also included in the CAD dataset due to its shared mechanism of action with CAD antivirals whereas fluvoxamine, a CAD known to act through immune-mediated processes was excluded from the CAD dataset. Additional subsets of these data, including filtering out chloroquine and hydroxychloroquine trials from the CADs, were also analyzed. To quantify the estimated cost of clinical trials, the molecules from a given subset were first filtered for unique clinical trial IDs, then were grouped based on clinical trial phase and multiplied by the average cost of an anti-infective clinical trial in that phase (*50*, *51*). Mixed-phase trials were multiplied by the cost of the more advanced phase only.

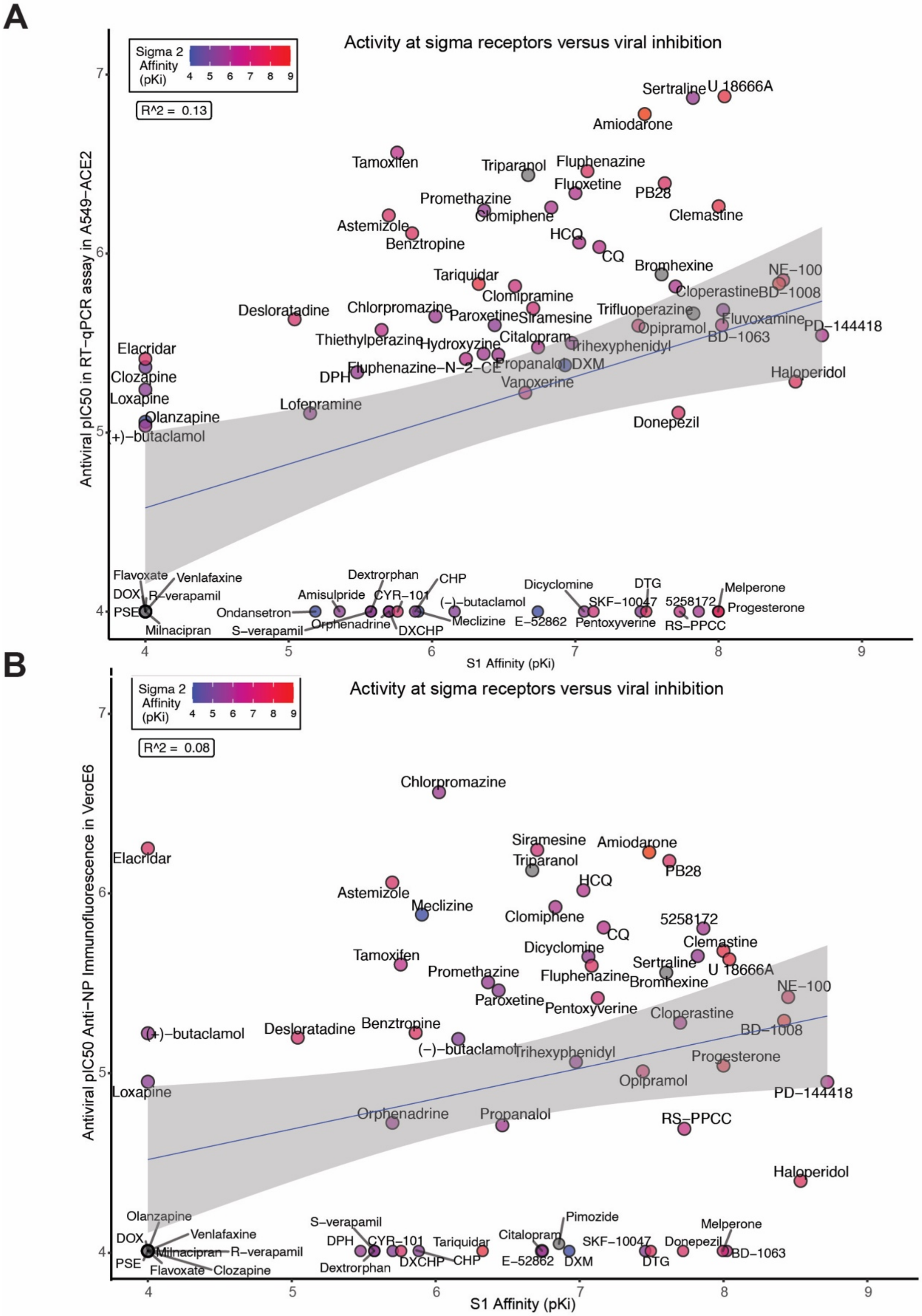

Fig. S1. Correlation analyses for sigma receptor affinity and antiviral activity. A. pKi at Sigma-1 was correlated with pIC50 in the RT-qPCR assay in A549-ACE2 cells. pKi at Sigma-2 is denoted by the colors. **B**. pKi at Sigma-1 was correlated with pIC50 in the anti-NP immunofluorescence viral infectivity assay in VeroE6 cells. Abbreviations: CQ: chloroquine; HCQ: hydroxychloroquine; DOX: doxylamine; PSE: pseudephedrine; CHP: chlorpheniramine; DXCHP: dexchlorpheniramine; DXM: dextromethorphan; DPH: diphenhydramine; DTG: ditolylguanidine.

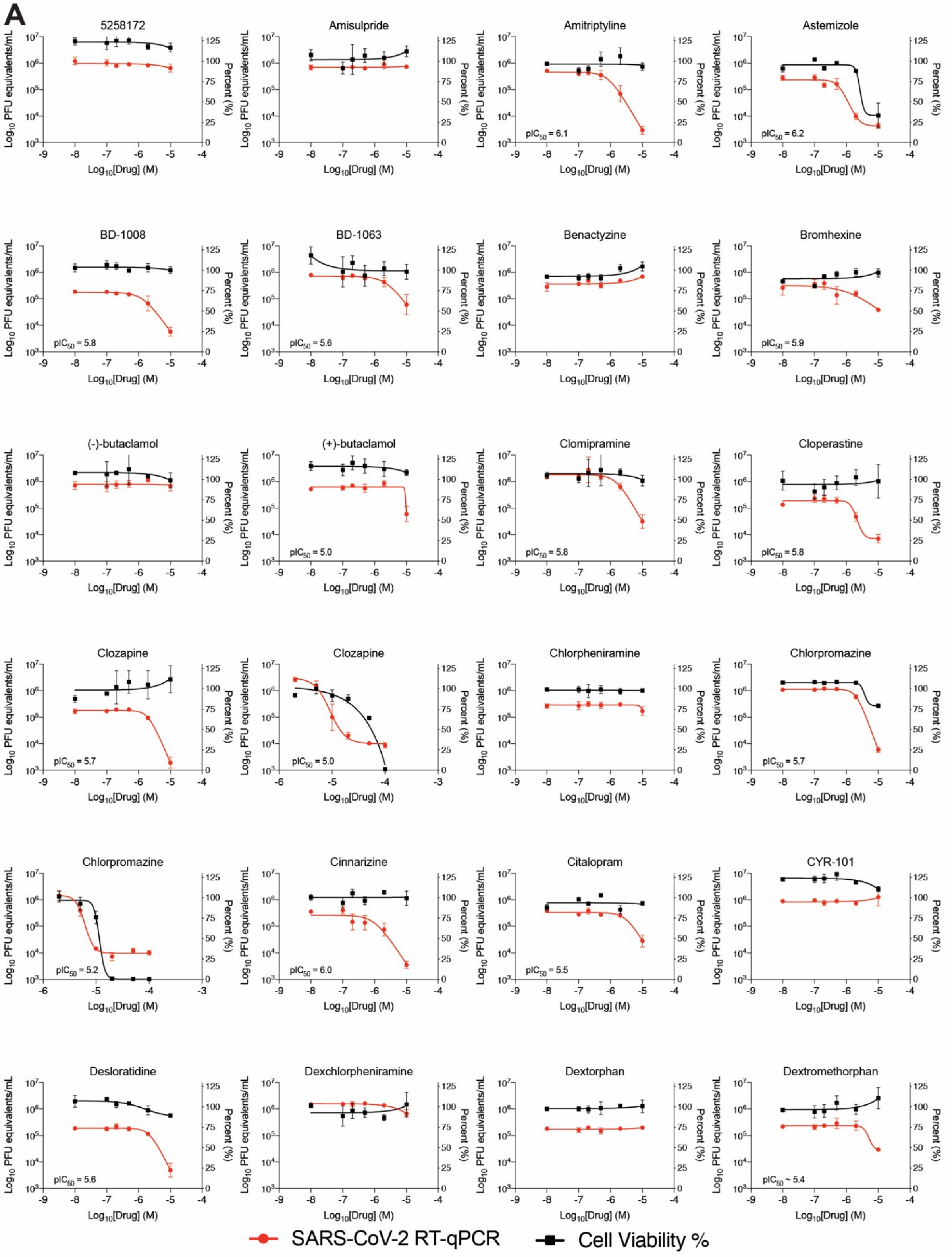

A
5258172
Amisulpride
Amitriptyline
pIC$_{50}$ = 6.1
Astemizole
pIC$_{50}$ = 6.2
BD-1008
pIC$_{50}$ = 5.8
BD-1063
pIC$_{50}$ = 5.6
Benactyzine
Bromhexine
pIC$_{50}$ = 5.9
(-)-butaclamol
(+)-butaclamol
pIC$_{50}$ = 5.0
Clomipramine
pIC$_{50}$ = 5.8
Cloperastine
pIC$_{50}$ = 5.8
Clozapine
pIC$_{50}$ = 5.7
Clozapine
pIC$_{50}$ = 5.0
Chlorpheniramine
Chlorpromazine
pIC$_{50}$ = 5.7
Chlorpromazine
pIC$_{50}$ = 5.2
Cinnarizine
pIC$_{50}$ = 6.0
Citalopram
pIC$_{50}$ = 5.5
CYR-101
Desloratidine
pIC$_{50}$ = 5.6
Dexchlorpheniramine
Dextorphan
Dextromethorphan
pIC$_{50}$ ~ 5.4
Log$_{10}$ PFU equivalents/mL
Percent (%)
Log$_{10}$[Drug] (M)
SARS-CoV-2 RT-qPCR
Cell Viability %

A cont.

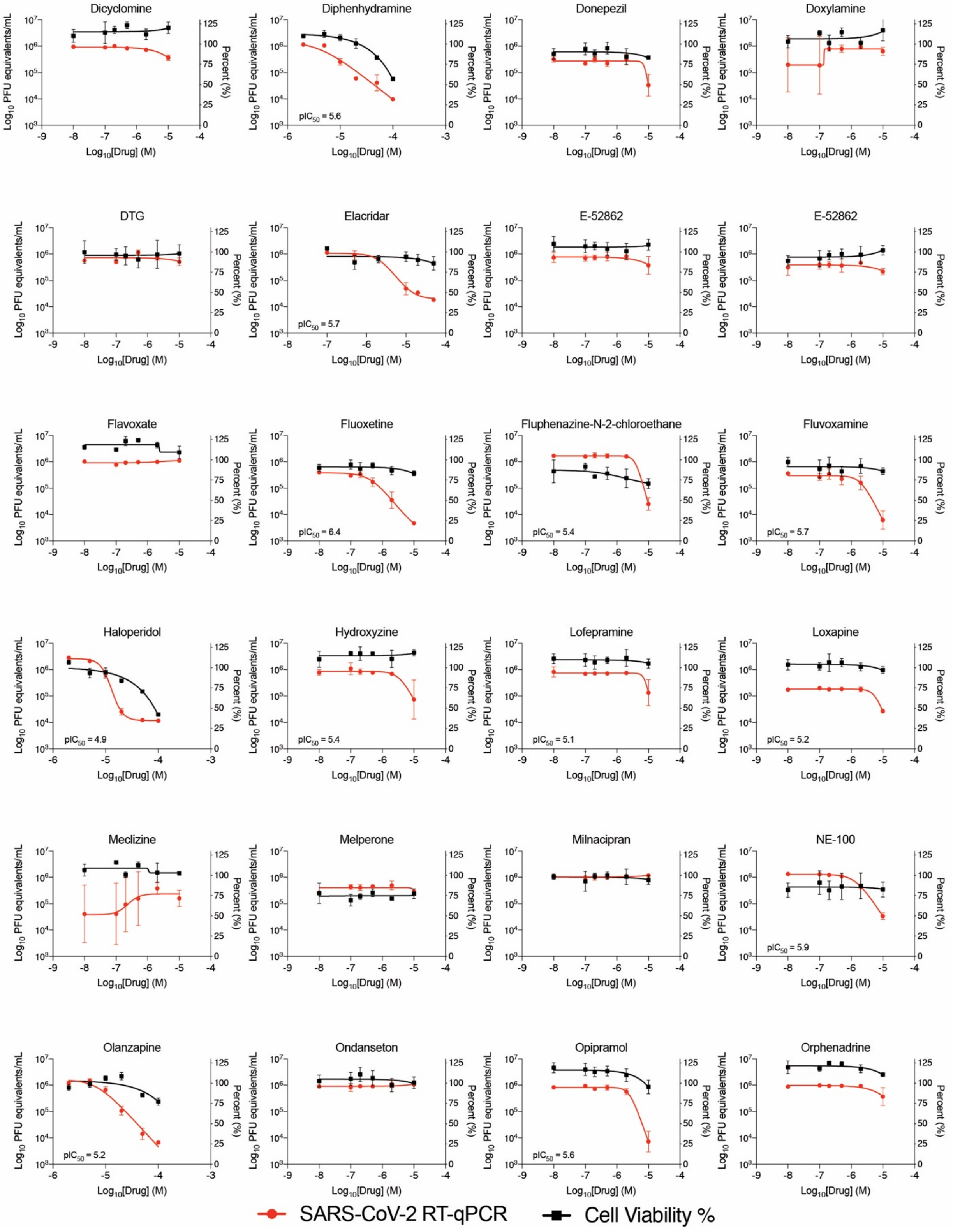
Dicyclomine
Diphenhydramine
pIC$_{50}$ = 5.6
Donepezil
Doxylamine
DTG
Elacridar
pIC$_{50}$ = 5.7
E-52862
E-52862
Flavoxate
Fluoxetine
pIC$_{50}$ = 6.4
Fluphenazine-N-2-chloroethane
pIC$_{50}$ = 5.4
Fluvoxamine
pIC$_{50}$ = 5.7
Haloperidol
pIC$_{50}$ = 4.9
Hydroxyzine
pIC$_{50}$ = 5.4
Lofepramine
pIC$_{50}$ = 5.1
Loxapine
pIC$_{50}$ = 5.2
Meclizine
Melperone
Milnacipran
NE-100
pIC$_{50}$ = 5.9
Olanzapine
pIC$_{50}$ = 5.2
Ondanseton
Opipramol
pIC$_{50}$ = 5.6
Orphenadrine
Log$_{10}$ PFU equivalents/mL
Percent (%)
Log$_{10}$[Drug] (M)
SARS-CoV-2 RT-qPCR
Cell Viability %

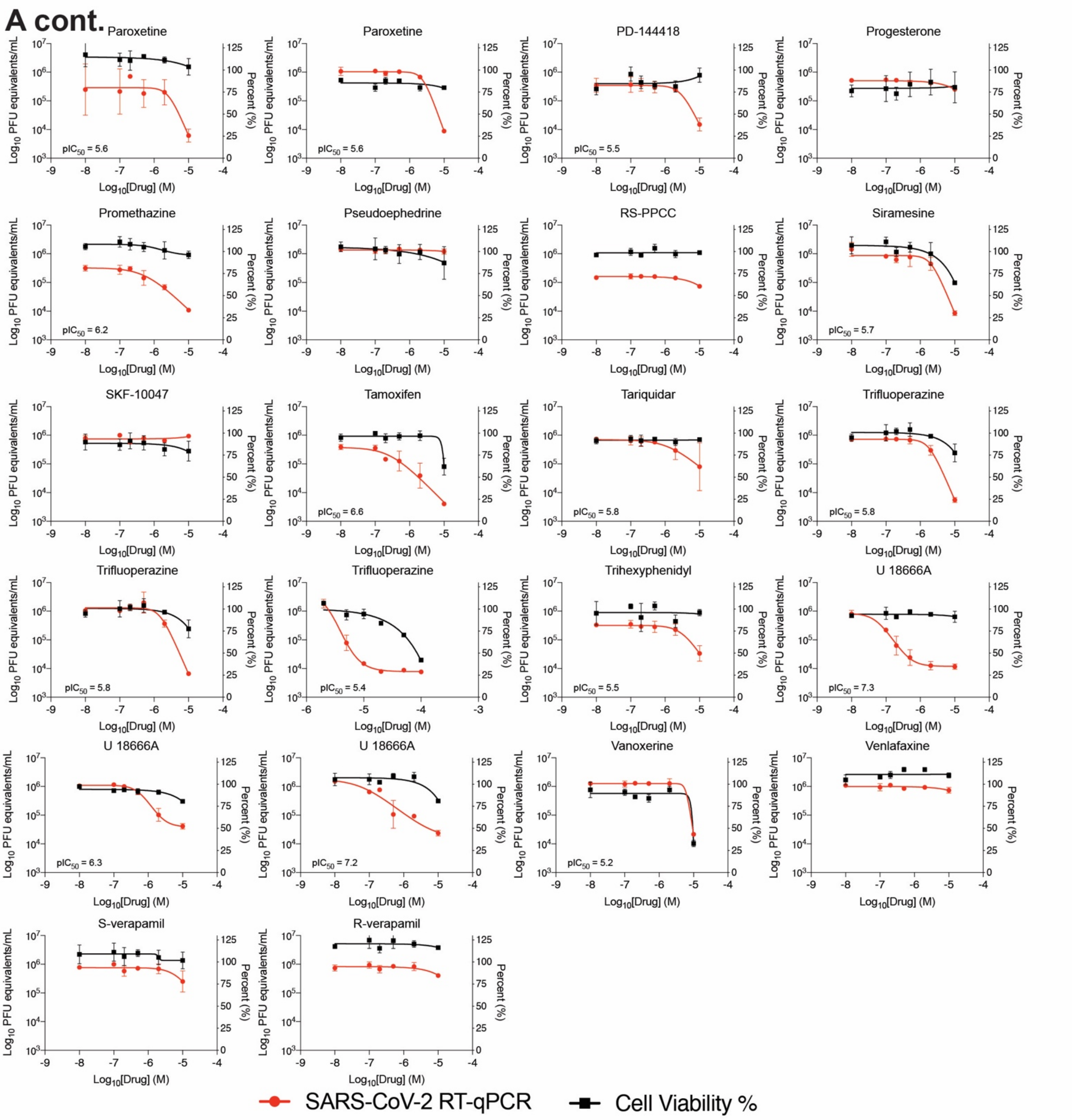

Fig. S2. Dose response curves for a set of cationic amphiphilic drugs in an RT-qPCR viral infectivity assay. A. Viral infectivity and cell viability data for a subset of literature-identified cationic amphiphilic drugs in VeroE6 cells. Data shown are mean ± SD from three biological replicates. Independent experiments are shown as separate graphs when available.

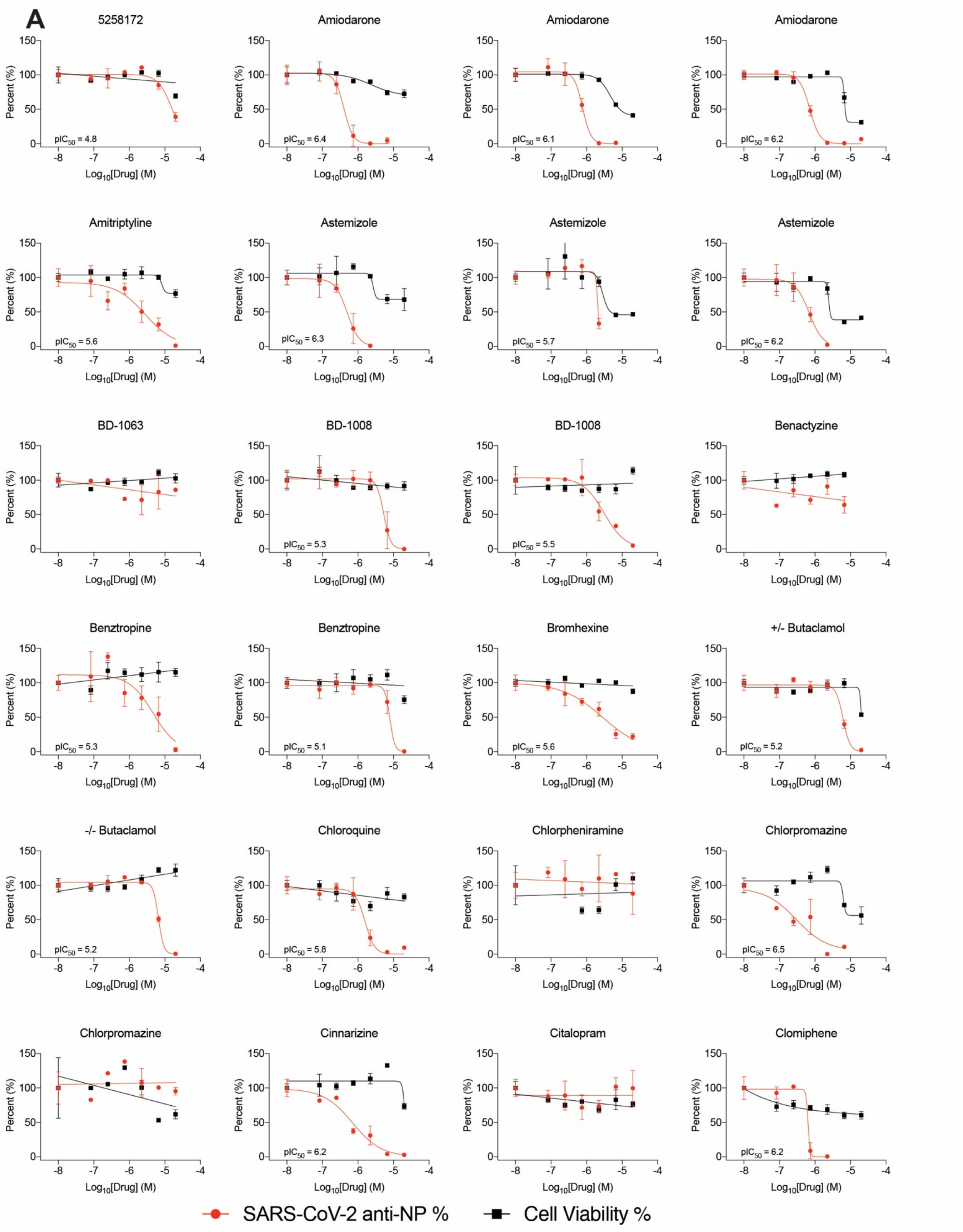
A
5258172
pIC50 = 4.8
Amiodarone
pIC50 = 6.4
Amiodarone
pIC50 = 6.1
Amiodarone
pIC50 = 6.2
Amitriptyline
pIC50 = 5.6
Astemizole
pIC50 = 6.3
Astemizole
pIC50 = 5.7
Astemizole
pIC50 = 6.2
BD-1063
BD-1008
pIC50 = 5.3
BD-1008
pIC50 = 5.5
Benactyzine
Benztropine
pIC50 = 5.3
Benztropine
pIC50 = 5.1
Bromhexine
pIC50 = 5.6
+/- Butaclamol
pIC50 = 5.2
-/- Butaclamol
pIC50 = 5.2
Chloroquine
pIC50 = 5.8
Chlorpheniramine
Chlorpromazine
pIC50 = 6.5
Chlorpromazine
Cinnarizine
pIC50 = 6.2
Citalopram
Clomiphene
pIC50 = 6.2
Percent (%)
Log10[Drug] (M)
SARS-CoV-2 anti-NP %
Cell Viability %

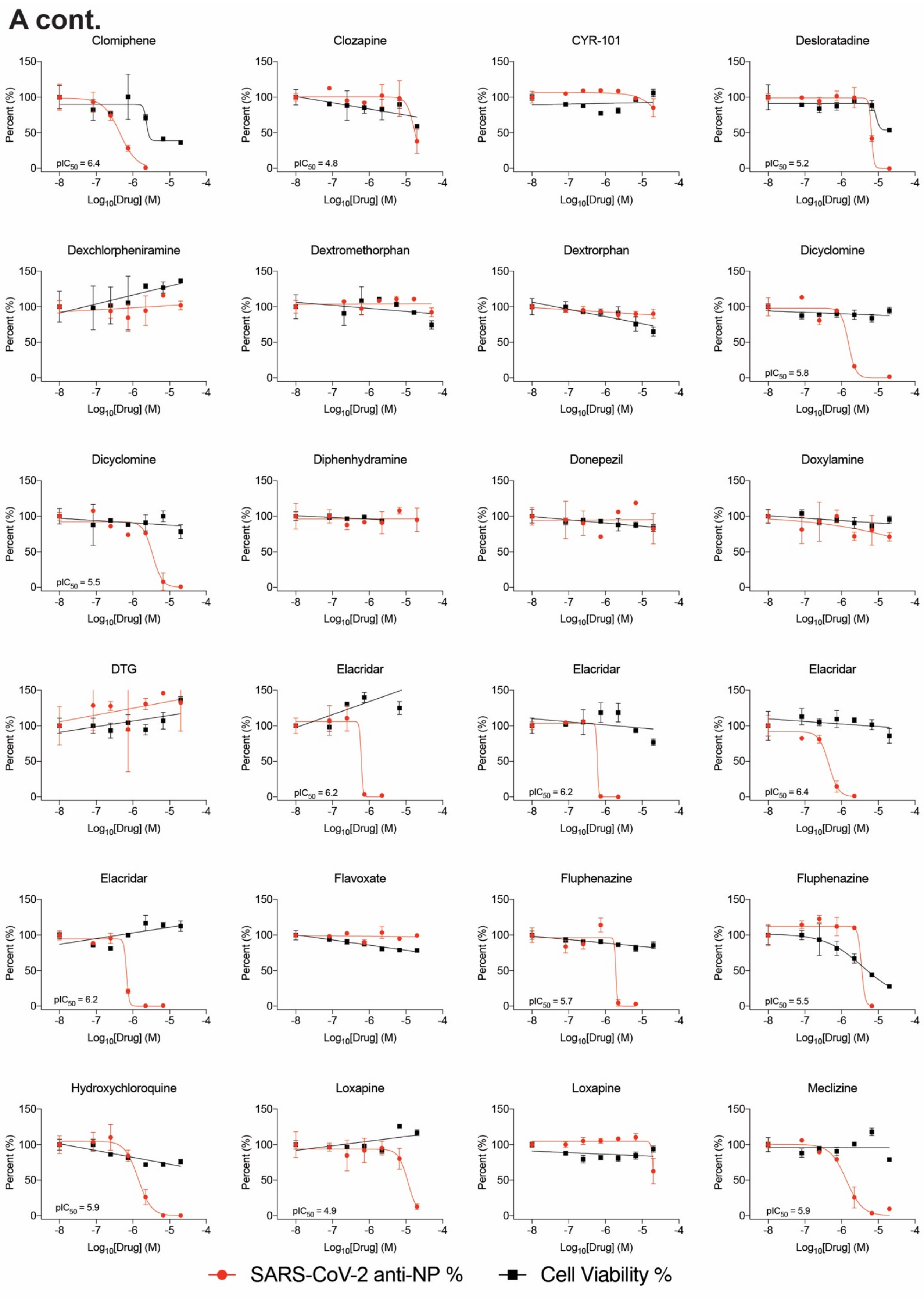
A cont.
Clomiphene
pIC50 = 6.4
Clozapine
pIC50 = 4.8
CYR-101
Desloratadine
pIC50 = 5.2
Dexchlorpheniramine
Dextromethorphan
Dextrorphan
Dicyclomine
pIC50 = 5.8
Dicyclomine
pIC50 = 5.5
Diphenhydramine
Donepezil
Doxylamine
DTG
Elacridar
pIC50 = 6.2
Elacridar
pIC50 = 6.2
Elacridar
pIC50 = 6.4
Elacridar
pIC50 = 6.2
Flavoxate
Fluphenazine
pIC50 = 5.7
Fluphenazine
pIC50 = 5.5
Hydroxychloroquine
pIC50 = 5.9
Loxapine
pIC50 = 4.9
Loxapine
Meclizine
pIC50 = 5.9
Percent (%)
Log10[Drug] (M)
SARS-CoV-2 anti-NP %
Cell Viability %

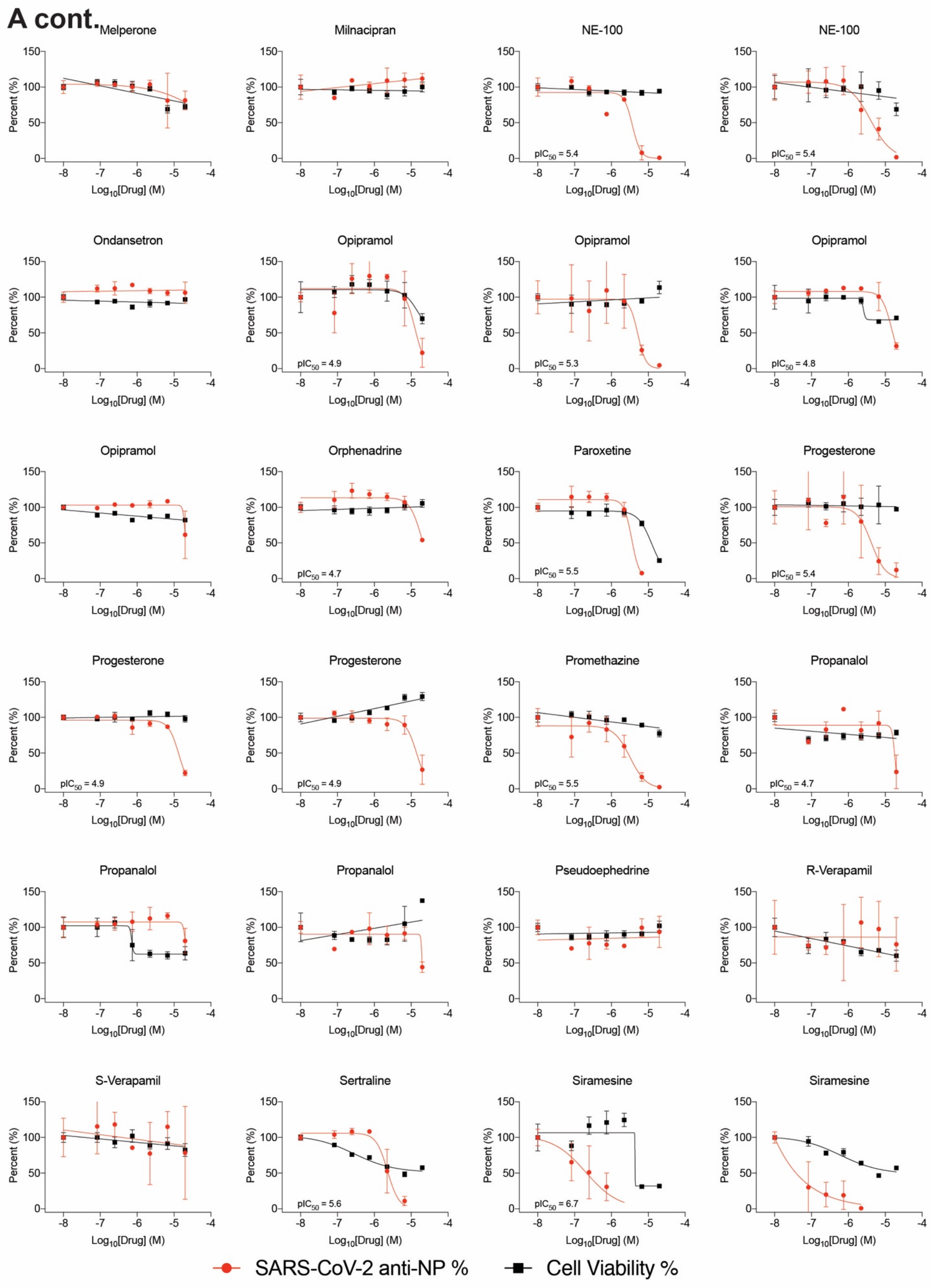
A cont.
Melperone
Milnacipran
NE-100
pIC50 = 5.4
NE-100
pIC50 = 5.4
Ondansetron
Opipramol
pIC50 = 4.9
Opipramol
pIC50 = 5.3
Opipramol
pIC50 = 4.8
Opipramol
Orphenadrine
pIC50 = 4.7
Paroxetine
pIC50 = 5.5
Progesterone
pIC50 = 5.4
Progesterone
pIC50 = 4.9
Progesterone
pIC50 = 4.9
Promethazine
pIC50 = 5.5
Propanalol
pIC50 = 4.7
Propanalol
Propanalol
Pseudoephedrine
R-Verapamil
S-Verapamil
Sertraline
pIC50 = 5.6
Siramesine
pIC50 = 6.7
Siramesine
Percent (%)
Log10[Drug] (M)
SARS-CoV-2 anti-NP %
Cell Viability %

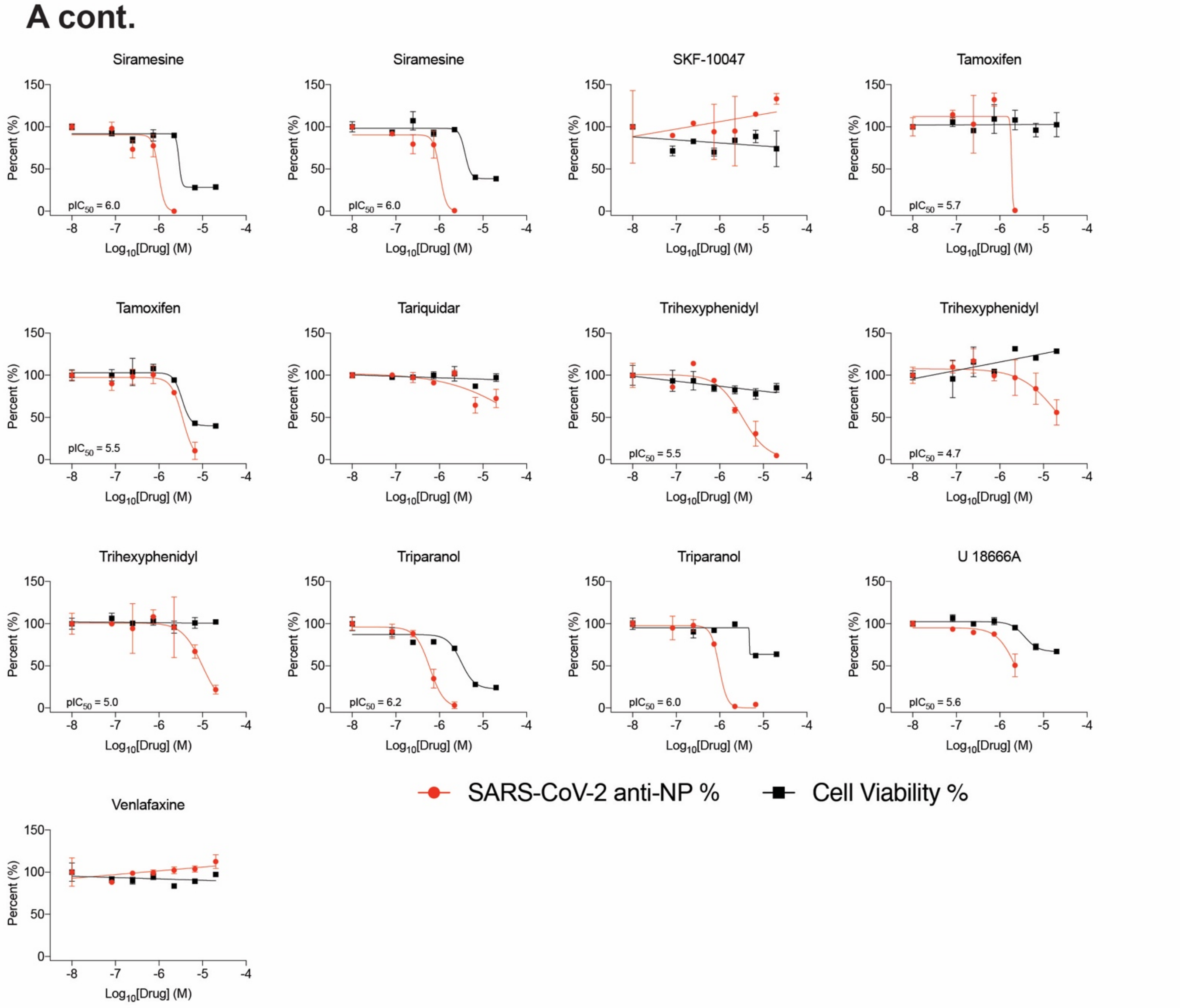

Fig. S3. Dose response curves for a set of cationic amphiphilic drugs (CADs) in an anti-NP immunofluorescence viral infectivity assay. A. Viral infectivity and cell viability data for a subset of literature-identified CADs in VeroE6 cells. Data shown are mean ± SD from three biological replicates. Independent experiments are shown as separate graphs when available.

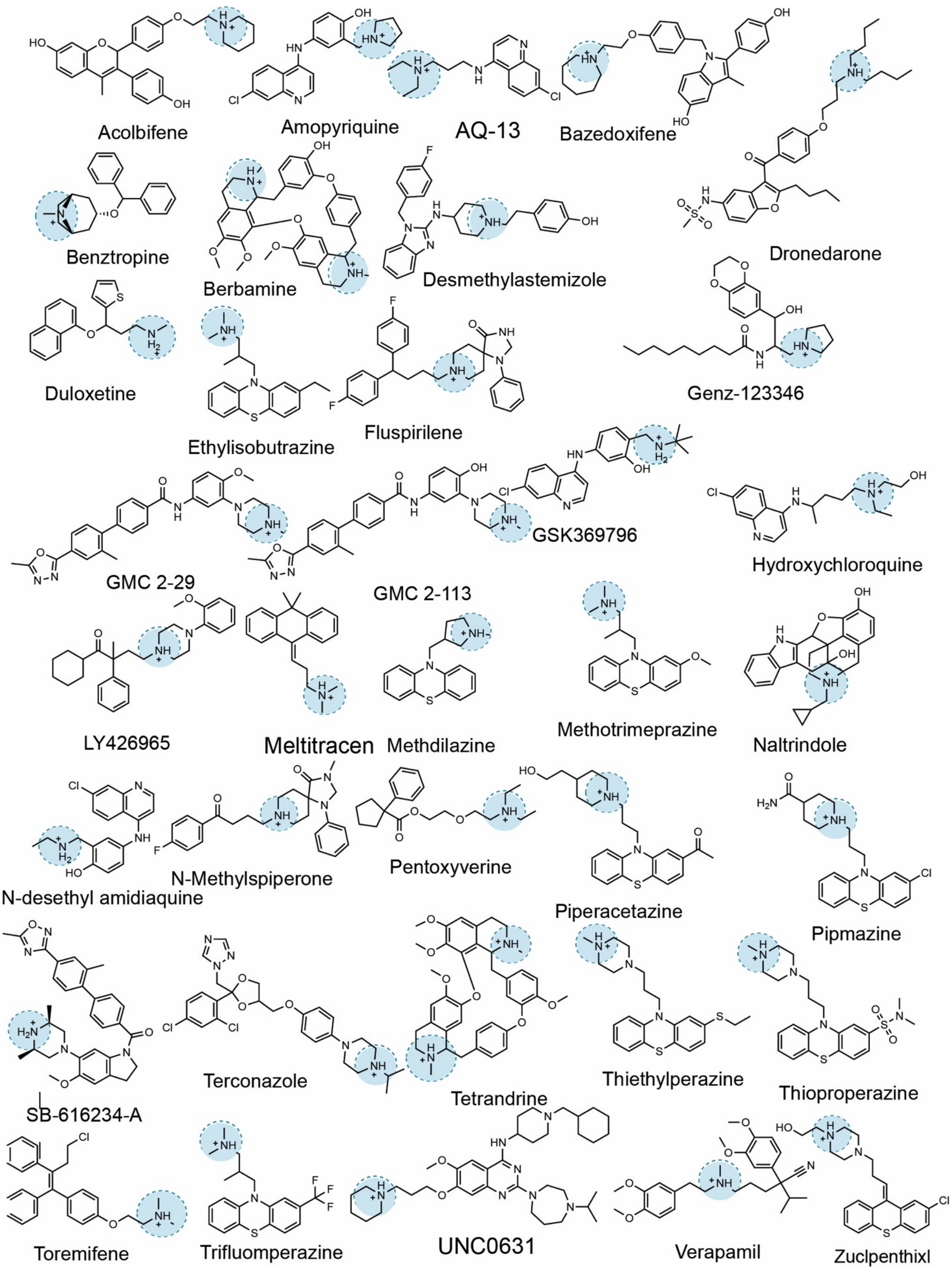

Fig. S4. Example cationic amphiphilic drugs identified from SARS-CoV-2 drug repurposing literature predicted to induce phospholipidosis.

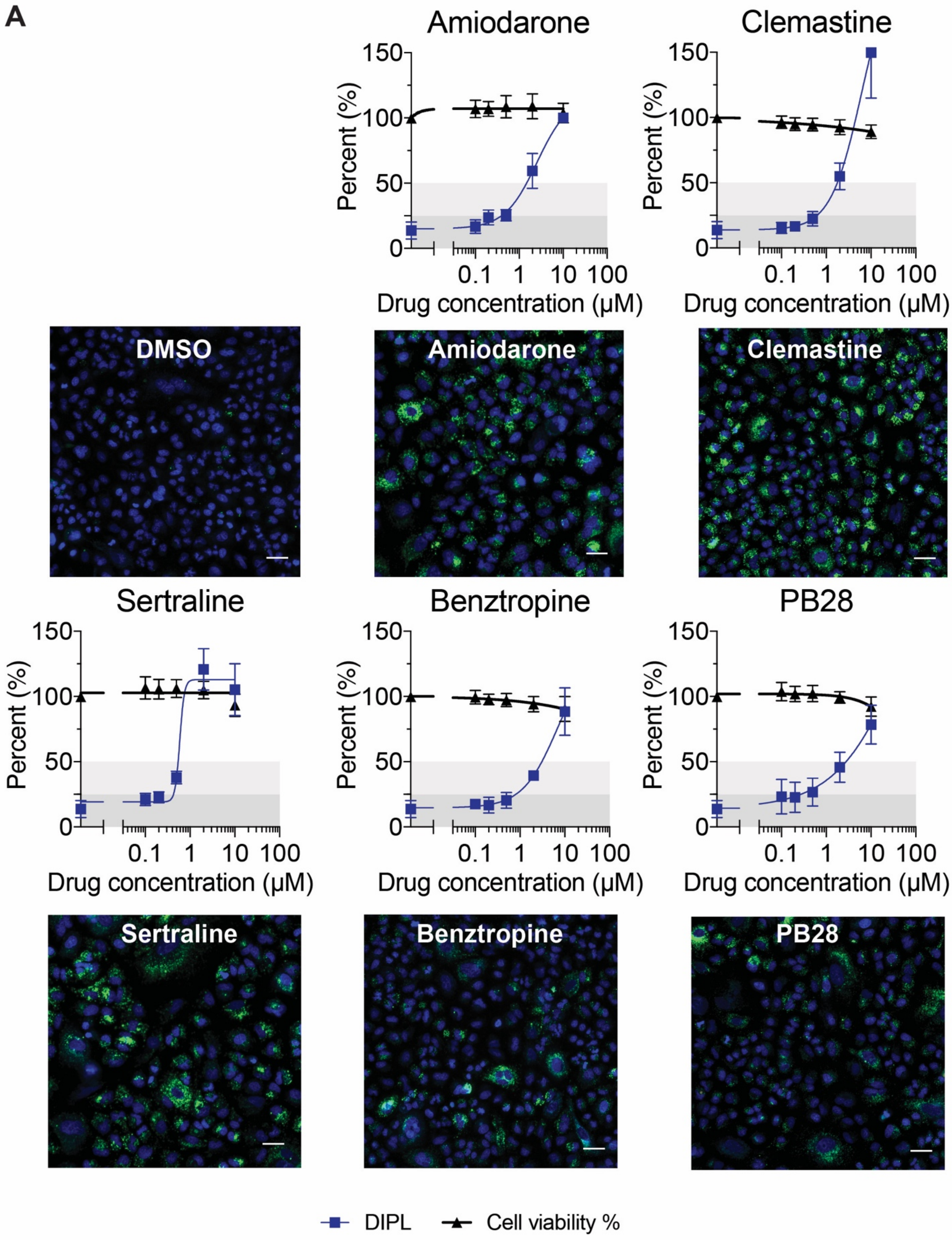

A
Amiodarone
Clemastine
Percent (%)
150
100
50
0
0.1
1
10
100
Drug concentration (μM)
DMSO
Amiodarone
Clemastine
Sertraline
Benztropine
PB28
DIPL
Cell viability %

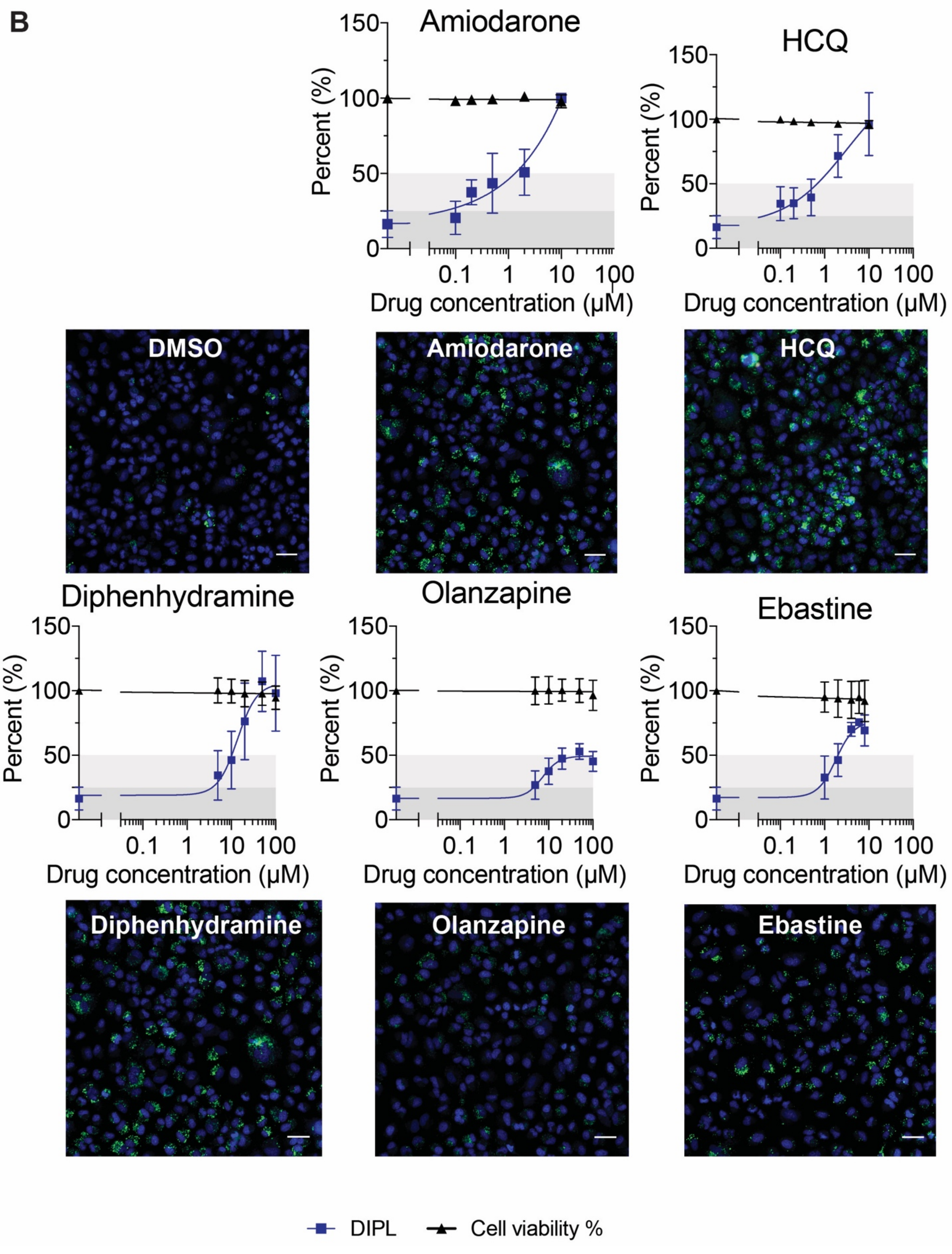

B
Amiodarone
HCQ
Percent (%)
Drug concentration (μM)
DMSO
Amiodarone
HCQ
Diphenhydramine
Olanzapine
Ebastine
DIPL
Cell viability %

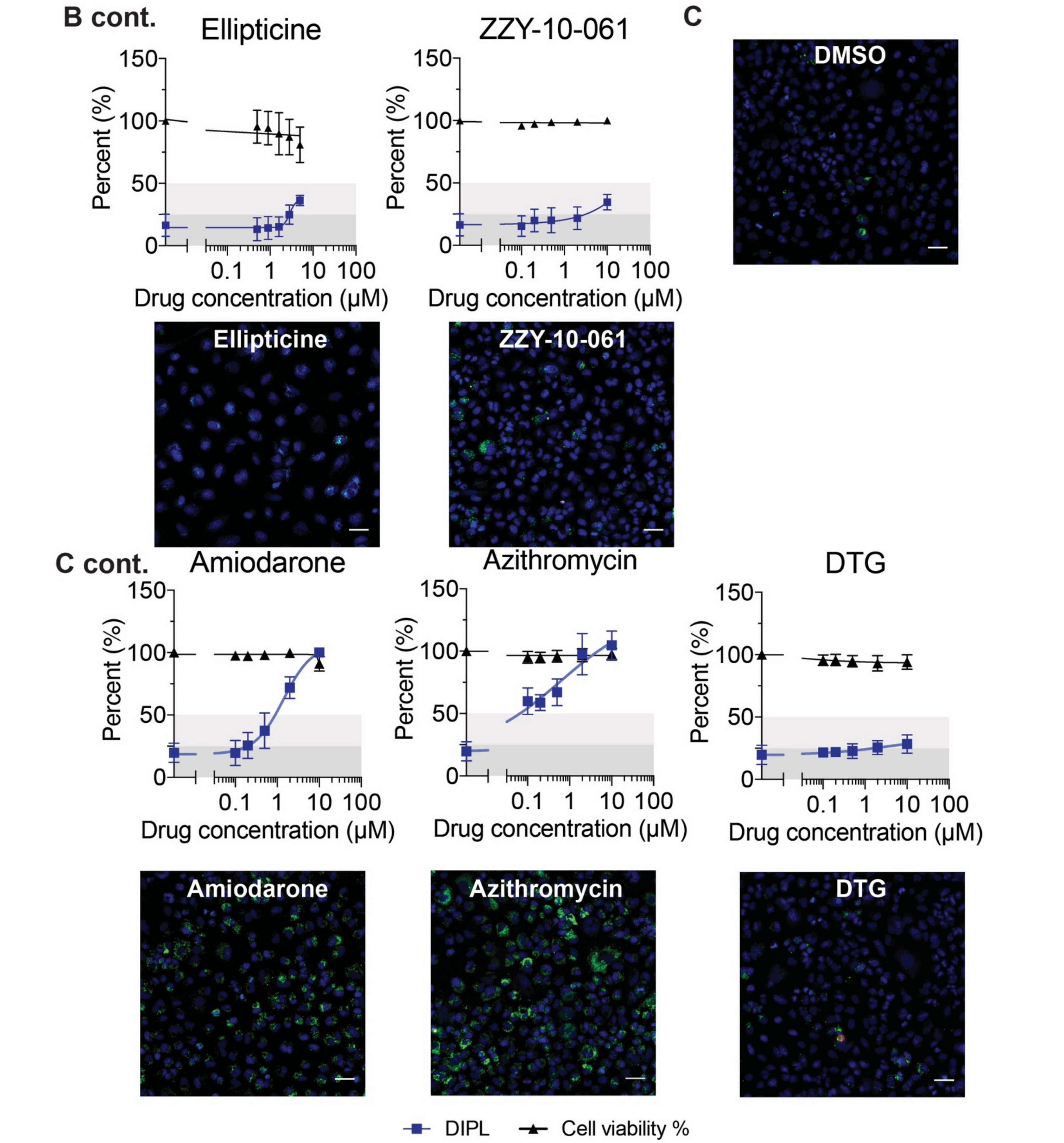

Fig. S5. Dose response curves for drugs measured in the phospholipidosis and cell viability assays and plate images at top tested concentrations. Batch 1- **A.**, batch 2- **B.**, and batch 3- **C.** Blue = Hoechst nuclei staining, Green = NBD-PE phospholipid staining, Red = EthD-2 staining for dead cells. Dose response of NBD-PE aggregation was normalized to DMSO and 10 μM amiodarone from the same batch, and cell viability was normalized to DMSO. Data shown are pooled means ± SD from three independent experiments each with three biological replicates.

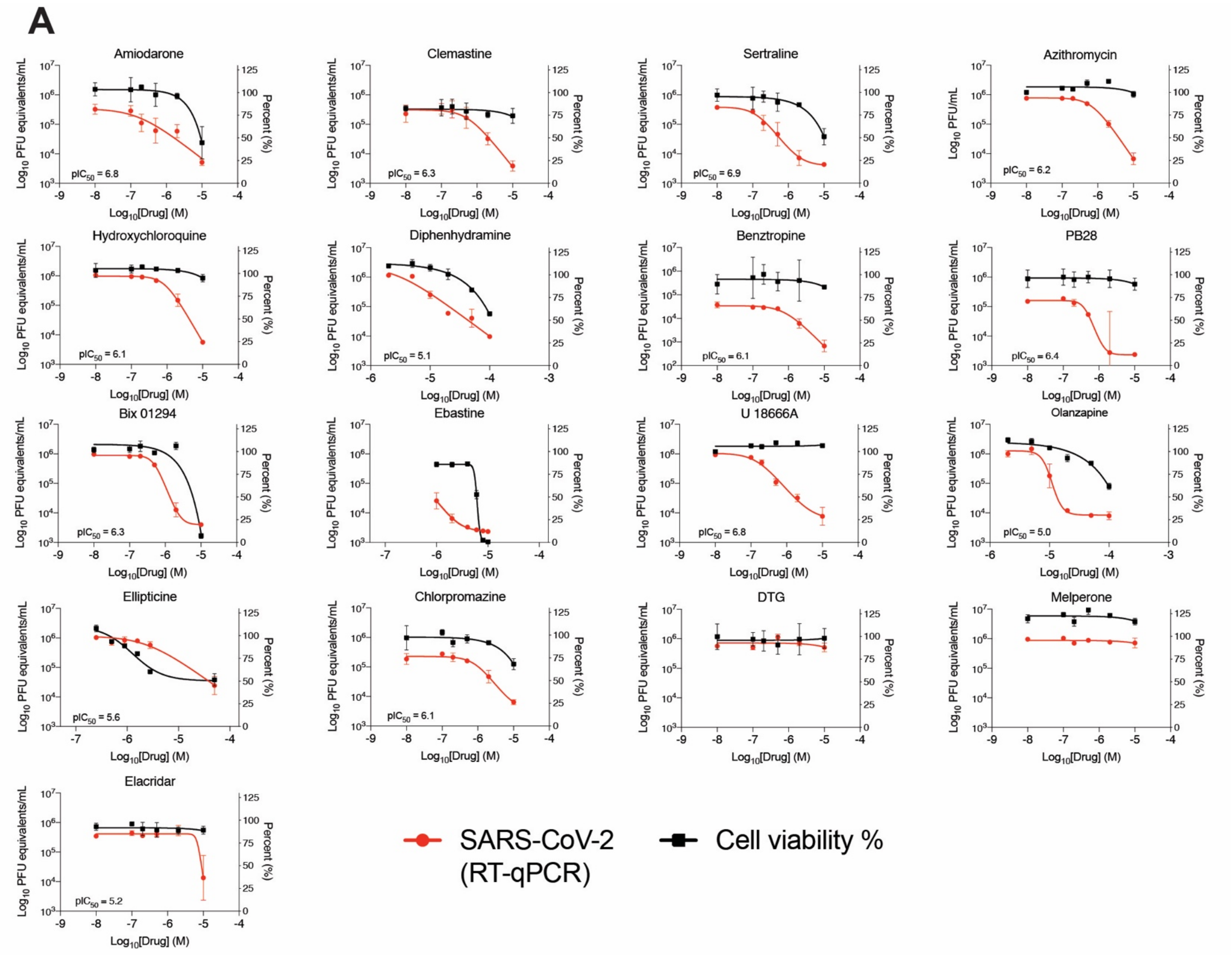

Fig. S6. Dose response curves for cationic amphiphilic drugs in the RT-qPCR viral infectivity assay that were measured for NBD-PE aggregation. A. Viral infectivity and cell viability data for a subset of drugs that were selected for the DIPL correlation analysis. Data shown are mean ± SD from three biological replicates. The concentrations from these experiments match what was tested in the NBD-PE assay. Data for amiodarone, chlorpromazine, and hydroxychloroquine are reprinted from Gordon *et al.* with permission (*14*). Data for ZZY-10-051 and ZZY-10-061 are in **Fig. S8**.

A

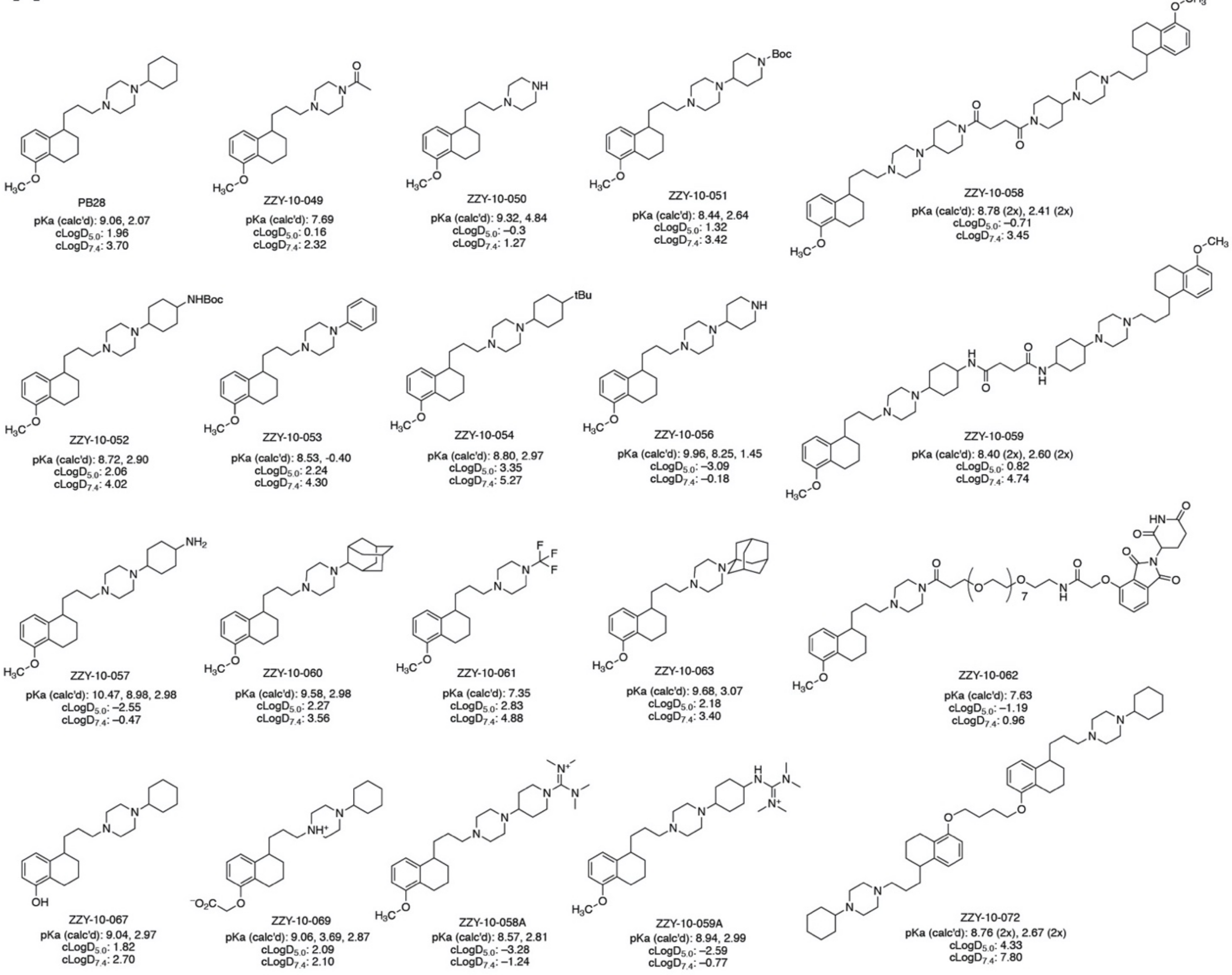

B

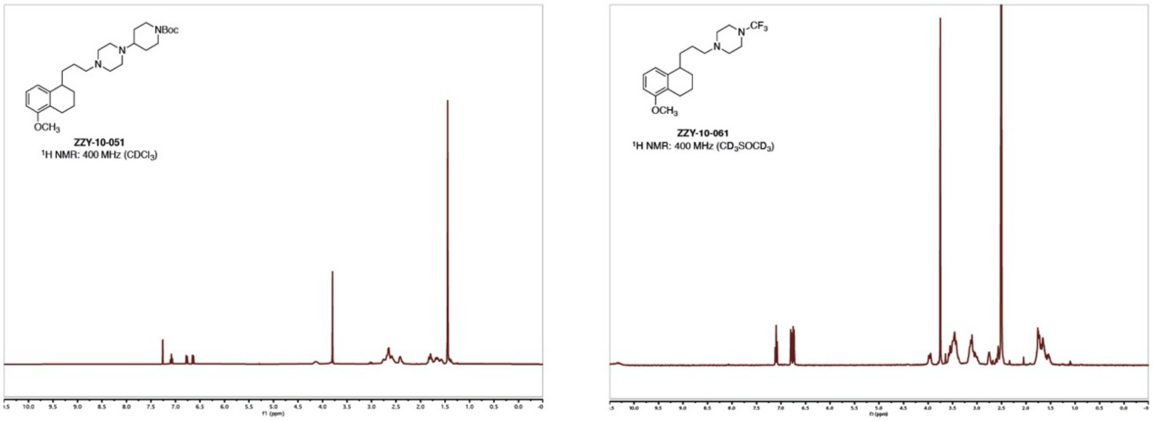

Fig S7. PB28 analog structures. A. Chemical structures of PB28 analogs tested, shown in their neutral form. All compounds were prepared as racemates. Compounds ZZY-10-061, ZZY-10-062, ZZY-10-064, ZZY-10-056, ZZY-10-057, ZZY-10-058, ZZY-10-059 and ZZY-10-072 contain mixtures of diastereomers that were not resolved or separated. With the exception of ZZY-10-061 and ZZY-10-062, all compounds were prepared as HCl salts by acidification of their neutral forms with an ethereal solution of hydrogen chloride, or by lyophilization of their aqueous solutions in 50 mM HCl. **B.** ^{1}H NMR spectra for ZZY-10-051 and ZZY-10-061.

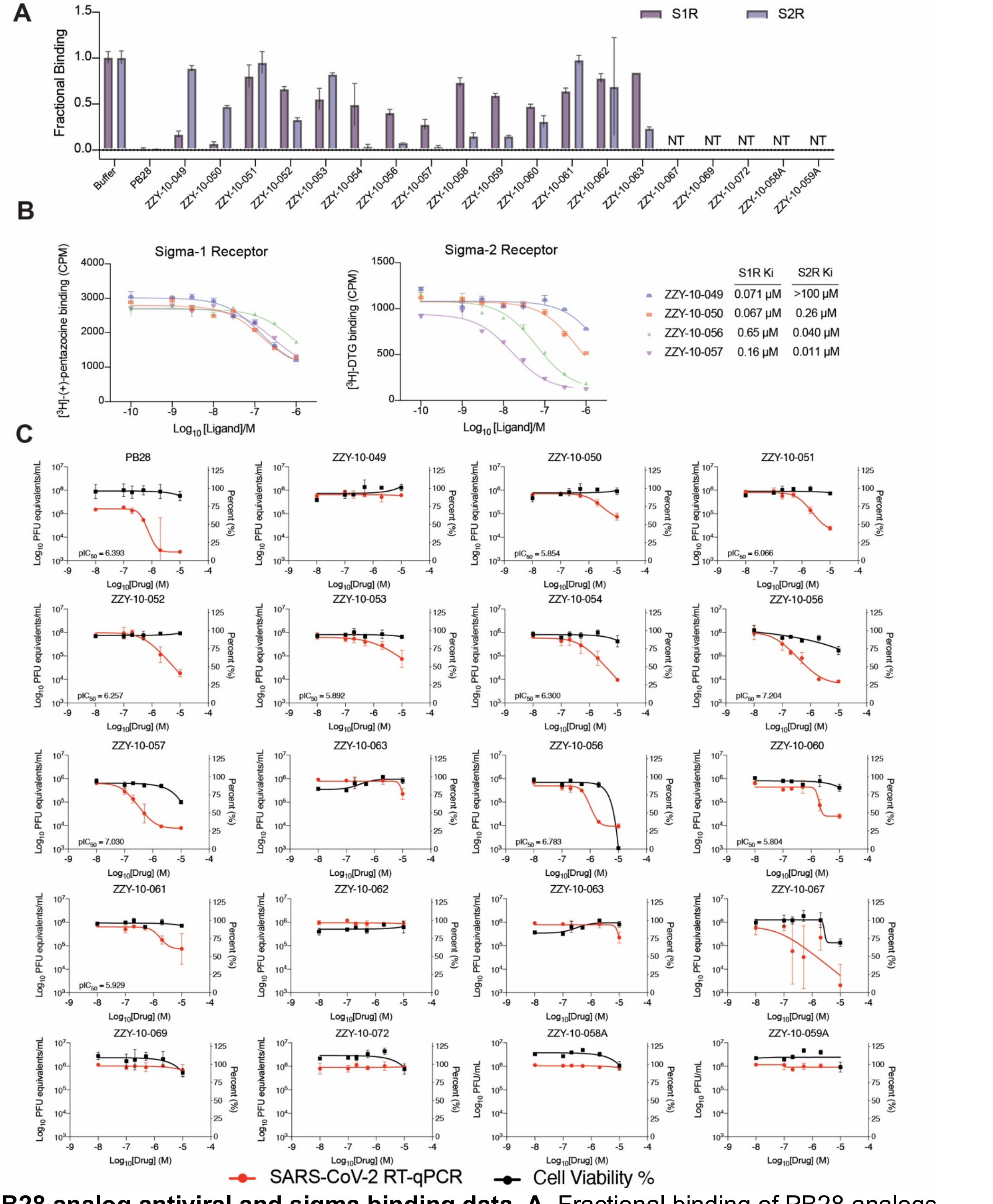

Fig S8. PB28 analog antiviral and sigma binding data. A. Fractional binding of PB28 analogs against Sigma-1 (purple) and Sigma-2 (blue) normalized to a buffer control at 1.0 in a radioligand binding experiment. Data shown are mean ± SEM from three biological replicates. **B.** Dose-response curves for selected PB28 analogs in Sigma-1 and Sigma-2 radioligand competition binding assay. Data points shown are mean ± SEM from three biological replicates. **C.** Viral infectivity data for PB28 analogs A549-ACE2 cells. Data shown are mean ± SD from three biological replicates.

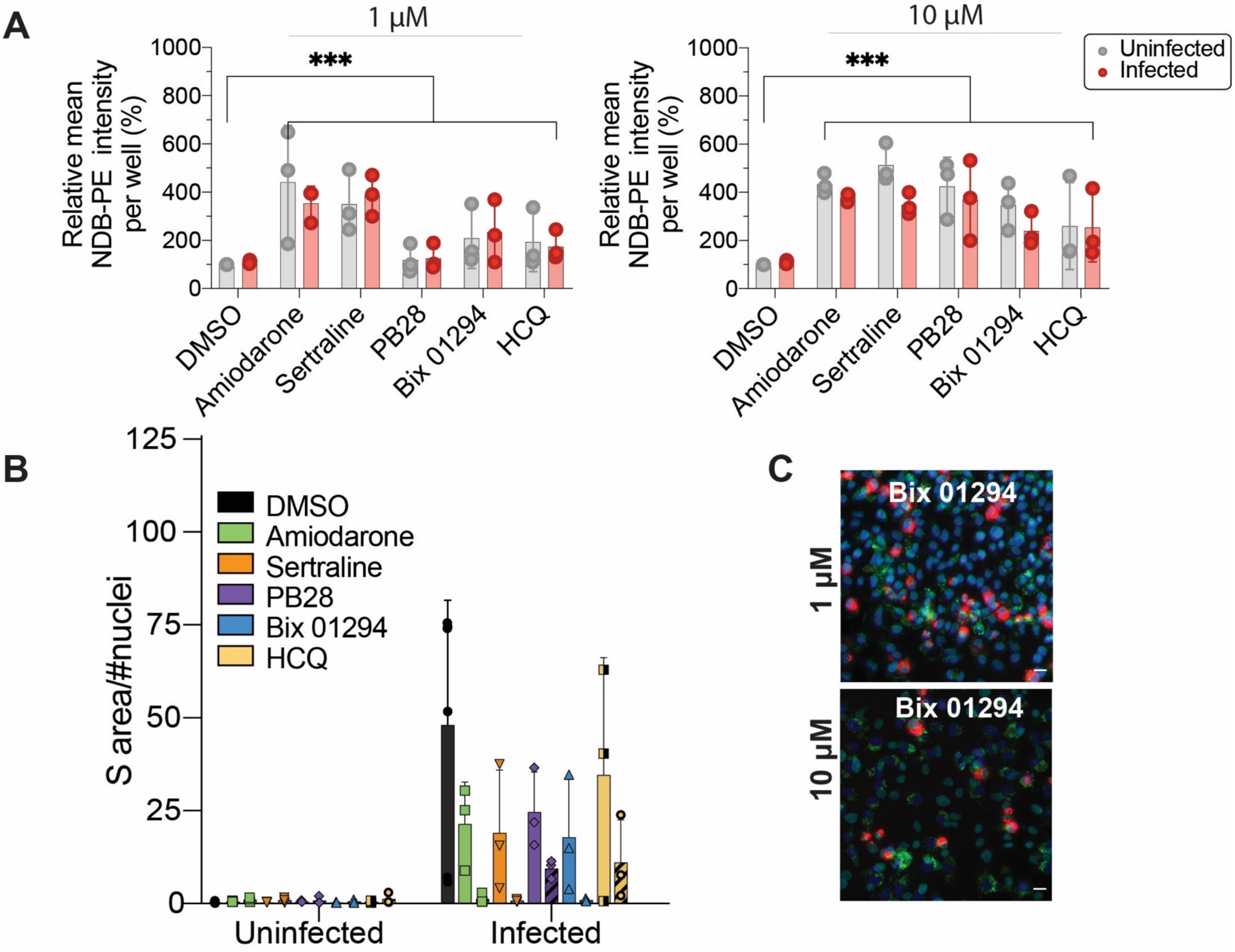

Fig S9. Quantification of phospholipidosis and spike protein in the same cells. A. Relative mean ± SD NBD-PE intensity per well percent for 5 molecules and DMSO (1 and 10 μM) in uninfected and SARS-CoV-2 infected A549-ACE2 cells. Data shown are pooled from three independent experiments each in biological quadruplicate. Two-way ANOVA main effect of drug treatment at 1 μM, $F(5, 24) = 7.7$, ***$P < 0.001$, no main effect of infection state, $F(1,24) = 0.02$, $P=0.90$, and 10 μM, $F(5, 24) = 9.1$, ***$P < 0.001$, no main effect of infection state, $F(1,24) = 3.48$, $P=0.07$. **B.** Spike protein quantification in the same experiment as **A.** for both uninfected and SARS-CoV-2 infected cells, and 1 (solid color bars) and 10 μM (hatched bars) drug treatments. Data represent mean ± SD from three independent experiments each in biological quadruplicate. Spike protein was quantified as S area / # nuclei per well. **C.** Example images from the costaining experiment measuring phospholipidosis and SARS-CoV-2 Spike protein in infected and uninfected A549-ACE2 cells. Bix 01294 (1 and 10 μM) is shown. Blue = Hoechst nuclei staining; Red = Spike protein staining; Green = NBD-PE phospholipid staining; Yellow = coexpression of spike protein and NBD-PE. Scale bar = 20 μm.

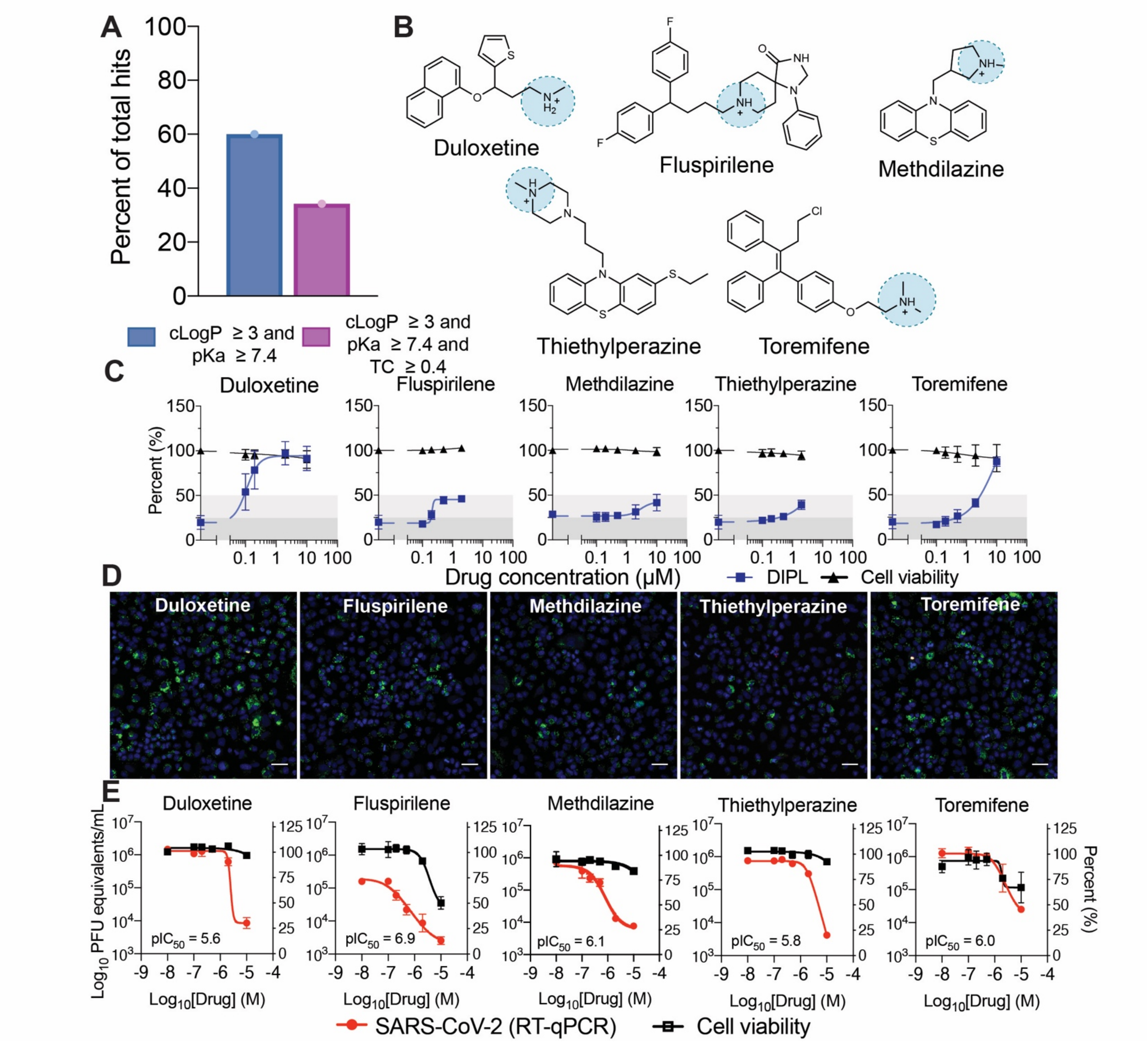

Fig. S10. Many drugs with activity against SARS-CoV-2 are CADs that induce phospholipidosis. A. Percentage of total drug repurposing hits collected that pass CAD thresholds. **B.** Example repurposing hits from the literature that pass our CAD filters. **C.** Dose response curves for five predicted phospholipidosis inducers. All five induce measurable phospholipidosis (blue) with no impact on cell viability (black). **D.** Representative images of phospholipidosis quantification through NBD-PE staining in A549 cells. Blue = Hoechst nuclei staining, Green = NBD-PE phospholipid staining, Red = EthD-2 staining for dead cells. Scale bars = 20 μm. **E.** Viral infectivity (red) and cytotoxicity (black) data for five example literature CADs tested in A549-ACE2 cells. Data shown are mean ± SD from three biological replicates.

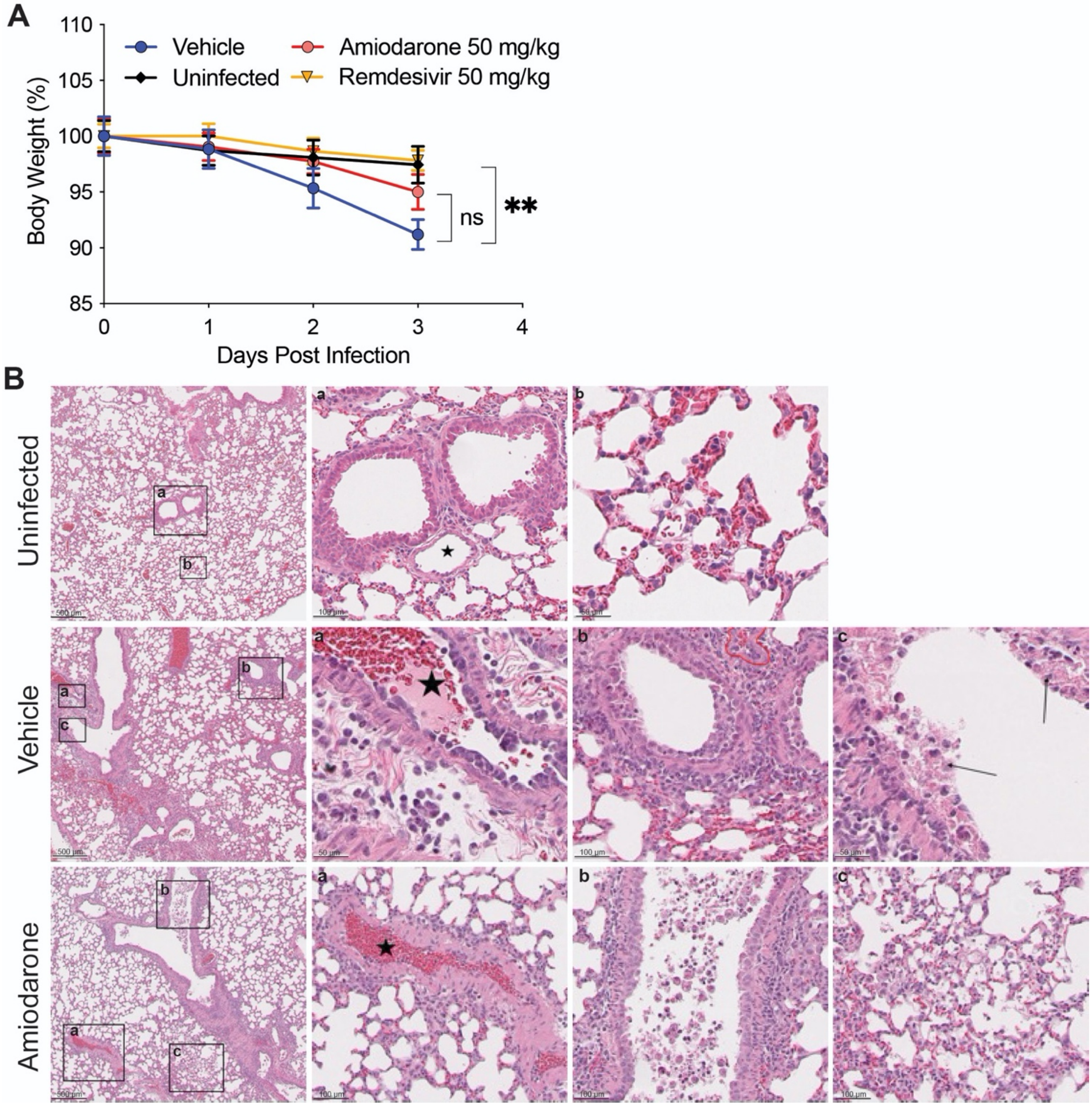

Fig S11. Additional endpoints for 15-day dosing experiment. A. Uninfected mice, vehicle-treated infected mice, and drug-treated mice were weighed over the course of three days after SARS-CoV-2 infection. Groups were compared with a two-way ANOVA (main effect of treatment $F(3,76) = 2.98$, $P = 0.04$; main effect of day post-infection, $F(3,76) = 8.22$, $P < 0.0001$; no interaction between variables, $F(9,76) = 1.03$, $P = 0.42$). Individual group comparisons determined using Dunnett's multiple comparison test are indicated (day 3: vehicle $N = 6$ vs. uninfected $N = 6$, $^{**}P = 0.006$; vehicle vs. remdesivir $N = 6$, $^{**}P = 0.003$; vehicle vs. amiodarone $N = 5$, ns = not significant, $P = 0.17$). Data are mean ± SEM. **B.** Lungs were harvested on day 3 post-infection and stained for hematoxylin and eosin. Regions of the lung anatomy where inflammation was assessed are highlighted by black boxes, with the corresponding higher-magnification image indicated by matching letter. Regions where inflammation was detected are indicated by arrows. Quantification of histopathology scores are shown in **Fig. 5**.

Table S3. Cationic amphiphilic drugs found active against other viruses in the literature.

Compound	ZINCID	SMILES	Virus	Antiviral Assay	Activity Type	Activity Value (uM)	Antiviral Reference
Amiodarone	ZINC3830212	CCCCc1oc2ccccc2c1C(=O)c1cc(I)c(OCCN(CC)CC)c(I)c1	EBOV	VeroE6 EBOV-eGFP assay	EC50	7.6	27622822
			MARV	EAhy MARV GP pseudoparticle assay	EC50	1.18 ug/mL	24710028
			SARS	VeroE6 SARS CPE assay	IC50	4.18	33060197
			HCV	Huh-7.5.1 luc-IRES assay	IC50	2.1	23659500
Amodiaquine	ZINC608172	CCN(CC)Cc1cc(Nc2ccnc3cc(Cl)ccc23)ccc1O	EBOV	VeroE6 EBOV-eGFP assay	EC50	34	27622822
			MERS	VeroE6 MERS ELISA	EC50	6.212	24841273
			SARS	VeroE6 SARS CPE assay	EC50	1.274	24841273
Aripiprazole	ZINC1851149	O=C1CCc2ccc(OCCCCN3CCN(c4cccc(Cl)c4Cl)CC3)cc2N1	EBOV	VeroE6 eGFP-EBOV assay	IC50	8.1	26041706
Astemizole	ZINC601274	COc1ccc(CCN2CCC(Nc3nc4ccccc4n3Cc3ccc(F)cc3)CC2)cc1	EBOV	VeroE6 eGFP-EBOV assay	IC50	6.17	26041706
Benztropine	ZINC100036536	CN1[C@H]2CC[C@@H]1C[C@H](OC(c1ccccc1)c1ccccc1)C2	EBOV	VeroE6 eGFP-EBOV assay	IC50	8.07	26041706
			MERS	VeroE6 MERS ELISA	EC50	16.627	24841273
			SARS	VeroE6 SARS CPE assay	EC50	21.611	24841273
			EBOV	A549 HIV/EBOV pseudotyped virus	IC50	3.7	26202243
			MARV	A549 HIV/MARV pseudotyped virus + luciferase reporter gene assay	IC50	13.2	26202243

			EBOV	VeroE6 EBOV-eGFP assay	EC50	9.2	27622822
Chlorcyclizine		Clc1ccc(cc1)C(c2ccccc2)N3CCN(CC3)C	HCV	Huh7.5.1 HCV RT-qPCR assay	EC50	0.0331	25855495
Chloroquine	ZINC19144226	CCN(CC)CCC[C@@H](C)Nc1ccnc2cc(Cl)ccc12	MERS	VeroE6 MERS ELISA	EC50	6.275	24841273
			SARS	VeroE6 SARS CPE assay	EC50	6.538	24841273
			EBOV	VeroE6 EBOV-eGFP assay	EC50	16	27622822
Chlorpromazine	ZINC44027	CN(C)CCCN1c2ccccc2Sc2ccc(Cl)cc21	MERS	VeroE6 MERS ELISA	EC50	9.514	24841273
			SARS	VeroE6 SARS CPE assay	EC50	12.971	24841273
Clemastine	ZINC402830	CN1CCC[C@@H]1CCO[C@](C)(c1ccccc1)c1ccc(Cl)cc1	EBOV	VeroE6 eGFP-EBOV assay	IC50	5.44	26041706
			EBOV	VeroE6 EBOV-eGFP assay	EC50	5.2	27622822
Clomiphene	ZINC1530601	CCN(CC)CCOc1ccc(/C(=C(/Cl)c2ccccc2)c2ccccc2)cc1	EBOV	VeroE6 eGFP-EBOV assay	IC50	2.42	26041706
			EBOV	VeroE6 EBOV-eGFP assay	EC50	11	27622822
Clomipramine	ZINC20248	CN(C)CCCN1c2ccccc2CCc2ccc(Cl)cc21	EBOV	VeroE6 eGFP-EBOV assay	IC50	11.4	26041706
			MERS	VeroE6 MERS ELISA	EC50	9.332	24841273
			SARS	VeroE6 SARS CPE assay	EC50	13.238	24841273
Fluphenazine	ZINC19203912	OCCN1CCN(CCCN2c3ccccc3Sc3ccc(C(F)(F)F)cc32)CC1	EBOV	VeroE6 eGFP-EBOV assay	IC50	5.54	26041706
			MERS	VeroE6 MERS ELISA	EC50	5.868	24841273
			SARS	VeroE6 SARS CPE assay	EC50	21.431	24841273

			EBOV	VeroE6 EBOV-eGFP assay	EC50	12	27622822
Fluspirilene	ZINC537755	O=C1NCN(c2ccccc2)C12CCN(CCCC(c1ccc(F)cc1)c1ccc(F)cc1)CC2	MERS	VeroE6 MERS ELISA	EC50	7.477	24841273
			SARS	VeroE6 SARS CPE assay	EC50	5.963	24841273
Hydroxychloroquine	ZINC1530652	CCN(CCO)CCC[C@@H](C)Nc1ccnc2cc(Cl)ccc12	MERS	VeroE6 MERS ELISA	EC50	8.279	24841273
			SARS	VeroE6 SARS CPE assay	EC50	7.966	24841273
			EBOV	VeroE6 EBOV-eGFP assay	EC50	22	27622822
Hydroxyzine	ZINC19364222	OCCOCCN1CCN([C@@H](c2ccccc2)c2ccc(Cl)cc2)CC1	HCV	Huh7.5.1 HCV RT-qPCR assay	EC50	0.0503	25855495
Maprotiline	ZINC1530688	CNCCCC12CCC(c3ccccc31)c1ccccc12	EBOV	VeroE6 eGFP-EBOV assay	IC50	9.63	26041706
Paroxetine	ZINC527386	Fc1ccc([C@@H]2CCNC[C@H]2COc2ccc3c(c2)OCO3)cc1	EBOV	VeroE6 eGFP-EBOV assay	IC50	7.45	26041706
			EBOV	VeroE6 EBOV-eGFP assay	EC50	27	27622822
Pimozide	ZINC4175630	Oc1nc2ccccc2n1C1CCN(CCCC(c2ccc(F)cc2)c2ccc(F)cc2)CC1	EBOV	VeroE6 eGFP-EBOV assay	IC50	3.12	26041706
Prochlorperazine	ZINC19796018	CN1CCN(CCCN2c3ccccc3Sc3ccc(Cl)cc32)CC1	EBOV	VeroE6 eGFP-EBOV assay	IC50	5.96	26041706
			EBOV	VeroE6 EBOV-eGFP assay	EC50	11	27622822
Promazine	ZINC10402	CN(C)CCCN1c2ccccc2Sc2ccccc21	EBOV	VeroE6 EBOV-eGFP assay	EC50	21	27622822
Promethazine	ZINC20250	C[C@@H](CN1c2ccccc2Sc2	MERS	VeroE6 MERS ELISA	EC50	11.802	24841273

		ccccc21)N(C)C					
			SARS	VeroE6 SARS CPE assay	EC50	7.545	24841273
			EBOV	A549 HIV/EBOV pseudotyped virus + luciferase reporter gene assay	IC50	19.4	26202243
			MARV	A549 HIV/MARV pseudotyped virus + luciferase reporter gene assay	IC50	19.1	26202243
Sertraline	ZINC1853550	CN[C@H]1CC[C@@H](c2ccc(Cl)c(Cl)c2)c2ccccc21	EBOV	VeroE6 eGFP-EBOV assay	IC50	3.13	26041706
Tamoxifen	ZINC1530689	CC/C(=C(\c1ccccc1)c1ccc(OCCN(C)C)cc1)c1ccccc1	MERS	VeroE6 MERS ELISA	EC50	10.117	24841273
			SARS	VeroE6 SARS CPE assay	EC50	92.886	24841273
			EBOV	VeroE6 EBOV-eGFP assay	EC50	3	27622822
Thi-ethylperazine	ZINC22446674	CCSc1ccc2c(c1)N(CCCN1CCN(C)CC1)c1ccccc1S2	MERS	VeroE6 MERS ELISA	EC50	7.865	24841273
Thioridazine	ZINC1530695	CSc1ccc2c(c1)N(CC[C@@H]1CCCCN1C)c1ccccc1S2	EBOV	VeroE6 eGFP-EBOV assay	IC50	6.24	26041706
Toremifene	ZINC12404516	CN(C)CCOc1ccc(/C(=C(/CCCl)c2ccccc2)c2ccccc2)cc1	EBOV	VeroE6 eGFP-EBOV assay	IC50	0.162	26041706
			MERS	VeroE6 MERS ELISA	EC50	12.915	24841273
			SARS	VeroE6 SARS CPE assay	EC50	11.969	24841273
Triflupromazine	ZINC538507	CN(C)CCCN1c2ccccc2Sc2ccc(C(F)(F)F)cc21	MERS	VeroE6 MERS ELISA	EC50	5.758	24841273
			SARS	VeroE6 SARS CPE assay	EC50	6.398	24841273

Trimipramine	ZINC968275	C[C@H](CN(C)C)CN1c2ccccc2CCc2ccccc21	EBOV	A549 HIV/EBOV pseudotyped virus + luciferase reporter gene assay	IC50	11.1	26202243
			MARV	A549 HIV/MARV pseudotyped virus + luciferase reporter gene assay	IC50	10.9	26202243
Triparanol	ZINC1692389	CCN(CC)CCOc1ccc([C@](O)(Cc2ccc(Cl)cc2)c2ccc(C)cc2)cc1	MERS	VeroE6 MERS ELISA	EC50	5.283	24841273
			EBOV	VeroE6 EBOV-eGFP assay	IC50	1.92	23441171
U 18666A	ZINC118915627	[H][C@@]23CC=C1C[C@@H](OCCN(CC)CC)CC[C@@](C)1[C@]([H])2CC[C@@]4(C)[C@]([H])3CCC4=O	EBOV	VeroE6 EBOV-eGFP assay	IC50	8.05	23441171
			DENV	A549 dengue replicon assay	EC50	6.2	22146564

Table S4. Measured pharmacokinetic parameters for Amiodarone.

**Amiodarone**		**Dose Route**	**Dose (mg/kg)**	**T_{max} (min)**	**Cmax, ng/mL (g)**	**$AUC_{0\rightarrow tlast}$ ng*min/mL (g)**	**$AUC_{0\rightarrow\infty}$ ng*min/ mL (g)**	**$T_{1/2}$ (min)**	**K_{el} (min^{-1})**	**Cmax (nM)**
MWT (Da) 681.8	Plasma	i.p.[a]	0.3	30	37.4	18800	34200	1270	0.000548	55
		i.p.	1	30	73.5	42000	52500	583	0.00119	108
		i.p.	3	30	178	96700	144000	769	0.000901	261
		i.p.	10	15	519	205000	278000	585	0.0118	761
		i.p.	30	60	1070	476000	577000	505	0.00137	1569
	Lung	i.p.	0.3	360	120	121000	162000	698	0.000993	176
		i.p.	1	120	328	310000	375000	599	0.00116	481
		i.p.	3	120	1240	1110000	1230000	501	0.00138	1819
		i.p.	10	120	8020	3650000	4870000	645	0.00107	11763
		i.p.	30	120	13900	8930000	17900000	1250	0.000554	20387

[a]i.p. = intraperitoneally

Table S5. Measured pharmacokinetic parameters for Sertraline.

Sertraline		Dose Route	Dose (mg/kg)	T_{max} (min)	Cmax, ng/mL (g)	$AUC_{0\rightarrow tlast}$ ng*min/mL (g)	$AUC_{0\rightarrow\infty}$ ng*min/mL (g)	$T_{1/2}$ (min)	K_{el} (min^{-1})	Cmax (nM)
MWT (Da) 306.2	Plasma	i.p.[a]	0.3	15	11.8	979	10400	626	0.00111	39
		i.p.	1	30	18.4	1750	3730	123	0.00565	60
		i.p.	3	30	79	11500	14600	147	0.00472	258
		i.p.	10	30	332	41100	44700	103	0.00672	1084
		i.p.	30	60,0	902	193000	198000	272	255	2945
	Lung	i.p.	0.3	60	949	163000	197000	134	0.00518	3099
		i.p.	1	60	3290	576000	845000	200	0.00347	10743
		i.p.	3	30	11500	3270000	3320000	257	0.0027	37553
		i.p.	10	30	29100	6700000	6740000	198	0.0035	95025
		i.p.	30	15	43500	14500000	14500000	188	0.00369	14204 7

[a]i.p. = intraperitoneally

Table S6. Measured pharmacokinetic parameters for Tamoxifen.

Tamoxifen		Dose Route	Dose (mg/kg)	T_{max} (min)	Cmax, ng/mL (g)	$AUC_{0\rightarrow tlast}$ ng*min/mL (g)	$AUC_{0\rightarrow\infty}$ ng*min/ mL (g)	$T_{1/2}$ (min)	K_{el} (min^{-1})	Cmax (nM)
MWT (Da) 371.5	Plasma	i.p.[a]	0.3	ND[b]	ND	ND	ND	ND	ND	NA
		i.p.	1	120	13.7	4030	23100	1120	0.00062	37
		i.p.	3	120	49.4	13600	36700	464	0.00149	133
		i.p.	10	120	105	31400	42100	669	0.00104	283
		i.p.	30	360	436	409000	493000	538	0.00129	1174
	Lung	i.p.	0.3	360	174	147000	152000	278	0.00249	468
		i.p.	1	360	639	572000	609000	348	0.00199	1720
		i.p.	3	120	1970	1640000	1770000	398	0.00174	5303
		i.p.	10	360	4950	4390000	4650000	337	0.00205	13324
		i.p.	30	360	33400	28200000	31800000	430	0.00161	89902

[a]i.p. = intraperitoneally
[b]ND = not determined

Table S7. Measured pharmacokinetic parameters for PB28.

PB28		Dose Route	Dose (mg/kg)	T_{max} (min)	Cmax, ng/mL (g)	$AUC_{0 \to tlast}$ ng*min/mL (g)	$AUC_{0 \to \infty}$ ng*min/mL (g)	$T_{1/2}$ (min)	K_{el} (min^{-1})	Cmax (nM)
MWT (Da) 370.6	Plasma	i.p.[a]	0.3	30	15.8	269	ND[b]	ND	ND	43
		i.p.	1	30	16.4	3260	4690	200	0.00347	44
		i.p.	3	15	25.1	5760	7120	169	0.00411	68
		i.p.	10	15	89.6	13700	29700	431	0.00161	242
		i.p.	30	5	336	41900	56300	191	0.00362	907
	Lung	i.p.	0.3	15	206	37600	50800	188	0.00368	556
		i.p.	1	30	735	116000	144000	150	0.00463	1983
		i.p.	3	15	2500	316000	367000	130	0.00535	6746
		i.p.	10	15	6720	2120000	2130000	218	0.00318	18134
		i.p.	30	5	20600	7350000	7380000	186	0.00373	55590

[a]i.p. = intraperitoneally
[b]ND = not determined

Table S8. Measured pharmacokinetic parameters for Elacridar.

Elacridar		Dose Route	Dose (mg/kg)	T_{max} (min)	Cmax, ng/mL (g)	$AUC_{0\rightarrow tlast}$ ng*min/mL (g)	$AUC_{0\rightarrow\infty}$ ng*min/mL (g)	$T_{1/2}$ (min)	K_{el} min^{-1}	Cmax (nM)
MWT (Da) 563.7	Plasma	i.p.[a]	0.3	120	42.4	26900	29900	437	0.00159	75
		i.p.	1	60	78.9	57900	80300	790	0.000878	140
		i.p.	3	120	182	141000	155000	427	0.00162	323
		i.p.	10	360	489	511000	752000	836	0.000829	868
		i.p.	30	360	717	802000	1400000	1120	0.000618	1272
	Lung	i.p.	0.3	60	507	286000	350000	604	0.00115	899
		i.p.	1	60	1560	764000	864000	478	0.00145	2768
		i.p.	3	60	4090	2550000	2650000	330	0.0021	7256
		i.p.	10	60	13300	10600000	12600000	571	0.00121	23596
		i.p.	30	120	18700	17900000	37500000	1470	0.000471	33176

[a]i.p. = intraperitoneally

Table S9. Estimates of expenditures of COVID-19 cationic amphiphilic drug clinical trials.

Phase	Mean cost[a]	Number of CAD trials[b]	Cost of CAD trials	Number of non-CAD HCQ/CQ trials	Cost of non-CAD HCQ/CQ trials	Number of non HCQ/CQ CAD trials[c]	Cost of non-HCQ/CQ CAD trials	Number of small molecule trials	Cost of small molecule trials	Percentage CAD trials versus all trials
0		9		4		5		58		
1	$4.2	13	$55	7	$29	6	$25	56	$235	23%
2	$14.2	99	$1406	48	$682	51	$724	409	$5808	24%
3	$22.8	162	$3694	98	$2234	64	$1459	428	$9758	38%
1/2[e]	$14.2	4	$57	2	$28	2	$28	40	$568	10%
2/3[e]	$22.8	38	$866	25	$570	13	$296	125	$2850	30%
3/4[e]	$11	0						3	$33	
4	$11	68	$748	45	$495	23	$253	154	$1694	44%
Not available		46		29		17		217		21%
Phase I to III Totals		316	$6078	180	$3544	136	$2533	1058	$19219	30%
Phase I to IV Totals		442	$6826	258	$4039	181	$2786	1490	$20946	29%

[a]All costs are in US millions of dollars, not accounting for inflation since the time of the study (*50, 51*)
[b]Cationic amphiphilic drugs (CADs) estimated using cLogP ≥ 3 and pKa ≥ 7.4 criteria; trials using azithromycin, a non-CAD PLD inducer, is included in these numbers; trials are only counted one time if more than one CAD was administered in a trial
[c]Trials that administered a CAD in addition to HCQ or CQ are included in these numbers
[e] Costs for mixed-phase trials were estimated to be the cost of only the higher phase trial

References

54. L. M. Wingler, C. McMahon, D. P. Staus, R. J. Lefkowitz, A. C. Kruse, Distinctive activation mechanism for angiotensin receptor revealed by a synthetic nanobody. *Cell* **176**, 479-490.e12 (2019).
55. J. Buchrieser, J. Dufloo, M. Hubert, B. Monel, D. Planas, M. M. Rajah, C. Planchais, F. Porrot, F. Guivel-Benhassine, S. Van der Werf, N. Casartelli, H. Mouquet, T. Bruel, O. Schwartz, Syncytia formation by SARS-CoV-2-infected cells. *EMBO J.* **39**, e106267 (2020).
56. F. Amanat, K. M. White, L. Miorin, S. Strohmeier, M. McMahon, P. Meade, W. Liu, R. A. Albrecht, V. Simon, L. Martinez-Sobrido, T. Moran, A. García-Sastre, F. Krammer, An in vitro microneutralization assay for SARS-CoV-2 serology and drug screening. *Current Protocols in Microbiology* **58**, e108 (2020).
57. D. Planas, T. Bruel, L. Grzelak, F. Guivel-Benhassine, I. Staropoli, F. Porrot, C. Planchais, J. Buchrieser, M. M. Rajah, E. Bishop, M. Albert, F. Donati, M. Prot, S. Behillil, V. Enouf, M. Maquart, M. Smati-Lafarge, E. Varon, F. Schortgen, L. Yahyaoui, M. Gonzalez, J. De Sèze, H. Péré, D. Veyer, A. Sève, E. Simon-Lorière, S. Fafi-Kremer, K. Stefic, H. Mouquet, L. Hocqueloux, S. van der Werf, T. Prazuck, O. Schwartz, Sensitivity of infectious SARS-CoV-2 B.1.1.7 and B.1.351 variants to neutralizing antibodies. *Nat. Med.* **27**, 917–924 (2021).
58. F. Berardi, C. Abate, S. Ferorelli, V. Uricchio, N. A. Colabufo, M. Niso, R. Perrone, Exploring the importance of piperazine N-atoms for sigma(2) receptor affinity and activity in a series of analogs of 1-cyclohexyl-4-[3-(5-methoxy-1,2,3,4-tetrahydronaphthalen-1-yl)propyl]piperazine (PB28). *J. Med. Chem.* **52**, 7817–7828 (2009).
59. P.-C. Bürkner, brms: An R package for Bayesian multilevel models using Stan. *Journal of Statistical Software* **80**, 1–28 (2017).

60. A. Vehtari, A. Gelman, J. Gabry, Practical Bayesian model evaluation using leave-one-out cross-validation and WAIC. *Stat. Comput*. **27**, 1413–1432 (2017).

61. S. Kim, J. Chen, T. Cheng, A. Gindulyte, J. He, S. He, Q. Li, B. A. Shoemaker, P. A. Thiessen, B. Yu, L. Zaslavsky, J. Zhang, E. E. Bolton, PubChem in 2021: New data content and improved web interfaces. *Nucleic Acids Res*. **49**, D1388–D1395 (2021).

62. R. Rathnasinghe, S. Strohmeier, F. Amanat, V. L. Gillespie, F. Krammer, A. García-Sastre, L. Coughlan, M. Schotsaert, M. B. Uccellini, Comparison of transgenic and adenovirus hACE2 mouse models for SARS-CoV-2 infection. *Emerg. Microbes Infect.* **9**, 2433–2445 (2020).

63. L. Coughlan, A. C. Bradshaw, A. L. Parker, H. Robinson, K. White, J. Custers, J. Goudsmit, N. Van Roijen, D. H. Barouch, S. A. Nicklin, A. H. Baker, Ad5:Ad48 hexon hypervariable region substitutions lead to toxicity and increased inflammatory responses following intravenous delivery. *Mol. Ther.* **20**, 2268–2281 (2012).

64. L. Coughlan, S. Vallath, A. Saha, M. Flak, I. A. McNeish, G. Vassaux, J. F. Marshall, I. R. Hart, G. J. Thomas, In vivo retargeting of adenovirus type 5 to αvβ6 integrin results in reduced hepatotoxicity and improved tumor uptake following systemic delivery. *J. Virol*. **83**, 6416–6428 (2009).

65. R. Rathnasinghe, S. Jangra, A. Cupic, C. Martínez-Romero, L. C. F. Mulder, T. Kehrer, S. Yildiz, A. Choi, I. Mena, J. De Vrieze, S. Aslam, D. Stadlbauer, D. A. Meekins, C. D. McDowell, V. Balaraman, J. A. Richt, B. G. De Geest, L. Miorin, F. Krammer, V. Simon, A. García-Sastre, M. Schotsaert, The N501Y mutation in SARS-CoV-2 spike leads to morbidity in obese and aged mice and is neutralized by convalescent and post-vaccination human sera (2021), (available at http://dx.doi.org/10.1101/2021.01.19.21249592).

66. D. S. Himmelstein, User-friendly extensions of the DrugBank database v1.0 (2016; https://zenodo.org/record/45579).
